# Genotype epigenome phenotype integration reveals peripheral immune contributions to type I bipolar disorder

**DOI:** 10.1038/s41467-026-72543-0

**Published:** 2026-05-15

**Authors:** Lei Hou, Yue Li, Xushen Xiong, Yosuke Tanigawa, Yongjin Park, Samuel W. Lenz, Amy Grayson, Jeong-Heon Lee, Euijung Ryu, Janet E. Olson, Joanna M. Biernacka, Mark A. Frye, Tamas Ordog, Manolis Kellis

**Affiliations:** 1 Computer Science and Artificial Intelligence Lab, Massachusetts Institute of Technology, Cambridge, MA USA; 2 The Broad Institute of Harvard and MIT, Cambridge, MA USA; 3 Department of Medicine, Biomedical Genetics Section, Boston University School of Medicine, Boston, MA USA; 4 Department of Molecular Oncology, BC Cancer, Vancouver, BC Canada; 5 Center for Individualized Medicine, Mayo Clinic, Rochester, MN USA; 6 Division of Experimental Pathology and Laboratory Medicine, Department of Laboratory Medicine and Pathology, Mayo Clinic, Rochester, Minnesota USA; 7 Department of Quantitative Health Sciences, Mayo Clinic, Rochester, MN USA; 8 Department of Psychiatry & Psychology, Mayo Clinic, Rochester, MN USA; 9 Department of Physiology and Biomedical Engineering, Mayo Clinic, Rochester, MN USA; 10 Division of Gastroenterology and Hepatology, Department of Medicine, Mayo Clinic, Rochester, MN USA; 11 Present Address: School of Computer Science, McGill University, Montreal, QC Canada; 12 Present Address: Department of Pathology and Laboratory Medicine, Department of Statistics, University of British Columbia, Vancouver, BC Canada

**Keywords:** Genetics research, Computational biology and bioinformatics, Neuroimmunology, Molecular medicine, Diagnostic markers

## Abstract

Immune dysfunction has been increasingly implicated in bipolar disorder, but the underlying mechanisms remain unclear. To address this, we profile 833 genome-wide chromatin immunoprecipitation sequencing datasets spanning five histone marks in peripheral blood immune cells from 88 Type I bipolar disorder patients and 92 controls, integrating them with whole-genome sequencing and clinical data. We identify disease-associated *cis*-regulatory elements and genetically influenced regulatory elements, revealing immune signatures and pathways involving calcium signaling and endoplasmic reticulum transport. By integrating genetic risk variants, differential and genetically influenced regulatory elements, and regulatory element—gene links, we prioritize 39 driver genes, 28 of which are exclusively supported by blood evidence. We further stratify patients into five epigenomic subtypes with distinct clinical features and genetic risk profiles and identify compounds that reverse disease-associated epigenomic signatures. Here, we combine immune epigenomics with genetics and clinical traits to identify driver genes, patient subtypes, and therapeutic candidates, highlighting immune contributions to type I bipolar disorder pathogenesis.

## Abstract (web summary)

Immune dysfunction is implicated in bipolar disorder, but mechanisms are unclear. Here, the authors map epigenomic changes in peripheral blood from patients with bipolar disorder and link them to genetic risk and regulatory networks, revealing new disease mechanisms, patient subtypes, and potential therapeutic targets.

## Introduction

The substantial global burden of bipolar disorder (BD), schizophrenia (SCZ), and other psychiatric diseases represents a significant public health concern^1^. These disorders often require lifelong treatment and are characterized by high patient-to-patient heterogeneity in symptoms, disease progression, and therapeutic response^2–4^ and temporal variability of disease course^5^. Contributing factors include diverse genetic backgrounds, environmental exposures, and comorbid conditions, which together complicate early diagnosis and hinder the tracking of disease progression and the development of targeted interventions. To advance our understanding of BD pathogenesis and to improve patient outcomes, two interdependent efforts are essential: (1) to elucidate the diverse genetic and non-genetic factors that underlie BD pathogenesis and treatment response; and (2) identify molecular signatures that can stratify patients into biologically meaningful subtypes predictive of physiological states and therapeutic outcomes.

Although psychiatric drug development has traditionally focused on the nervous system, a growing body of evidence implicates immune dysregulation as a mechanistic contributor to psychiatric disease. From a genetic perspective, genome-wide association studies (GWAS) have revealed tens to hundreds of loci associated with different psychiatric disorders, including BD^6^, SCZ^7^, and major depressive disorder (MDD)^8^. Interestingly, besides top genetic hits for BD directly impacting neuronal activity, including ion channels, synapse function, and neurotransmitters, GWAS has also revealed that immune function genes may be causal contributors to psychiatric disorders^9–11^, with driver genetic loci associated with multiple psychiatric disorders enriched in immune-related pathways and genomic regions active in immune cell types^12,13^. These disorders also show a strong genetic correlation with inflammatory or autoimmune diseases and inflammatory biomarkers in blood, such as C-reactive protein (CRP)^14^. At the cellular level, microglia, a resident immune cell type in the brain, is considered central for brain homeostasis as their dysregulation may lead to neuroinflammation^15^, a contributing factor to psychiatric diseases^16^. Besides the brain immune system, there is an emerging role of circulating immune cells in psychiatric diseases, which communicate with the brain through cytokine and chemokine signaling^17^. Multiple studies have reported alterations of immune functions in peripheral blood from BD, SCZ, and MDD patients, including T cell exhaustion and T cell senescence^18,19^. Common BD medicines, e.g., aripiprazole, topiramate, and quetiapine, could lower inflammation in peripheral blood, indicating a potential mechanism of action^20^. Immunomodulatory agents, such as non-steroidal anti-inflammatory drugs (NSAIDs) and cytokine inhibitors have also shown efficacy in subsets of MDD or BD patients with high baseline inflammation or a history of childhood adversity^21^, which is known to affect immune functions^22^. This evolving immune-psychiatry axis offers unique opportunities to uncover disease mechanisms, develop biomarkers from accessible tissues such as blood, and design individualized treatment strategies. However, it is unclear how BD genetic risk is implemented through immune cell *cis*-regulatory networks and whether circulating immune biomarkers could capture BD patient heterogeneity. In this study, we address this gap through large-scale integrative analysis of the immune epigenome, genetic variation, and clinical phenotypes in BD, revealing mechanistic insights into the role of circulating immune cells in psychiatric disease pathogenesis.

**Fig. 1:**
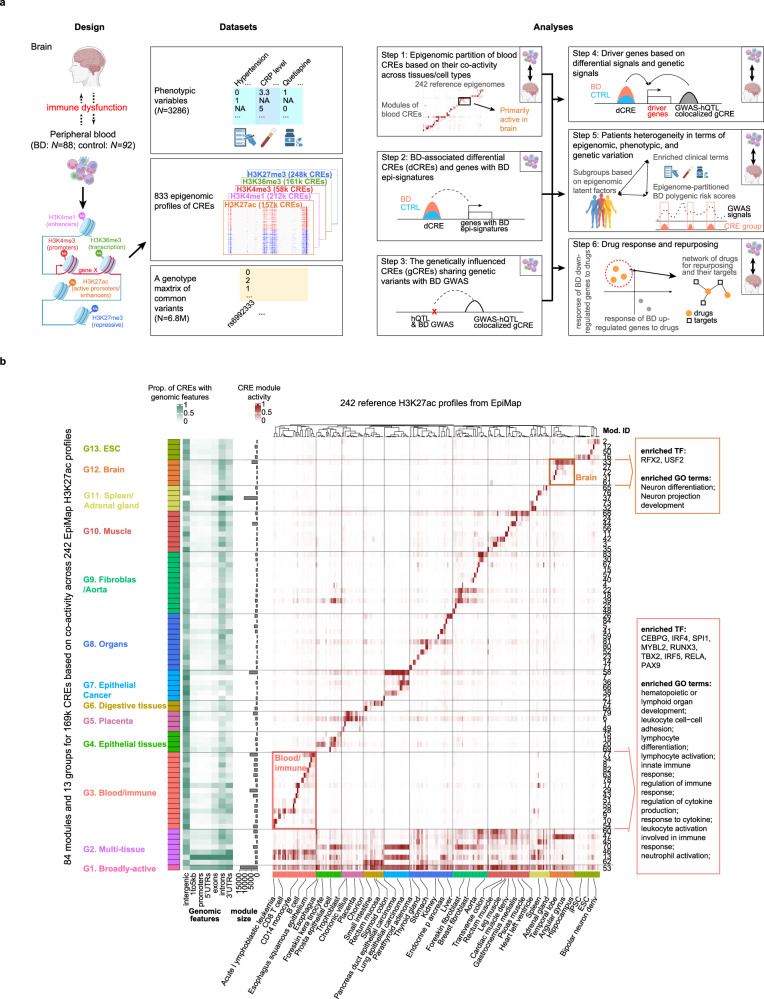
Overview of the study and epigenomic profiling. **a** Overview of the integrative analyses of genotypic, blood epigenomic, and phenotypic data for type I bipolar disorder (BD). Symbols created with BioRender (Brain and blood symbols were created in BioRender. Hou, L. (2026) https://BioRender.com/bp5g9am, Multi-histone symbol was created in BioRender. Hou, L. (2026) https://BioRender.com/bugc8az, EHR symbol was created in BioRender. Hou, L. (2026) https://BioRender.com/n1wfbgb, Population symbol was created in BioRender. Hou, L. (2026) https://BioRender.com/jubqwp7). Panels from left to right: study design to reflect the connection between brain and blood; data generated including blood epigenomic, genotypic, and phenotypic data; and the integrative analyses consisting of six steps. CREs cis regulatory elements, dCREs differential CREs, gCREs genetically influenced CREs, hQTL histone quantitative trait locus, GWAS genome-wide association study. **b** 169k CREs associated with enhancers or promoters (H3K27ac, H3K4me1, and H3K4me3) in this study forming 13 groups of 84 modules based on coactivity across 242 reference epigenomes. Panels from left to right: 84 CRE modules; proportion of CREs annotated in different genomic features for each module; size of each module; average CRE activity (H3K27ac signal) of each module (by row) across 242 reference epigenomes (by column). Representative sample names labeled for each sample cluster on the bottom; IDs of 84 CRE modules labeled on the right; enrichment of TF and GO terms in brain and blood/immune groups are show in orange boxes on the right. TF transcription factor, GO gene ontology, ESC embryonic stem cell, UTR untranslated region. Source data are provided as a Source Data file.

We generated 833 epigenetic profiles for five histone modifications, namely H3K27ac, H3K4me1, H3K4me3, H3K36me3, and H3K27me3, in peripheral blood buffy coat samples from 180 participants including 88 type I BD patients (i.e., BD patients with manic and, usually, depressive episodes) and 92 age- and sex-matched controls without any psychiatric condition or disorder. We took six steps to analyze epigenomic variation by integrating these datasets with disease status, genetic variants from whole-genome sequencing (WGS) data, and risk factors and other health conditions from electronic health record (EHR) data across 3286 phenotypic variables for BD patients^23^ (Fig. 1a). (1) We first defined *cis*-regulatory elements (CREs) as peaks in ChIP-seq profiles and investigate co-activity patterns across multiple tissues. (2) We identified on average 8.4k CREs across the five histone marks (range: 805 for H3K4me1 and 22k for H3K27ac) showing differential activity between BD patients and controls (differential CREs, dCREs), suggesting overall increased activity of innate immune response and repression of adaptive immune response in BD. We inferred 2632 genes with BD-differential epigenomic signatures based on dCREs in gene bodies and gene regulatory domains defined by promoter capture Hi-C data. (3) We next mapped 1.8k–27k genetically influenced CREs (gCREs) implicating 1.6 M histone quantitative trait loci (hQTLs) in blood and identified 341 gCREs showing BD–GWAS–hQTL colocalization, which were enriched in genes related to calcium signaling and endoplasmic reticulum processes. (4) We then predicted 215 candidate BD driver genes acting in circulating immune cells based on BD GWAS signals, QTLs affecting CRE activity, CRE–gene links, and BD-differential epigenomic signatures. Of these 215 genes, 28 were confirmed by GWAS–eQTL colocalization in white blood cells only, whereas 11 were confirmed in both brain and blood cell types, suggesting an immune component in BD pathogenesis. (5) We also applied latent variable analysis to capture major epigenomic variation across individuals, clustered them based on latent variables into five subgroups with enriched clinical terms indicating BD comorbidity, risk factors, and preferred medicines. We found that these patient subtypes were significantly associated with several BD polygenic risk scores (PRSs), especially PRSs partitioned by BD genetics-associated gCREs. (6) Drug repurposing analysis based on the enrichment of BD dysregulated genes in differential gene expression profiles in response to pharmacological perturbations identified 428 drugs and compounds, including drugs targeting tumors, the nervous system, and the cardiovascular system, as potential treatment options for BD.

In summary, we present the largest blood histone modification profiling dataset in type I BD to date and comprehensive integration analyses that reveal the role of circulating immune components in BD based on genetic signals, epigenomic marks, and gene expression. We also suggest drugs and targets based on BD-associated immune dysfunction. Our study demonstrates the important role of white blood cells, a readily accessible biospecimen, in understanding BD pathogenesis and patient heterogeneity.

## Results

### An epigenomic atlas of 439k blood CREs across 180 individuals

To capture epigenomic differences in type I BD (referred to as BD henceforth) patients compared to control individuals, we profiled the genome-wide distribution of five histone marks using chromatin immunoprecipitation followed by high-throughput sequencing (ChIP-seq) in blood buffy coat samples from 180 participants including 88 patients and 92 controls from a mainly non-Hispanic white patient population seen at Mayo Clinic in Rochester, Minnesota (Fig. 1a, Supplementary Table 1, Supplementary Data 1). These five widely studied marks relevant to gene-regulatory processes include H3K27ac, which is associated with active promoters and enhancers, H3K4me1, a marker of enhancer regions, H3K4me3, a marker of promoters, H3K36me3, a mark that reflects transcriptional elongation, and H3K27me3, which is associated with Polycomb-repressed regions^24^.

Across all subjects, we identified 157k *cis*-regulatory elements (CREs) based on peaks in the epigenomic profiles of H3K27ac, 211k CREs for H3K4me1, 58k for H3K4me3, 161k for H3K36me3, and 248k CREs for H3K27me3 (Fig. 1a, Supplementary Data 2), covering 439k non-overlapping regions in the genome. Distribution analysis of CREs around genes showed that 76–88% of CREs across the five histone marks were located within 50 kb of the transcription start sites (TSS) or transcription termination sites (TTS) of a gene (Supplementary Fig. 1a). Consistent with being a mark of transcriptional elongation^25^, H3K36me3 CREs showed much higher average signals within gene bodies compared to promoter regions than CREs associated with other histone marks (0.81 vs. 0.18–0.35). The observed overlaps among different types of CREs were also consistent with their functions: we found 82% of H3K27ac CREs (active enhancers and promoters) overlapping with H3K4me1 CREs (active or poised enhancers), 84% of H3K4me3 CREs (active and poised promoters) overlapping with H3K27ac CREs, but only 3% of H3K27me3 CREs (repressed genomic regions) overlapping with H3K36me CREs (transcribed regions) (Supplementary Fig. 1b).

As CREs are frequently shared between tissues, we next sought to understand the activity profiles of these blood CREs in a broad collection of tissues. Specifically, we quantified the activity of 169k of our blood CREs annotated by the enhancer/promoter-associated marks H3K27ac, H3K4me1, and H3K4me3 based on the H3K27ac signal across 242 reference epigenomes from the Encyclopedia of DNA Elements (ENCODE), the National Institutes of Health Roadmap Epigenomics Mapping Consortium database, and the National Human Genome Research Institute’s Genomics of Gene Regulation (GGR) project^26–28^ and integrated in EpiMap^29^. We then binarized CRE activities based on these profiles across tissues and clustered them into 84 modules and 13 groups (G1-G13, Fig. 1b) using the k-centroids algorithm with the Jaccard distance as in the EpiMap^29^ (*Methods*). We found these blood CREs to be broadly active (G1) or active in multiple tissues (G2), with the rest being active predominantly in immune/blood cells (G3), epithelial/epidermal cells (G4), the placenta (G5), digestive tissues (G6), epithelial cancer (G7), miscellaneous organs (G8), fibroblast/aorta (G9), muscle (G10), spleen/adrenal (G11), brain (G12) and embryonic stem cells (G13).

We then investigated the enrichment of transcription factor (TF) binding motifs and gene ontology (GO) terms for each CRE module. The CREs broadly active or active in multiple types of tissues (G1 and G2) were more likely to appear in promoter regions (40% vs. 12% in the background), while those active only in certain tissue types were more likely to be from intergenic regions (31% vs. 26% in the background) (Fig. 1b), consistent with the previous findings that tissue specificity is mostly driven by intergenic enhancers^26^. By TF motif and GO enrichment analysis, we found immune-related TFs, such as IRF4^30^, SPI1^31^, and IRF5^32^, and immune response-related biological processes enriched in G3 (Supplementary Fig. 1c-e), confirming the functions of CREs primarily active in blood or immune cells. Interestingly, we also found CREs that were predominantly active in the brain (G12). This group was also enriched in two neuron-related TFs, RFX2^33^ and USF2^34^, and neuron differentiation-related GO terms, indicating potential sharing between blood and brain. Additionally, CREs broadly active across tissues (G1) were enriched for terms such as mRNA metabolic processes, multi-tissue CREs (G2) for negative regulation of apoptotic processes, and placenta-predominant CREs (G5) for cell migration and developmental regulation. Together, these results highlighted that CREs detected in blood may originate from distinct regulatory programs active in diverse tissues.

### Differential activity of CREs in bipolar disorder

Due to high inter-individual variability in peak detection and the lack of statistical robustness in defining BD-specific CREs (Supplementary Fig. 2), we then focused on whether the activity of CREs in blood is different in BD patients compared to controls. Since the buffy coat samples are mixtures of different immune cell types, the variation of CRE activity may also be impacted by cell fraction variation. We therefore estimated for each patient the cell fractions of six major white blood cell types, including CD4^+^ T cells, CD8^+^ T cells, B cells, natural killer cells, monocytes, and neutrophils, with our in-house deconvolution approach based on cell-type-specific CRE activity from reference epigenomes (*Methods*). Neutrophils showed the largest fraction, and these estimated fractions varied across individuals (Supplementary Fig. 3a). We next identified differential CREs (dCREs) between BD patients and controls for each histone mark. We applied surrogate variable analysis followed by a linear regression model to account for the hidden variables (*Methods*). As expected, inferred surrogate variables from different histone modifications were strongly correlated with the estimated cell fractions (see H3K27ac as an example in Supplementary Fig. 3b), suggesting that the impact of cell fraction variations was captured and thus corrected during dCRE analysis.

**Fig. 2:**
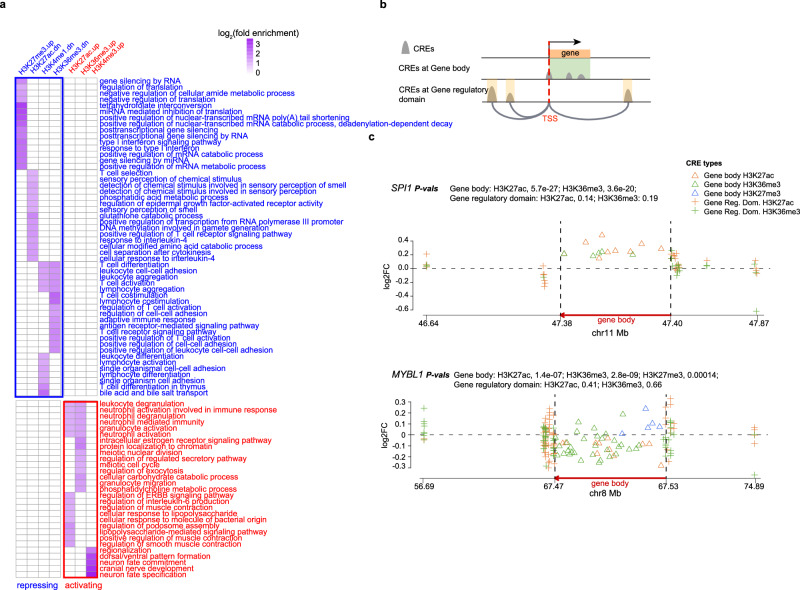
dCREs in BD and genes with BD-differential epigenomic signatures. **a** Enrichment of GO Biological processes in BD dCRE sets. The heatmap shows the log_2_ fold enrichment of GO terms (by row) for BD dCRE sets (by column) for each histone mark with different signs, where up-regulation of repressive marks and down-regulation of activating marks are grouped as “repressing” in blue and down-regulation of repressive marks and up-regulation of activating marks are grouped as “activating” in red. Up, upregulated; Dn, downregulated. **b** Schematic diagram to show CREs in a gene body or in a gene regulatory domain. **c** Two examples of genes with BD-differential epigenomic signatures. *SPI1*: consistently up-regulated dCREs in the gene body based on active marks (H3K36me3 and H3K27ac): *MYBL1*: consistently down-regulated dCREs based on active marks (H3K36me3 and H3K27ac) and up-regulated dCREs based on repressive marks (H3K27me3) in the gene body (scaled to be ⅓ of the x-axis, in red arrow). log2 (fold changes) of dCREs are shown in the y-axis. Nominal *P* based on two-sided meta analysis on differential signals of CREs of the same histone mark were shown. Source data are provided as a Source Data file.

In total, we detected 22k (H3K27ac), 16.1k (H3K36me3), 1.6k (H3K27me3), 805 (H3K4me1), and 1.6k (H3K4me3) dCREs (adjusted *P* 0.05), showing the largest number of overlapped dCREs between the active marks, such as the up-regulated dCREs shared between H3K36me3 and H3K27ac (*N* = 1638), and the down-regulated dCREs shared between H3K4me3 and H3K27ac (*N* = 576) (Supplementary Figs. 4a–c, 5a, Supplementary Data 3). GO enrichment analysis by GREAT^35^ also confirmed coordinated impacts of BD on CREs across different marks. The repressive regulation in BD (up-regulated repressive marks and down-regulated active marks) was enriched for gene silencing and adaptive immune response, while the active regulation in BD (up-regulated active marks and down-regulated repressive marks) was enriched for neutrophil activation (Fig. 2a). These results suggest an elevated level of inflammation and reduced adaptive immune response in BD^36^ and highlight the utility of epigenomic analysis for capturing altered physiological states in blood. Interestingly, several neuron-related pathways were also affected in peripheral immune cells in BD, including detection of chemical stimulus involved in sensory perception of smell and neuron fate commitment. This suggests shared molecular machinery between immune cells and neurons in BD.

We next investigated TFs that may underlie these epigenomic alterations in BD by using the motif enrichment analysis software Homer^37^. We identified 19 TF families showing motif enrichment in any dCRE set. Of the representative TFs, 15 were associated with BD or other psychiatric disorders based on genetics, differential signals, or mechanistic evidence^7,38–53^ (Supplementary Data S3). Six of these TFs (IRF8, SPI1, CEBPB, RORA, ATF1, and EGR3) are directly related to inflammatory response, while five TFs (SPI1, MECP2, MBD2, JDP2, and CTCF) function as chromatin regulators/remodelers. 18 of these dCRE-associated TFs also formed a protein-protein interaction network based on GeneMANIA^54^, indicating potential regulatory mechanisms connecting inflammatory responses to chromatin remodeling in the peripheral immune system of BD patients (Supplementary Fig. 5b).

**Fig. 3:**
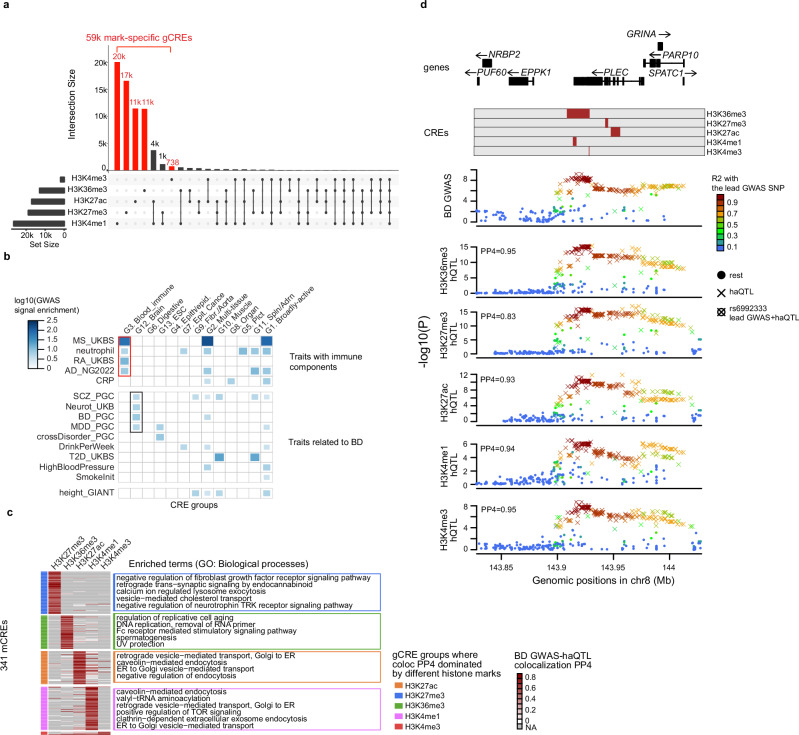
Genetics of CREs in blood and implications in BD. **a** Overlap among different gCRE sets of the five histone marks. The upset plot shows the number (y-axis) of CREs with various combinations of histone marks, with mark-specific CREs highlighted in red. **b** Genome-wide association study (GWAS) signal enrichments in CRE groups. The heatmap shows the log_10_ enrichment of different GWAS signals with LD score regression (by row, with groups labeled on the left) in different groups of CREs (by column) defined in Fig. 1b; red box, enrichment of immune components; black boxes, enrichment of brain component; enrichment was only shown for those with adjusted *P* < 0.25 (BH multiple test correction). MS multiple sclerosis, RA rheumatoid arthritis, AD Alzheimer’s disease, Neurot neuroticism, T2D type II diabetes, SmokeInit Smoking initiation, UKB UK biobank, UKBS UK biobank self-report, PGC Psychiatric Genomic Consortium, GIANT Genetic Investigation of ANthropometric Traits Consortium. **c** gCREs colocalized with BD GWAS. The heatmap shows the BD GWAS–hQTL colocalization signals for each merged CRE (mCREs) across various marks in different colors, with enriched biological processes annotated on the right. **d** BD–hQTL-colocalization at the *PLEC-GRINA* locus shown as an example of the colocalization of BD GWAS signals and hQTL signals across different marks with the genomic positions of genes and CREs annotated on top. Source data are provided as a Source Data file.

We next inferred differential signals at the gene level. We generated multiple differential scores by meta-analysis of differential signals of CREs from each histone mark using two CRE–gene mapping strategies as illustrated in Fig. 2b (*Methods*): (1) CREs located within the gene body and (2) CREs located within the gene regulatory domain defined as the genomic regions linked to the promoter of the gene based on promoter capture Hi-C data in immune cells. H3K36me3 at the gene body is an epigenomic mark for transcription elongation^55^ and is thus a reasonable surrogate for transcriptional activity. We found that two scores from the gene body (H3K27ac and H3K27me3) and two scores from the gene regulatory domains (H3K27ac and H3K36me3) were strongly correlated with the score for gene body H3K36me3 (Supplementary Fig. 5c, d). Our findings are also consistent with the known association between these three marks (H3K27ac, H3K36me3, and H3K27me3) and the level of gene transcription^56^. We then aggregated these five strongly correlated scores and inferred the genes with BD down-regulated (*N* = 631) and up-regulated (*N* = 2001) epigenomic signatures (Supplementary Fig. 5e, Supplementary Data 3, *Methods*). GO enrichment analysis of these genes with Enrichr showed downregulated adaptive immune response and up-regulated innate immune response (Supplementary Data S3), consistent with the results for BD dCREs above (Fig. 2a). The genes with BD-differential epi-signatures included six of the TFs we earlier identified as potential regulators for the dCREs, including SPI1, FLI1, MYBL1, MBD2, JDP2, and CEBPB (*SPI1* and *MYBL1* shown as examples in Fig.3c), genes associated with neutrophil extracellular traps (*TLR4* and *PADI4*)^57^, and differentially expressed genes from previous BD blood studies^58,59^ that are involved in DNA damage (*OGG1*) and the TNF-α signaling pathway (*TNFAIP6*, *TNFRSF10C*, and *TNFAIP2*).

### Genetic regulation of CRE activity and implication of CRE activity in BD genetic associations

To dissect BD-associated genetic variants, we next sought to identify the impacts of genetic variants on CRE activity by mapping histone quantitative trait loci (hQTLs), i.e., specific SNPs significantly associated with histone modification levels, at CREs across individuals. These CREs are referred to as genetically influenced CREs (gCREs). We followed the GTEx eQTL mapping protocol as follows^60^: (1) we identified latent factors inferred by probabilistic estimation of expression residuals (referred to later as PEER factors)^61^, (2) included top PEER factors as covariates together with age and sex during hQTL mapping, and (3) corrected for multiple testing based on permutation. We also verified that the cell fraction variation was corrected for during QTL mapping by confirming that the top PEER factors were strongly correlated with the cell fractions estimated above (Supplementary Fig. 6a). We identified 1.5 M H3K27ac hQTLs (targeting 17k gCREs), 2.2 M H3K4me1 hQTLs (27k gCREs), 200k H3K4me3 hQTLs (1.8k gCREs), 1.2 M H3K36me3 hQTLs (13k gCREs), and 1.4 M H3K27me3 hQTLs (19k gCREs) (Supplementary Fig. 6b, Supplementary Data 4, *Methods*). As expected, a major proportion of the lead hQTLs (40-70% across the five histone marks) were within 20 kb of the center of the target peaks, showing significant enrichment compared to the background (Kolmogorov–Smirnov test *P* = 2.2 × 10^−16^) (H3K27ac shown as an example, Supplementary Fig. 6c).

Given the substantial overlap among CREs labeled by different histone marks (Supplementary Fig. 1b), we next investigated the likelihood of their shared genetic regulation. We found that the majority of gCREs were specific to individual marks, 59k in total across the genome, demonstrating the value of our multi-mark approach to capture genetically influenced regulatory elements (Fig. 3a). We also identified 4k genomic regions with overlapping gCREs from H3K27ac and H3K4me1, and 1k regions with overlapping gCREs from H3K27me3 and H3K4me1 (Fig. 3a). Following our previous studies on QTL sharing across tissues based on directional consistency of lead QTL effect size^62^, we revealed pairs of histone marks with higher degree of hQTL sharing (e.g., among H3K27ac, H3K4me1, and H3K4me3), consistent with their active regulatory roles, and others with low sharing levels (e.g., between H3K36me3 vs. H3K4me1, and H3K4me3 vs. H3K27me3), indicating complex genetic regulations across different histone marks (Supplementary Fig. 6d).

Based on the different annotations of CRE groups from blood (CRE groups across various primary tissues/cell types in Fig. 1b, blood dCREs in BD in Fig. 2a, and gCREs in Fig. 3a), we next asked whether these annotations could shed light on the contexts of genetic variants either associated with complex diseases/traits with known immune components or related to BD. We carried out stratified LD score regression (LDSC) analysis^63^ to quantify the enrichment of GWAS signals overlapping with different CRE annotations. Immune-related traits, including multiple sclerosis (MS), rheumatoid arthritis (RA), Alzheimer’s disease (AD) and neutrophil counts, showed strong enrichment of GWAS signals in CREs predominantly active in blood/immune system (G3) and in gCREs (H3K27ac or H3K4me1), confirming the strong immune components in these traits (red boxes in Fig. 3b and Supplementary Fig. 6e). Interestingly, these diseases also showed significant enrichment of GWAS signals in BD dCREs (H3K27ac, H3K36me3, and H3K4me1), indicating they might share immune components with BD. BD, together with SCZ, neuroticism, and MDD, showed significant GWAS signal enrichment in CREs detected in blood and predominantly active in the brain or in neurons (G12) (black boxes in Fig. 3b), indicating that CREs in the blood may mirror similar epigenetic regulation in the brain, and capture the impact of these disease-associated genetic variants.

To identify specific gCREs that share genetic drivers with BD and thus play important roles in BD etiology, we performed BD GWAS–hQTL colocalization analysis by the R package coloc^64^ (*Methods*). We identified 341 merged BD-associated gCREs from different marks in our dataset (posterior probability, coloc PP4 > 0.5), with the majority being histone-mark-specific gCREs (Fig. 3c). Interestingly, enrichment analysis by rGREAT revealed that genes near these gCREs had convergent functions related to the endoplasmic reticulum (ER), indicating that ER homeostasis in circulating immune cells may contribute to BD etiology^65,66^ (Fig. 3c). In total, we identified 97 BD GWAS loci (*P* < = 1 × 10^−6^) potentially regulating blood gCREs of any mark based on GWAS–hQTL colocalization (Supplementary Data 4). We found that gCREs of a single mark could at most explain 60% of these loci (H3K4me1, *N* = 58), demonstrating the necessity of our multi-mark approach to comprehensively dissect the GWAS signals. We also found that 61% of these loci were associated with gCREs from multiple histone marks. For example, at the *PLEC*-*GRINA* locus, which has been linked to ER homeostasis^67,68^, hQTL signals across all five histone marks showed strong colocalization with the BD GWAS signal (posterior probability, coloc PP4 > 0.8) (Fig. 3d), indicating a consistent impact of BD-associated genetic variants over the histone marks. Notably, *GRINA* was previously identified as a differentially expressed gene in the brain samples of individuals with major depressive disorder^69^, a disorder closely related to BD.

In light of the BD GWAS signal enrichment in the CREs primarily active in the brain (G12. Brain, Fig. 3b) and the BD GWAS–hQTL colocalization events in the blood (Fig. 3c), we next asked whether the GWAS–hQTL colocalizations observed in the blood might mirror colocalizations in the brain. Such a scenario could be supported by enrichment of BD GWAS–hQTL colocalization events for the gCREs shared between the blood and the brain. We first found that of the blood H3K27ac gCREs (*N* = 30.0k), those shared with the brain (*N* = 12.6k; based on our previous eGTEx study^70^) also significantly enriched gCREs near BD GWAS signals (within 100 kb, *N* = 982, one-sided Fisher’s exact test, *P* = 0.0032). However, when we considered the subsets of the blood H3K27ac gCREs near BD GWAS signals (*N* = 982), the gCREs shared in the brain (*N* = 456) did not enrich the gCREs showing BD GWAS–hQTL colocalization (coloc PP4 > 0.5, *N* = 103, one-sided Fisher’s exact test, *P* = 0.29), and neither did the gCREs sharing hQTL regulation between the blood and the brain (brain shared hQTL *P* < = 0.01, *N* = 31, shared hQTLs defined in Supplementary Fig. 6f and *Methods*, one-sided Fisher’s exact test, *P* = 0.46). Together, our results suggest that the BD GWAS variants we captured from hQTLs in the blood may specifically impact CRE function in the circulating immune cells. Alternatively, these BD GWAS variants may function in immune cells both in the blood and the brain but were only captured by blood hQTLs because of a larger power due to both a larger sample size and a much larger number of immune cells in the blood than in the brain. Either explanation indicates a potentially causal role of the circulating immune system in BD etiology.

### BD driver genes and blood-brain sharing

We next sought to prioritize potential BD driver genes in blood. We reasoned that the driver genes should both be driven by the BD GWAS signals and show BD-differential signals at their gene regulatory domains, i.e., CREs linked to their promoters. We first explored the global relationship between BD GWAS signals and BD differential signals at the level of chromatin interaction blocks, namely, clusters of chromatin regions connected by links within 1 Mb based on promoter capture Hi-C data in immune cell types from BLUEPRINT^71^. For each of the 1370 chromatin interaction blocks identified, we quantified the enrichment of the dCREs for each histone mark over the background, and the density of the links connecting the chromatin regions. Our results showed positive correlation between link density and dCRE enrichment for the blocks with dCREs across all five histone marks (Supplementary Fig. 7a), indicating that the dCRE signals may impact each other through these chromatin interactions inside each block. Together with the gCREs associated with BD genetic signals based on colocalization or Mendelian randomization, analysis at the level of chromatin interaction blocks revealed that the blocks annotated with BD GWAS signals were more likely to show enrichment of dCRE signals, except H3K27me3-downregulated dCREs and H3K4me3-upregulated dCREs (Supplementary Fig. 7b). These results suggest that BD GWAS signals at these chromatin interaction blocks contributed to blood epigenomic dysregulation in BD, supporting the notion that driver genes for BD can be identified by analyzing blood samples.

**Fig. 4:**
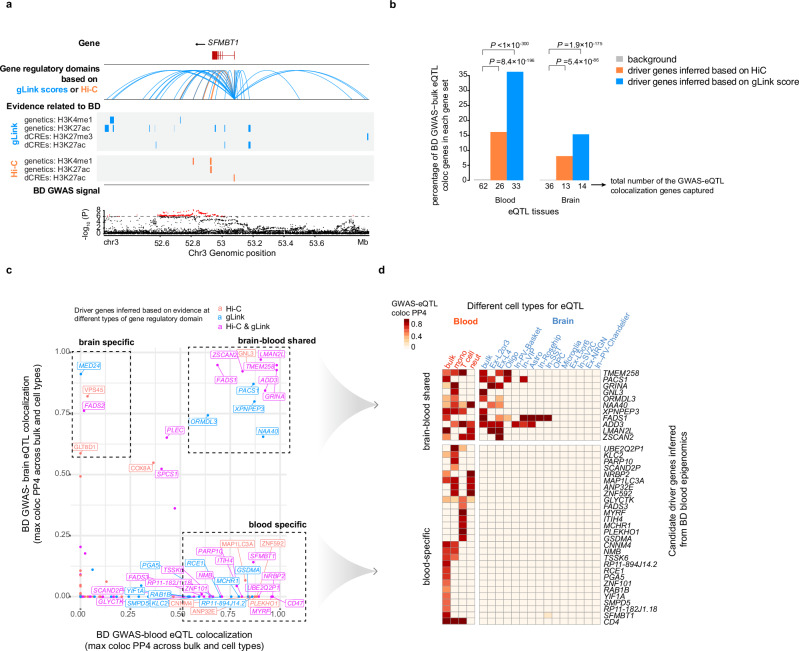
BD Driver genes shared between the blood and brain. **a** Driver genes inferred based on genetic signals and differential signals at gene regulatory domains (GRD). *SFMBT1*, which was identified as a driver gene in blood for BD based on its links (based on either gLink in blue or Hi-C in orange) to both CREs genetically associated with BD (e.g., genetics: H3K4me1) and BD dCREs (e.g., Dn_H3K27ac), shown as an example. The BD GWAS nominal *P* (two-sided association test) is shown at the bottom panel. **b** Enrichment of driver genes in BD GWAS–eQTL colocalization genes. The bar plot compares the percentage of BD GWAS–eQTL colocalized genes from the brain or blood in three different gene sets: all eGenes as background (in gray), driver genes based on Hi-C linking (in orange), and driver genes based on gLink scores (in blue). Asterisks indicate the significance of the tests comparing different proportions (one-sided proportion test). **c** Driver genes supported by BD GWAS–eQTL colocalization from blood or brain. The scatter plot shows the BD GWAS–eQTL colocalization signal (max coloc PP4) across brain tissue/cell types (y-axis) and blood tissue/cell types (x-axis), with tissue-specific and shared driver genes highlighted and genes supported by different types of linking evidence in different colors. **d** BD GWAS–eQTL colocalization based on eQTL at the level of cell types. The heatmap shows the BD GWAS–eQTL colocalization signal (coloc PP4) for driver genes (by row) with strong GWAS–eQTL colocalization signal in the blood (red) or in brain (blue) (coloc PP4 ≥ 0.5) across different cell types or bulk tissues (by column). Genes were grouped based on whether there were also strong colocalization signals in any of the brain bulk tissue or cell types. Source data are provided as a Source Data file.

To identify driver genes based on BD-associated gene regulatory domains, we leveraged two types of CRE–gene linking evidence: (1) physical evidence, which is based on promoter capture Hi-C data in immune cells^71^, and (2) genetic evidence, namely, gLink scores, which we defined in our eGTEx study^70^. We assessed the consistency between the two pieces of evidence and confirmed that gLink scores outperformed background and the permuted scores (controlling for CRE–gene distances) in predicting Hi-C linkages (Supplementary Fig. 7c, *Methods*). We then prioritized driver genes as those linked to both upstream BD genetic signals—specifically gCREs associated with BD genetics either by GWAS–QTL colocalization or Mendelian randomization—and downstream epigenomic signals, namely BD dCREs. Using this method, we identified 161 and 91 BD driver genes in blood via CRE—gene linking based on Hi-C and gLink scores (overlap *N* = 37), respectively. Consistent with the result at the level of chromatin interaction blocks, genes linked to dCREs showed enrichment in genes with BD GWAS-associated regulatory elements from the two linking strategies (*P* = 1.3 × 10^−38^ and 9.7 × 10^−10^ for Hi-C and gLink scores, respectively). As an example, we showed results for *SFMBT1*, a gene encoding for a histone-binding co-repressor protein, which has previously been associated with BD^72^ (Fig. 4a).

We next tested whether eQTL signals of BD driver genes from either the blood or brain support their association with BD genetic signals. To increase the power of GWAS–eQTL colocalization, we also included eQTL data at the cell type level for blood from the BLUEPRINT project^73^ and for the brain from a ROSMAP cohort^74^. Colocalization analysis for the driver genes resulted in 36 and 63 genes based on brain and blood eQTLs (coloc PP4 ≥ 0.5), respectively. We then used Enrichr^75^ to identify enriched GO terms for both gene sets. Notably, only blood-eQTL-based candidate genes enriched terms such as Golgi vesicle-mediated transport (*P* = 0.005) and regulation of Fc receptor mediated stimulatory signaling pathway (*P* = 0.015), closely mirroring our findings based on blood hQTL-colocalized gCREs (Fig. 3c), suggesting a convergence between BD transcriptomic and epigenomic signals. The driver genes identified via both types of linking evidence showed significant enrichment (one-sided proportion test *P* < 1 × 10^−85^) for the candidate genes based on GWAS–eQTL colocalization in blood or brain samples (coloc PP4 ≥ 0.5) (Fig. 4b). The higher enrichment in driver genes based on gLink scores vs. Hi-C data in both tissues also confirmed that the CRE–gene links identified via gLink scores could prioritize genes with stronger disease-associated genetic signals^70^. We identified 46 driver genes showing GWAS–eQTL colocalization signals in either the blood or brain (coloc PP4 ≥ 0.5), including 28 genes with colocalization only in the blood (blood coloc PP4 ≥ 0.5, brain coloc PP4 < 0.25), 4 with colocalization only in the brain (brain coloc PP4 ≥ 0.5, blood coloc PP4 < 0.25), and 11 genes with colocalization in both tissues (Fig. 4c, Supplementary Data 5). These results indicate that most BD genetic signals captured in our blood samples based on GWAS–eQTL colocalization may specifically function through the circulating immune system rather than in the brain.

We next focused on 39 driver genes supported by GWAS–eQTL colocalization in the blood (*colo*c PP4 ≥ 0.5) and examined them further at the cell type level (Fig. 4d, Supplementary Data 5). While most genes were specifically supported by blood eQTL signals, colocalization at the cell-type level also revealed a strong pattern of nine driver genes shared between immune cells and excitatory pyramidal cells of neocortical layers 2/3 (Ex-L2or3) or layer 4 (Ex-L4) in the brain, instead of microglia, the primary brain immune cell type. Interestingly, these neuronal cell types may be causal in BD since they have previously been identified as involved in mediating stress-induced depressive behaviors^76,77^. It indicates these nine genes captured by cell-type-specific eQTL signals from both neuronal and immune cell types may drive BD pathogenesis primarily in neuronal cell types while mirroring genetic effects in immune cells. Our analysis also uncovered genes such as *ORMDL3* and *MAP1LC3A*, which are related to ER stress or calcium signaling (Fig. 4d). These pathways are enriched around CREs that potentially share genetic drivers with BD (Fig. 3d), and have also been linked to BD^65,78,79^. Furthermore, among the TFs enriched for dCREs (Fig. 2b), ATF1, CEBPB, NFIA, and NRF1 are also related to ER stress^80–83^. Together, these results suggest that BD genetic signals may increase the risk of dysregulated ER homeostasis and calcium signaling in the circulating immune system, potentially contributing to BD pathogenesis^9^.

### Heterogeneity of CRE activities in BD patients reveals phenotypic subtypes

Due to the various risk factors for BD, such as genetics, early exposure to trauma or infection, and comorbidity patterns^84^, BD pathogenesis is believed to be highly heterogeneous^4,85^. To determine whether epigenomic profiles in the blood could reflect such heterogeneity, we first filtered dCREs from each histone mark for robust signals based on dCREs enrichment analysis in chromatin interaction blocks as described above (*Methods*). We then carried out latent factor analysis by MOFA^86^ to capture the variation of the selected dCREs in our cohort (Supplementary Fig. 8a) and identified 24 latent factors (LFs) across different histone marks (Supplementary Fig. 8b). Next, we investigated whether these latent factors, representing major epigenomic variation across individuals, were driven by any physiological or medical information from the EHR or with the estimated cell fraction variation across all BD patients. Notably, LF2-5 showed a strong correlation with the estimated cell fractions; LF7 was significantly correlated with terms related to infections of a specific organ; LF10 with inflammation, infection, diabetic, and cardiometabolic status-related terms; LF11 with NK cell fractions and cytomegalovirus (CMV) infection, which has been reported to be associated with BD^87^; LF20 was correlated with digestive system-related medicine records; and LF8 and LF15 were correlated with lithium total score, reflecting cumulative lithium exposure in BD treatment (adjusted *P* < 0.05, Supplementary Fig. 8c). These correlations suggest that LFs reflect various sources of epigenomic variation across BD patients.

**Fig. 5:**
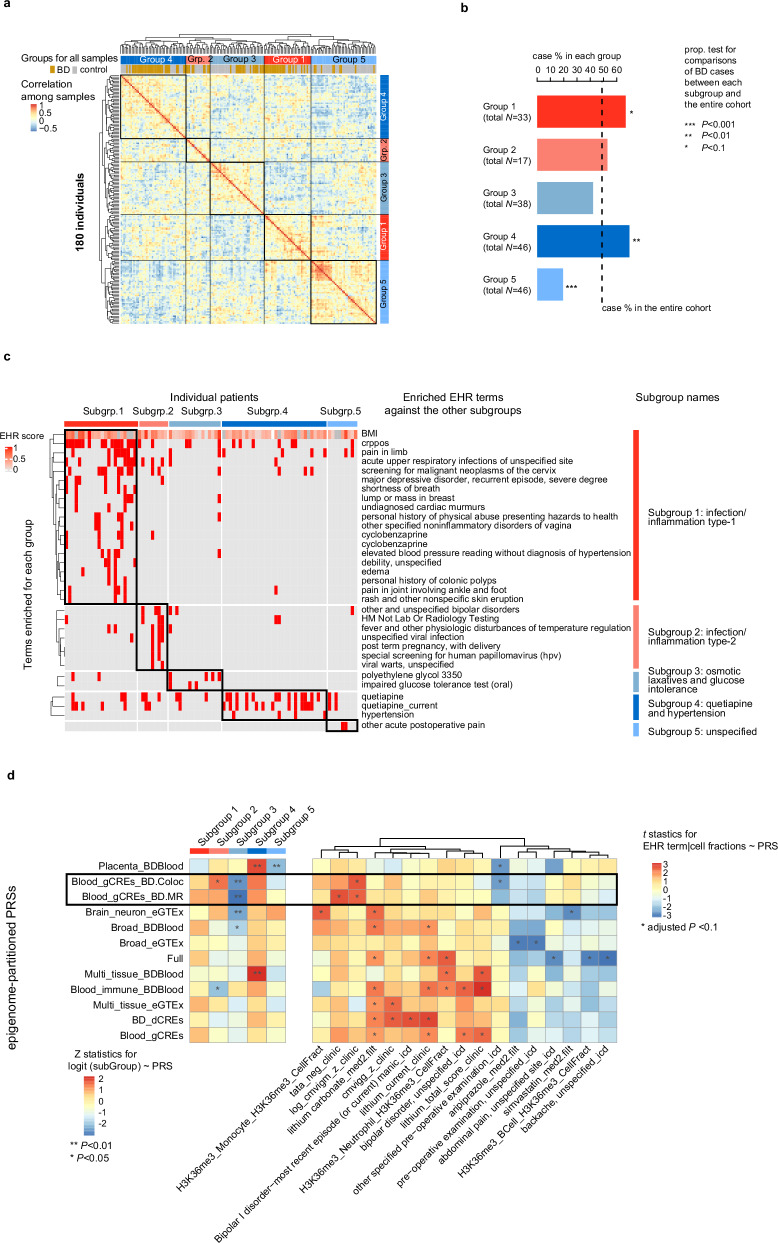
Subtyping of BD patients and annotation of subgroups. **a** Sample clustering based on correlation across latent factors. The heatmap shows the correlations (Pearson’s correlation coefficient) among patients and controls (by row and by column) based on the latent factors and hierarchical clustering based on the correlation matrix. **b** Different proportions of BD in each group. Order the same as in panel a. Nominal *P*s (the two-sided proportion test) to compare BD fractions between each group and all samples are 0.06, 0.93, 0.50, 7.9 × 10^−3^, and 1.2 × 10^−4^ for groups 1 to 5, respectively. **c** Patient groups capture features from EHR. Top: the heatmap shows the value of latent factors (by row) across individuals (by column) for each patient group; bottom: the heatmap shows the normalized EHR score (1 for presence, 0 for absence for categorical terms, continuous terms are normalized to the range of 0-1) for each individual (by column) for each term (by row) with the group name on the right. **d** Epi-partitioned PRSs could explain BD subtypes and EHR terms across BD patients. The left heatmap shows the *Z* statistics from binomial regression models that predict the BD subtypes (binary, by column) across individuals based on various epi-partitioned PRSs (by row) correcting for sex, age, and appearance of EHR terms related to infection/inflammation, glucose, hypertension, pain, and abuse. Asterisks indicate the significance levels of the tests, where nominal *P*s are from two-sided Wald Test. The right heatmap shows the *t* statistics of linear models that predict known factors (by row) across individuals based on various epi-partitioned PRSs (by column) correcting for sex and age. Asterisks indicate adjusted *P* < 0.1 based on two-sided *t*-test followed by BH multiple test correction. Placenta_BDBlood, Broad_BDBlood, Multi_tissue_BDBlood, and Blood_immune_BDBlood are PRSs built based on CRE modules, Placenta (G5), Broadly-active (G1), Multi-tissue (G2), and Blood/immune (G3) derived in this blood study, respectively; Blood_gCREs_BD.Coloc, Blood_gCREs_BD.MR, Blood_gCREs, and BD_dCREs are PRSs built based on gCREs associated with BD GWAS based on colocalizaiton or Mendelian Randomization, all gCRE set, and dCRE set defined in this study, respectively; Brain_neuron_eGTEx, Broad_eGTEx, and Multi_tissue_eGTEx are PRSs built based on CRE modules derived in the previous eGTEx report. Source data are provided as a Source Data file.

We then clustered all patients and controls with these LFs based on the correlation matrix among them (Fig. 5a, Supplementary Fig. 8d, *Methods*). There were five groups of individuals showing significantly different proportions of the patients (Chi-squared test *P* = 9.1 × 10^−6^). Groups 1 and 2 showed higher patient proportions while group 5 showed a lower proportion than the entire cohort (two-sided proportion test), indicating that different BD risks or comorbidities were associated with the different groups (Fig. 5b). We next investigated whether patients from each group, namely patient subgroups, reflected heterogeneity related to BD pathology or physiology. We annotated each patient subgroup with EHR data to identify differentially enriched terms vs. other groups (Fig. 5c). Patients in subgroups 1 and 2 were both enriched for infection- and inflammation-related terms, consistent with the enriched terms for dCREs in BD (Fig. 2a). Subgroup 3 showed enrichment of the terms polyethylene glycol 3350, which indicates osmotic laxative usage, and impaired glucose tolerance test (oral). Both terms relate to BD: Osmotic laxatives are commonly used to treat constipation, which is one of the major side effects of antipsychotic medicines^88^ and diabetes has been reported to be associated with BD^89^. The prevalence of BD is also significantly higher in irritable bowel syndrome patients, who may present with constipation, than controls^90^. Subgroup 4 showed enrichment of the term quetiapine_current, an atypical antipsychotic medicine for BD, and hypertension, which BD patients have a higher risk for ref. ^91^. We did not identify strong BD-relevant terms for subgroup 5, which also showed the lowest fraction of BD patients compared to other subgroups (Fig. 5b). Importantly, we observed similar EHR enrichment patterns when comparing each subgroup to controls, including leukocytosis (*P* = 0.01) and joint pain (*P* = 0.02) for Group 1, constipation (*P* = 0.04) and impaired glucose tolerance test (*P* = 0.02) for Group 3, and hypertension (0.01) for Group 4, further confirming that the latent factor-based clusters capture meaningful clinical heterogeneity.

To confirm that different patient subgroups show distinct blood epigenomic signatures, we also identified CRE biomarkers across five histone marks for each patient subgroup. The GO annotations of the 12-2200 CRE biomarkers identified for each subgroup revealed biological processes consistent with the EHR terms characterizing individual subgroups (Supplementary Fig. 8e). For example, the biomarker CREs (H3K36me3 and H3K4me1) for subgroups 1 and 2 showed enrichment for infection- and inflammation-related terms; the biomarker CREs (H3K27ac) for subgroup 3 showed enrichment for inositol biosynthetic processes and response to lipopolysaccharide, which are both related to diabetes^92,93^; and the biomarker CREs (H3K27ac) for subgroup 4 showed enrichment for cellular responses to thyroid hormone stimulus, which is related to hypertension^94^.

We next explored the genetic basis of these patient subgroups by analyzing the contribution of aggregated genetic signals, specifically, the BD polygenic risk score (PRS; referred to as the “full” model henceforth). To facilitate capturing the genetic heterogeneity of this complex disease in finer detail, we also considered subsets of GWAS single-nucleotide polymorphisms (SNPs) based on epigenomic annotations in the PRS models in a manner similar to the computation of pathway-based PRSs^95^. Specifically, we partitioned the PRS by blood CRE groups primarily active in different tissues or cell types (Fig. 1b), dCREs (Fig. 2b), gCREs (Fig. 3a), and CRE sets detected in brain, muscle, heart, and lung samples from a previous eGTEx report^70^, generating “epi-partitioned PRSs” (*Methods*, Supplementary Fig. 9a, b, Supplementary Data 6). We found PRSs partitioned by CRE groups to be primarily active in the immune system and placenta, active across multiple tissues, and gCREs identified in our blood study showed significant differences between BD and control individuals (Supplementary Fig. 9c). Another two PRSs, which were built based on gCREs associated with BD genetics either by GWAS-QTL colocalization (blood_gAREs_BD.coloc) or Mendelian randomization (blood_gAREs_BD.MR), are particularly interesting. Though they did not show significant differences between patients and controls, however, these two scores could explain inter-individual variation among BD patients that the full model missed, suggesting that this inter-individual variation may reflect the BD genetic risk projected on the immune system (Fig. 5d). For example, these two PRSs distinguished subgroups 2 and 3 from the rest of the patients. Both of them were correlated with the reactivation of cytomegalovirus (term “CMV IgM”) but not with CMV infection (term “CMV IgG”), indicating a genetic component underlying the previously reported association between CMV reactivation and BD^87^. They were also correlated with the epigenomic latent factor LF9 across all patients (Supplementary Fig. 9d). Importantly, these pieces of evidence support that these two PRSs, reflecting the BD genetic signals associated with dysfunction in circulating immune cells, could capture epigenomic and phenotypic variations among patients. All these results suggest that the integration of profiles based on five epigenomic marks could capture the heterogeneity of the patients—which may arise from a complex interplay of genetic factors, medicine taken, and physiological conditions—providing a unique perspective for therapeutic intervention.

### BD drug response and repurposing

**Fig. 6:**
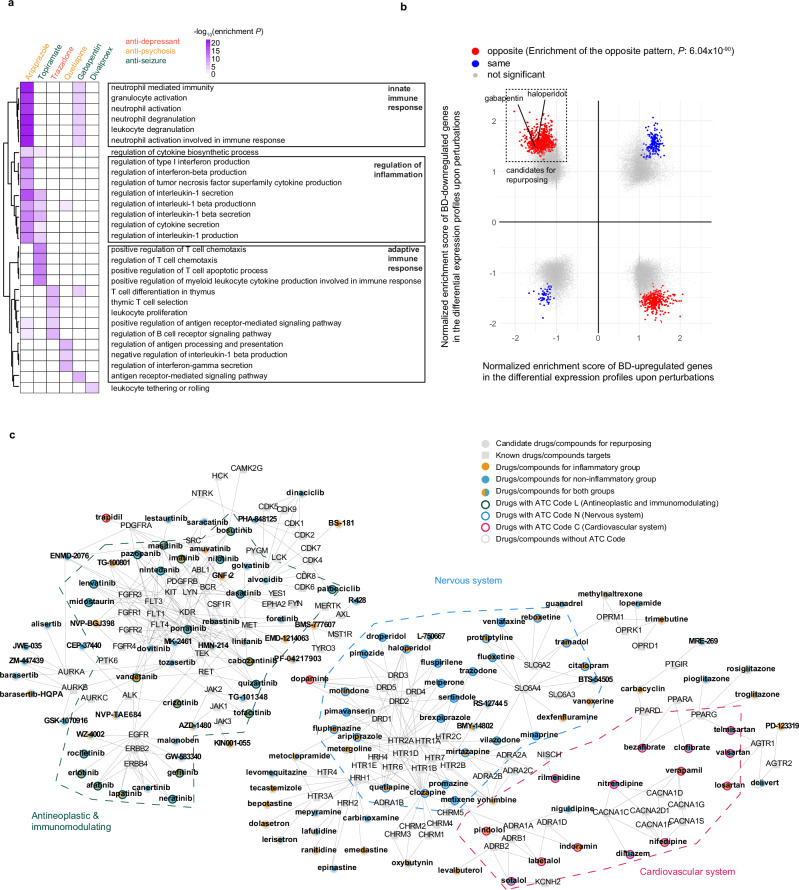
BD drug response and drugs repurposing. **a** Immune-related processes enriched in CREs responding to BD medicine. The heatmap shows the -log_10_ (enrichment *P*) of biological processes (by row) for each CRE set responding to BD drugs (by column), with different groups of processes highlighted on the left. Nominal *P*s (two-sided Fisher’s exact test) are shown. **b** Enrichment of genes with BD up/down-regulated epi-signatures in the expression profiles of hematopoietic cell lines exposed to pharmacological perturbations from CMap. Each dot represents an expression profile upon a perturbation; the x-axis and the y-axis represent normalized enrichment scores (NES) for the genes with BD up- and down-regulated epi-signatures, respectively. Colored dots indicate significant enrichment (nominal one-sided *P* < 0.001), with red dots representing differential expression profiles with *opposite* directions of NES for BD up- and down-regulated genes (significantly enriched, one-sided Fisher’s exact test *P* = 6.04 × 10^−90^), while blue dots representing differential expression profiles with the same directions of NES for the BD up- and down-regulated genes. **c** Candidate drugs for repurposing and their targets. Nodes represent either drugs (square) or targets (circle) with labels and edges connecting drugs to their targets. The network includes representative connected subgraphs, targeting common mechanisms between inflammatory and non-inflammatory BD subgroups, where at least one drug (in orange) is shared. These subgraphs show a high proportion of drugs originally designed for cancers, nervous system and cardiovascular disorders as labeled. ATC, Anatomical Therapeutic Chemical. Source data are provided as a Source Data file.

To reveal immune response to BD interventions, we then identified tens to thousands of CREs for each histone mark affected by medications in BD patients. GO enrichment analysis showed that the CREs affected by six of these drugs enriched genes from immune-related pathways (Fig. 6a, *Methods*). For example, the antipsychotic drug aripiprazole, a dopamine system stabilizer^96^, was strongly associated with innate immune response pathways and inflammation and, to a lesser extent, with adaptive immune response pathways. The anti-seizure drugs topiramate and gabapentin shared with aripiprazole their association with the innate immune response- and inflammation-related pathways, respectively. Quetiapine and trazodone were associated with adaptive immune responses, while divalproex was related to the chemotaxis of leukocytes. These results raise the possibility that these drugs, though designed to target neuronal signaling in the brain, may also affect immune functions either as part of their therapeutic effects or as side effects. We also found that these drugs may reverse the epigenomic impact associated with BD in a histone mark–dependent manner. For example, Gabapentin anti-correlated with BD effects on H3K27ac dCREs (*P* < 2.2 × 10^−308^) and BD-colocalizing gCREs (*P* = 4.9 × 10^−6^) (Supplementary Fig. 10a, b). Besides immune-related pathways, we also noticed pathways known to be affected by certain BD drugs, such as potassium ion transport associated with quetiapine^97^, and telomere and DNA repair with lithium^98,99^ (Supplementary Data 7). These results suggest that the peripheral immune system could provide insights into the physiological responses to, and potential mechanisms of action of, drugs primarily designed for central nervous system diseases.

We then carried out drug repurposing analysis to seek drugs and compounds that could reverse BD-associated immune dysfunctions as represented by the gene sets with up- or down-regulated epi-signatures defined above (Fig. 2c, Supplementary Data 3). We applied gene set enrichment analysis (GSEA)^100,101^ to assess the representation of these BD signature genes in the differential gene expression profiles of hematopoietic cell lines treated with 33,627 compounds in Connectivity Map (CMap)^102^ (*Methods*). Out of 49,488 differential expression profiles, 1232 showed significant normalized enrichment scores (NES, nominal *P* < 0.001) for both up- or down-regulated BD signature genes (colored spots in Fig. 6b). Further interrogation revealed that a significant majority of these profiles (1014 representing 82% of the total, one-sided Fisher’s exact test, *P* = 6 × 10^−90^) showed opposite patterns of BD gene enrichment: positive NES for BD-downregulated and negative NES for BD-upregulated genes (red spots in the upper left quadrant in Fig. 6b) or vice versa (red spots in the lower right quadrant in Fig. 6b), confirming an overall antagonistic relationship between up- and down-regulated BD genes across perturbation conditions. Importantly, 697 of these differential expression profiles, corresponding to 428 compounds targeting 279 proteins, showed an inverse pharmacological impact on BD signature genes, i.e., upregulation of the BD-downregulated and downregulation of the BD-upregulated genes (purple box in Fig. 6b, Supplementary Fig. 11a, Supplementary Data 5). These drugs/compounds are particularly noteworthy, since they represent potential candidate agents that could ameliorate BD-associated immune dysregulation.

Next, we tested whether the predicted responsiveness of BD patients to immune cell-targeting drugs and compounds could be further refined by considering BD patient groups established above. Based on the result above, we split patients into an inflammatory group (patient subgroups 1 and 2) and a non-inflammatory group (patient subgroups 3, 4 and 5) (Fig. 5c) and identified differential CREs across all five histone marks for each patient group compared to all the controls. We followed the same procedure to identify the genes with BD epi-signatures (Supplementary Data 7) and then candidate drugs that reversed these BD-associated changes. Similarly to the results from the entire BD patient cohort in Fig. 6b, BD genes with up- and down-regulated epi-signatures from both the inflammatory and non-inflammatory groups showed a similarly antagonistic pattern across CMap differential expression profiles (Supplementary Fig. 11b, c). We focused on drugs/compounds that reversed patient group-dependent BD signature genes in the differential expression profiles (purple boxes in Supplementary Fig. 11b, c) and linked them to their known targets in bipartite networks. Most drugs/compounds and target proteins were either shared by the two groups or connected to the shared ones in various subnetworks, indicating largely shared mechanisms between the two patient groups (Fig. 6c). We identified a subnetwork (Nervous system in Fig. 6c) with traditional BD drugs (e.g., aripiprazole) and BD drug targets (e.g., SLC6A4^103^), suggesting that the implicated drugs could reverse BD-associated immune dysfunction in immune cells as a potential mechanism of action. This subnetwork also contained a wide range of receptors and transporters for serotonergic, histaminergic, catecholaminergic, opioid, and cholinergic neurotransmission/signaling. Another subnetwork (Antineoplastic and immunomodulating in Fig. 6c) with multiple drugs primarily designed for cancers, including various inhibitors of tyrosine kinases FLT3, Aurora Kinases, JAK2, and EGFR, could also modulate immune dysfunctions as expected. We also noticed cardiovascular drugs such as nifedipine, bezafibrate, verapamil, and pindolol, either connected to the previously mentioned *Nervous system* subnetwork or from an independent subnetwork (Cardiovascular system in Fig. 6c), which have also been reported to show potential therapeutic effects in BD or related disorders^104–107^. In each group we also found unique drug–target pairs (Supplementary Fig. 11d, e). The list of drugs specific for the non-inflammatory group showed a higher proportion of cardiovascular drugs relative to the inflammatory group (Anatomical Therapeutic Chemical Code C, 7 of 24, i.e., 29% vs. 2 of 15, i.e., 13%) (Supplementary Table 2). Notably, diosmin, a cardiovascular drug that ameliorated depression behavior in an LPS-induced mouse model^108^, was unique to the non-inflammatory BD patients in our study. Together, these results further highlight the value of leveraging blood epigenomic marks of BD patients for facilitating mechanism-based patient classification.

## Discussion

By analyzing samples and information readily available from living patients (buffy coat cells, genotypes, and EHR data), our study provides unique insights into the immune dysregulation of type I BD and the patient heterogeneity in our cohort, thereby also offering a roadmap for similar investigations into other psychiatric disorders. We integrated multimodal data, including 833 ChIP-seq profiles across five major histone marks and genotypes from WGS data, as well as ICD-9 codes, procedure codes, patient-provided information, lab tests, and prescription codes from the EHR of 180 Mayo Biobank participants. We included BD patients and control participants matched to case subjects lacking any psychiatric condition or disorder based on age and sex. We specifically explored the potential role of circulating immune cells in BD, for which supportive data have been steadily accumulating^10,109,110^. Our analysis identified genes with strong genetic and epigenetic links to BD in the immune system. Importantly, a large proportion of these genes were linked to BD only by genetic and epigenetic evidence in blood, suggesting that immune function dysregulation in circulating cells may contribute to BD pathogenesis independent of resident immune cells of the brain. We also subtyped patients based on blood epigenomic signals into five subgroups, four of which had strong enrichment of BD-related medical terms. By analyzing the genetic basis of BD patient heterogeneity, we found that BD genetic signals associated with dysfunction in circulating immune cells could capture epigenomic and phenotypic variations among patients. Drug repurposing analysis performed by testing the enrichment of BD epigenetic signature genes in the differential gene expression profiles of hematopoietic cell lines in response to pharmacological perturbation revealed potential peripheral effects of BD drugs and identified drugs/compounds that could ameliorate BD-associated immune dysregulation, including agents with predicted selective effects in patients with or without inflammatory epigenetic profiles. These results highlight that blood epigenomic signals can reflect the physiological state variation of BD patients and are, therefore, promising as biomarkers that could facilitate precision medicine in BD such as matching patients to the right medications.

Immune dysregulation has previously been noted in psychiatric diseases, particularly in bipolar disorder^111^. For example, increased levels of pro-inflammatory cytokines such as IL-1β, IL-6, and TNF-α have been reported in patients with BD^112^ and genetic studies have revealed correlation between BD and autoimmune disorders, such as celiac disease and Crohn’s disease^113^. However, these studies were limited by reliance on a single modality, and the causal relationship between immune dysfunction and BD remained unclear. To address this problem, we tracked the effects of BD-associated genetic variants in immune dysfunction, leveraging multiple data types in the circulating immune system. Most of the BD-associated genetic variants based on GWAS are in the non-coding regions^114^, which makes it challenging to track their actions through their target genes in various cell and tissue types. We applied the following six strategies to address this challenge: (1) Global enrichment: we analyzed genome-wide enrichment of disease-associated genetic variants in CREs primarily active in specific tissue or cell types^29,115^. We highlighted distinct enrichment patterns between immune-related traits (e.g., autoimmune diseases, Alzheimer’s disease, and immune markers) and psychiatric disorders (e.g., BD, SCZ, and MDD). While the former showed strong enrichment in immune-specific regulatory elements, psychiatric risk variants appeared to impact enhancer programs that are active across both brain and circulating immune cells, pointing to a more systemic but less lineage-specific immune involvement (Fig. 3b). (2) GWAS–QTL integration: we integrated GWAS signals and molecular QTLs for histone modifications (Fig. 3c, d) and gene expression (Fig. 4c, d) to specify CREs and genes where a given disease-associated genetic variant can exert its effect at the molecular level. (3) Regional regulation: focusing on chromatin interaction blocks with BD-associated gCREs, we found that these chromosomal units enriched BD dCREs in blood, supporting that immune dysregulation in BD may stem from BD genetic variants (Supplementary Fig. 7b). (4) CRE–gene linking: we identified driver genes by linking both BD genetic signals and differential signals through two CRE–gene linking strategies, namely, Hi-C and gLink scores (Fig. 4a). (5) Blood-brain comparison: we compared GWAS–eQTL colocalization for driver genes between blood and brain, decoupling the genes specific to blood from those shared between the two tissues, permitting the identification of BD-relevant immune dysfunctions encoded exclusively in circulating white blood cells (Fig. 4c, d). (6) EHR phenotypic validation**:** Finally, we leveraged clinical data to test whether patient subgroups defined by epigenomic profiles were associated with immune-related phenotypes. Indeed, inflammation-related EHR terms such as *leukocytosis* and *joint pain* were enriched in specific BD subgroup (group 1), providing clinical support for the immune dysregulation hypothesis. Our systems strategy of integrating information based on genetics and epigenetics provides evidence of the impact of BD-associated genetic signals on the immune system, highlighting candidate SNP–CRE–gene circuits, especially those specific to the circulating immune system, that link immune dysregulation to BD.

Our study provides a mechanistic view of how immune components are involved in BD pathogenesis and affected by BD medication. We discovered upregulated innate immune response and downregulated adaptive immune response pathways in BD, consistent with previous findings of regulatory T cell anergy and senescence in BD^116^. We identified TFs responsible for these alterations, such as FLI1, and their potential connections to BD genetic signals or differential signals in blood. Furthermore, we also identified differentially regulated CREs that may be affected by BD medications, indicating that these drugs may modulate the immune system in distinct ways. We also captured additional upstream CREs inferred from genetic signals. These gCREs, whose hQTL signals were colocalized with BD signals, may indicate a causal effect in circulating immune cells on BD. They are linked based on genetic evidence or chromatin interaction to ER homeostasis and calcium signaling-related genes, including *TMEM258*, *GRINA*, *ORMDL3*, *LMAN2L*, *MAP1LC3A*, *ITIH4*, *MCHR1*, *RAB1B*, *Y1F1A*, and *CD47* (Fig. 4d). For example, *ORMDL3*, an asthma-associated gene, is known to promote inflammation through ER stress and unfolded protein response^117,118^. Importantly, calcium signaling and ER stress were also shown to be connected to the BD downstream events of T cell energy and exhaustion^119,120^. Consistently, we also identified cardiovascular drugs, e.g., nifedipine, targeting calcium signaling pathways, which are predicted to reverse BD associated immune dysregulation (Fig. 6c). Additionally, we found other potential BD driver genes in the circulating immune system, including genes related to epigenetic regulation (*SFMBT1* and *ANP32E*), apoptosis/DNA damage (*XPNPEP3*, *NRBP2*, *PLEKHO1*, and *PARP10*), autophagosome (*MAP1LC3A*), anti-phagocytosis (*CD47*), and fatty acid metabolism (*FADS1*) (Supplementary Data 5).

We next investigated the potential of blood as a surrogate tissue for studying psychiatric disorders. The importance of “liquid biopsies” has been increasingly recognized for their potential for identifying biomarkers in easily accessible tissues. Several studies have been conducted to establish connections between the blood and brain at various molecular levels, such as gene expression, DNA methylation, plasma cell-free DNA methylation, or QTL signals^121–125^. Our findings revealed three potential mechanisms connecting the circulating immune system to psychiatric disorders such as BD: (1) immune dysfunction manifest in BD, such as upregulated innate immunity and downregulated adaptive immunity (Fig. 2a); (2) BD-genetics-driven alterations specifically in the immune system, such as genes related to calcium signaling and ER-related transport, potentially exacerbating BD pathogenesis (Fig. 4d); and (3) events in the immune system mirroring those in the brain in BD due to shared genetics or epigenetic regulations (Fig. 4d). This third mechanism is currently largely under-studied. Specifically, our analysis of cell-type-specific GWAS–eQTL colocalization revealed that a subset of driver genes, some also related to calcium signaling and ER-related transport, were shared between immune cells and specific neuronal cell types: excitatory pyramidal cells of neocortical layers 2/3 (Ex-L2or3) or layer 4 (Ex-L4). These neuronal cell types have been previously implicated in stress-induced depressive behaviors. Interestingly, these genes were not shared with microglia, the primary brain immune cell type. Consistently, ER homeostasis and calcium signaling are also central to neuron homeostasis in the brain and are highly relevant to BD. For instance, studies have shown that disruptions in calcium signaling pathways may contribute to BD pathogenesis by affecting neuronal excitability, plasticity, and survival^126^. These three mechanisms provide various perspectives for identifying biomarkers in blood for psychiatric disorders.

We also used our blood profiles of five histone marks to reveal distinct patient subtypes in our type I BD cohort, without accessing any clinical information. We grouped all patients using 24 epigenomic LFs and identified five groups by thresholding the hierarchical cluster tree (Fig. 5a). Notably, clusters 1 and 4 were enriched for BD cases, while cluster 5 was enriched for controls (Fig. 5b), suggesting that the subtyping partially recapitulates diagnostic status. However, the clusters also captured phenotypic and biological heterogeneity beyond diagnosis, as evidenced by their enrichment for diverse phenotypic, clinical, and treatment features, highlighting the clinical relevance of our findings (Fig. 5c). To distinguish whether these epigenomic LFs were solely a consequence of treatment, or perhaps drivers of disease subtypes leading to treatment differences, we investigated the genetic basis of these patient subgroups. We partitioned the BD PRS by tissue-specific CRE groups or blood gCREs associated with BD genetics and found that the latter (Blood_gAREs_BD.Coloc and Blood_gAREs_BD.MR) could explain inter-individual variation among BD patients including the reactivation of cytomegalovirus (Fig. 5d), revealing a genetic component in the previously reported association between CMV reactivation and BD^87^. These two PRSs also distinguished subgroups 2 and 3 from the rest of the patients. Interestingly, other BD PRSs seem to capture other aspects of patient heterogeneity, for example, whether patients are currently taking lithium or aripiprazole. These findings provide additional insights into the potential molecular basis of BD subtypes and their connections to various risk factors and treatments and indicate that genetic signals associated with dysfunction in circulating immune cells can capture epigenomic and phenotypic variations among patients, further supporting a role for immune dysregulation in circulating cells in BD pathogenesis and the utility of analyzing peripheral blood cells to obtain mechanistic insights into BD and to develop biomarkers for patient classification.

We finally established a link between peripheral immune cell epigenomics and drug responses in BD. CREs across histone marks in blood in response to patient medications from the EHR revealed specific associations of common BD medications with adaptive and innate immune system pathways and inflammation (Fig. 6a), supporting the findings that these drugs, besides targeting central nervous system functions, may also have direct effects on circulating immune cells^127,128^. Inspired by this finding, we reasoned that drugs or compounds reversing the immune dysregulation may potentially be repurposed for BD. We thus carried out drug repurposing analysis by testing the enrichment of the blood gene sets with BD up- or down-regulated epi-signatures in the differential expression profiles of hematopoietic cell lines treated by pharmacological agents in CMap. The analysis revealed 428 compounds that repressed genes with BD up-regulated signatures and activated genes with BD down-regulated signatures, suggesting that they could ameliorate BD-associated immune dysregulation (Fig. 6b). Further refining these studies by considering patient groups with or without inflammation-related EHR signatures uncovered subnetworks of drugs/compounds and drug targets related to the nervous system including well-established BD drug targets such as serotonin receptors and transport mechanisms (Fig. 6c), consistent with the result of BD medicine response analysis mentioned above. The subnetworks also include tyrosine kinases with roles in immune cells and cancers, and cardiovascular drugs including some with potential therapeutic effects in BD. Interestingly, the non-inflammatory patient group showed a higher proportion of cardiovascular drugs relative to the inflammatory group (Supplementary Table 2). Unlike direct target–based approaches, this regulatory reversal–based strategy prioritizes compounds that counteract disease-associated gene programs inferred from epigenomic changes. For compounds that directly target epigenetic or transcriptional regulators, e.g., CDK7, CDK9, or Aurora kinases (Fig. 6c), this approach may also help elucidate mechanisms of action by revealing their effects on regulatory pathways.

Our study has several limitations. First, we could not include gene expression data since the common protocol used for the biobanking of buffy coat samples was designed to preserve DNA, not RNA. However, we overcame this by using epigenomic signals, especially H3K36me3, a mark for the gene body, to infer differential gene expression by meta-analysis. Second, our analyses were not performed at the single-cell or cell-type level. Nevertheless, we used deconvolution analysis to ensure that cell fraction changes were accounted for during our differential signal detection and QTL mapping. Third, although our hQTL analysis captured genetic effects across the full cohort, hQTL effect sizes may differ between BD cases and controls, offering insights into how BD may alter genetic risk for other diseases. This represents an important future direction enabled by the genotype and epigenomic data we provide. Fourth, we did not have access to paired brain postmortem samples to check the brain–blood sharing of the differential signals. In future studies, a paired-sample design would be more powerful to detect blood biomarkers that recapitulate changes in the brain. Fifth, our patient subtyping and enrichment analyses are limited by small sample sizes in certain subclusters, reducing statistical power to detect associations with clinical traits or treatment exposures, such as lithium, that may drive meaningful subgroups. Larger cohorts will be required to robustly capture these effects. Sixth, we also did not have drug response or other longitudinal data. We plan to integrate these data types in future work to better understand whether our subtyping is stable throughout an individual’s lifespan and meaningful for predicting drug response. Seventh, our work only addressed drug effects in the immune system. A more accurate prediction would require integrating the peripheral and central nervous system responses. We also noticed that some of the suggested drugs might lead to detrimental, rather than beneficial effects on BD. For example, flutamide may induce manic-like episodes^129^ (Supplementary Fig. 11d) and nicotine could exacerbate manic episodes in individuals with BD^130^ (Supplementary Fig. 11e). Conversely, peripheral blood epigenomic profiling may also be useful for linking medications to adverse effects on BD. Finally, our study cohort consists primarily of non-Hispanic White individuals diagnosed with Type I bipolar disorder. While this enhances the accuracy of PRS-based analyses and reduces heterogeneity within the cohort, it also limits the generalizability of our findings to other ancestral populations and to individuals with type II BD.

Despite the limitations, our study represents a significant step toward understanding the complex relationships between the blood and brain in psychiatric disorders such as BD. Studies like ours are essential for understanding the immune-psychiatry axis. By integrating multimodal data and investigating the immune components of BD from both genetic and epigenetic perspectives, this work offers valuable insights into the potential role of circulating immune cells in BD pathogenesis. Furthermore, our findings on BD subtypes based on blood epigenomic signals pave the way for improved patient monitoring and personalized treatment recommendations. Our datasets also offer a unique epigenomic modality that complements existing blood transcriptomic and methylation-based studies and opens avenues for cross-tissue and cross-modality comparisons.

## Methods

### Mayo clinic participants

The 180 participants (88 type I BD cases and 92 controls, 107 females and 73 males) were from the Mayo Clinic Bipolar Disorder Biobank and the Mayo Clinic Biobank^131^, respectively. For control participants, subjects from the Mayo Clinic Biobank were matched on age and sex with BD cases and filtered based on psychiatric conditions in self-reports and/or EHR. The EHR information for each of the patients covered five categories, including ICD-9 codes, procedure codes, patient-provided information, lab tests, and medication prescriptions. EHR information is not shared due to privacy concern. All participants had consented to research, including research using their EHR data^23^, as we as to share their individual genomic data and demographic information, including age and sex. Sex and age were determined based on clinical records and were considered as a biological variable in the analyses where applicable. This study was reviewed and approved by the Mayo Clinic Institutional Review Board and by the access committees from both biobanks. Participants were compensated with gifts less than $20 for this study.

### Ethical compliance statement

All human studies were designed and conducted in accordance with relevant institutional, national, and international guidelines and regulations governing research involving human participants, including the principles outlined in the Declaration of Helsinki. The study protocol was reviewed and approved by the Mayo Clinic Institutional Review Board.

### Common variants from whole-genome sequencing (WGS)

WGS of our cohort (*N* = 179 with one individual missing) were carried out using the same experimental protocol and quality control procedure as in a previous study^132^. In short, DNA from blood samples underwent shearing using a Covaris LE220 ultrasonicator to obtain fragments of about 350 bp in size. The fragmented DNA was processed using the NEBNext® DNA Library Prep Master Mix Set tailored for Illumina®. Unique in-house primers were employed to barcode the post-ligated samples, which were subsequently amplified over 6 PCR cycles with the KAPA HiFi HotStart Ready Mix. Library concentrations were gauged using the Qubit® 2.0 Fluorometer. Meanwhile, the quality of these libraries was assessed using a DNA 5 K chip in conjunction with a Caliper GX. Precise quantification was ascertained via the qPCR-based KAPA Biosystems Library Quantification kit. Each sample underwent sequencing on a single lane of Illumina’s HiSeq X device using v2 flow cells and reagents to achieve 30X genomic coverage.

The derived fastq files were then channeled through the *Mayo Genome GPS* v4.0 system. Using the Burrows–Wheeler Aligner^133^, reads were aligned to the human reference sequence (GRCh38 build). The Genome Analysis Toolkit (GATK)^134^ was employed for local realignment surrounding indels. Variant identification was executed using the GATK HaplotypeCaller, followed by variant recalibration (VQSR) in line with the GATK’s recommended best practices^135,136^. Genotypic determinations with GQ below 10 and/or depth (DP) lesser than 10 were omitted. Variants with an ED exceeding 4 were excluded from further analyses. We retained only those variants that cleared the VQSR and exhibited a call rate surpassing 95%. Ultimately, our study concentrated on 6,790,411 common variants with a minor allele frequency greater than 5%.

### ChIP-seq experiment

Buffy coat samples isolated from 5-10 ml of peripheral blood were preserved in anti-freeze media (90% fetal bovine serum and 10% dimethyl sulfoxide) at −80 °C. The samples were thawed on ice, cross-linked with formaldehyde (final concentration: 1%) for 10 min at room temperature then quenched with 125 mM glycine for 5 min. Fixed cells were washed twice with 1X TBS and lysed in cell lysis buffer (10 mM Tris-HCl, pH 7.5, 10 mM NaCl, 0.5% IGEPAL) on ice for 10 min. The lysates were washed with 1 ml MNase digestion buffer (20 mM Tris-HCl, pH 7.5, 15 mM NaCl, 60 mM KCl, 1 mM CaCl_2_), resuspended with 500 μl of MNase digestion buffer, and incubated for 20 min at 37 °C in the presence of MNase (New England Biolabs, Ipswich, MA). After adding the same volume of sonication buffer (100 mM Tris-HCl, pH8.1, 20 mM EDTA, 200 mM NaCl, 2% Triton X-100, 0.2% sodium deoxycholate), the lysate was sonicated for 30 min (30 s on, 30 s off) in Diagenode bioruptor and centrifuged at 21,000 *g* for 10 min. The supernatant was divided into five microcentrifuge tubes and incubated with ChIP-seq-validated antibodies on a rocker overnight. The following antibodies were used in the experiment; anti-H3K27ac (CST #8173), anti-H3K4me1 (Abcam ab8895; CST #5326), in-house generated anti-H3K4me3 (EDL lot 1)^137^, anti-H3K27me3 (CST #9733), and anti-H3K36me3 (Active Motif #61101) (Supplementary Table 1).

Immunoprecipitation and library preparation were performed as described^137,138^, ensuring robust experimental consistency across batches. After adding 30 μl of prewashed protein G-magnetic beads, the reaction was further incubated for 3 h. The beads were extensively washed with ChIP buffer, high salt buffer, LiCl 2 buffer, and TE buffer. All washes were carried out for 5 min at 4 °C on a rotating wheel. Bound chromatin was eluted in 100 μL ChIP elution buffer (10 mM Tris-HCl, pH 8.0, 10 mM EDTA, 150 mM NaCl, 5 mM DTT, 1% SDS) and reverse-crosslinked at 65 °C overnight. After treatment with RNase A and proteinase K, DNA was purified by Qiagen MinElute PCR Purification Kit (Cat. # 28006, Valencia, CA) and quantified using Qubit dsDNA High Sensitivity assay (Invitrogen, Q32851). ChIP yield, the DNA amount obtained after ChIP divided by the DNA amount of total chromatin input, was calculated. To check the size of input chromatin, purified input DNA was analyzed by Fragment Analyzer (Advanced Analytical Technologies; AATI; Ankeny, IA) using the High Sensitivity NGS Fragment Analysis Kit (Cat. # DNF-486). ChIP-seq libraries were prepared for input and ChIP DNAs for each sample using the ThruPLEX® DNA-seq Kit V2 (Rubicon Genomics, Ann Arbor, MI) according to the manufacturer’s instructions. To avoid PCR amplification bias, we determined the number of amplification cycles of each library by analyzing the DNA amount from the fraction of the PCR reaction after 12 cycles of PCR amplification of the adaptor- ligated DNA by the Fragment analyzer. The libraries were sequenced to 51 base pairs from both ends on an Illumina HiSeq 4000 instrument in the Mayo Clinic Center for Individualized Medicine Medical Genomics Facility.

### Quality control of ChIP-seq and preprocessing

We carried out peak calling with the ENCODE pipeline (https://github.com/kundajelab/chipseq_pipeline). Briefly, we mapped reads to human genome assembly hg19 (*bwa*, v0.5.9)^133^, filtered low-quality and multiple-mapped reads by *samtools* (v1.3.1)^139^, and applied *MACS2* (v2.1.1)^140^ for peak calling. Samples were then filtered based on three QC metrics: (a) Relative Strand Cross-correlation coefficient (RSC), a key ChIP-seq QC on signal/noise; (b) total reads; and (c) correlation between the signal of samples and those from blood samples in Roadmap reference epigenome. We define “Tier 1” samples as those with RSC score ≥2, total reads ≥30 M, and for which their matched Roadmap tissue was in the top three most correlated Roadmap epigenomes. We defined “Tier 2” samples with relatively permissive cutoffs: RSC score ≥1, total reads ≥20 M, and the same criteria of matched-tissue correlation.

We used high-quality samples of “Tier 1” to generate a reference peak set for each histone mark. We first selected peaks (q-value ≤ 0.05) from each sample and then merged them together. Only peaks present in at least two individuals and overlapping with peaks from immune cell samples in Roadmap are kept in consensus peaks sets for each histone mark, resulting in 156,967 (H3K27ac), 247,775 (H3K27me3), 161,248 (H3K36me3), 211,919 (H3K4me1), and 57,811 (H3K4me3) peaks, defined as *cis-*regulatory elements (CREs).

We extracted peak intensity as CRE activity across all “Tier 2” samples for downstream analyses. We defined peak intensity as the number of reads bases within each peak as in a previous report^70^. Peak intensity was then normalized with three steps: (1) to take into consideration library reads depth, first peak intensities are normalized with a factor for each sample to reach 30 M total reads across all the peaks identified; (2) peak intensity mean is then defined by peak intensity divided by peak length, and further normalized across samples by dividing the size factor inferred from R package DEseq2 (v_1.20.0)^141^, 3) finally peak intensity mean is corrected for GC content using R package gcapc (v1.4.0)^142^.

### CRE modules and functional annotation

We investigated the co-activity pattern of 223,573 CREs annotated by three enhancer/promoter-associated marks (H3K27ac, H3K4me1, and H3K4me3) across reference epigenomes based on H3K27ac intensity as in a previous study^70^. Briefly, we extracted -log_10_P signal from 242 EpiMap H3K27ac samples for each CRE (bigWigAverageOverBed^143^, v2), binarized the matrix by a cutoff of 2, kept CREs with non-zero values in at least one example (*N* = 168,801), and clustered them by a k-centroids algorithm implemented in R package *flexclust* (V1.4-0)^144^ with the Jaccard index as the distance metric and cluster number set as 84 (corresponding to 2000 CREs per module on average). We then aggregated CRE modules into 13 groups with multiple steps: (1) defining ubiquitous score as the proportion of samples with average CRE activity ≥0.2 for each CRE module; (2) defining CRE modules as Broadly-active (G1) if their ubiquitous scores were above 0.5; (3) defining CRE modules as Multi-tissue (G2) if their ubiquitous scores were between 0.1 and 0.5; (4) clustering 242 reference samples into 11 clusters based on average CRE signals across 84 modules by hierarchical clustering with 1-Pearson correlation coefficient as the distance metric, naming each sample cluster manually based on the names of majority samples; and (5) grouping the rest of the CRE modules based on the sample cluster where the CRE module shows the strongest signal. The CRE modules were visualized by R package *ComplexHeatmap* (v2.4.3, R-4.0)^145^ in Fig. 1c.

We annotated CRE modules based on different functional categories: (1) genomic regions: annotation calculated by R package *annotatr* (v1.6.0)^146^ (Fig. 1c); (2) TF binding sites: we used position weight matrix (PWM) of binding sites for TF families collected in EpiMap and scanned ARE modules for enriched motifs by Homer (v4.11.1)^37^ with background auto-selected; we filtered TF motifs based on adjusted *P*-value after Benjamini-Hochberg (BH) correction (cutoff of 0.05) and odds ratio (cutoff of 1.2); we visualized the enrichment for each TF family (mean odds ratio) in promoter and enhancers (Supplementary Fig. 1b, c); and (3) biological processes from gene ontology (GO): enrichment of nearby genes is calculated by R package rGREAT (v1.2.1)^147^ with the CREs across all modules as background (Supplementary Fig. 1d).

### Cell-type fraction estimation

We estimated fractions of six major white cell types, including CD4^+^ T cells, CD8^+^ T cells, B cells, natural killer cells, monocytes, and neutrophils in our bulk blood samples based on CRE activity from reference cell-sorted profiles. We carried out the analysis for each of three histone marks with strong cell type-specific signals (H3K27ac, H3K4me1, and H3K36me3) in four steps: (1) normalization of CRE activity including reference cell-sorted samples from Roadmap^26^ and BLUEPRINT^73^ and our samples described as above; (2) identification of cell-type marker CREs based on activity across reference samples, and then filtering marker CREs for each cell type based on correlation among each other in our bulk dataset, a strategy applied in a previous report^148^; (3) randomly sampling the same number of marker CREs (*N* = 30 for H3K27ac and H3K4me1, and 15 for H3K36me3) from the whole marker CRE set for each cell type for 100 times and estimating cell fractions for each of our bulk samples based on activity of marker CREs from each sampling with non-negative Poisson regression in R package addreg (v3.0)^149^: ${y}_{j}={\sum }_{i=1}^{6}{{\mathrm{Fract}}}_{{{\rm{i}}}}\,\times \,{{\mathrm{Ref}}}_{i,j}$, where ${y}_{j}$ denotes activity in a bulk sample for *j-th* CRE, ${{\mathrm{Fract}}}_{i}$ denotes the cell fraction of the bulk sample for *i-th* cell type such that ${{\mathrm{Fract}}}_{i}\,\ge 0$, and ${{\mathrm{Ref}}}_{i,j}$ denotes activity of *j-th* CRE in *i-th* cell type; (4) calculation of the average of resultant cell fractions estimated from each sampling as the final estimation.

### Differential CRE analysis and motif enrichment

To identify dCREs associated with BD, we first filtered CREs with at least 5 counts in at least 20% of samples in the normalized count matrix. We then applied surrogate variable analysis with R package sva (v3.28.0)^150^ followed by a linear regression model with R package limma (v.3.36.5)^151^, to account for the hidden variables (the inferred surrogate variables). The hidden variables could explain a significant proportion of the variance of residuals after regressing out the known variables, including sex, age, and disease status. We found the inferred surrogate variable was strongly correlated to the estimated cell fractions. We finally defined BD dCREs as those passing the adjusted *P*-value cutoff (0.05) after BH correction. We used the position weight matrix (PWM) of binding sites for TF families collected in EpiMap, scanned dCRE for each histone mark for enriched motifs by Homer (v4.11.1)^37^ with background auto-selected, and filtered TF motifs based on adjusted *P*-value (cutoff of 0.05) and odds ratio (cutoff of 1.2). To identify CREs associated with drug response, we included drugs taken in at least five individuals and used the same strategy as above while including other drugs taken as covariates. We finally defined dCREs associated with drug response as those passing adjusted *P*-value cutoff (0.2) after BH correction.

Due to logistical constraints, cases and controls were processed in separate batches, with only 2 of 62 containing both. To evaluate whether batch structure confounded differential analyses, we performed several checks. First, permutation of batch labels (case vs. control) showed a significant reduction in the number of differential CREs across 500 iterations, indicating the observed signals were unlikely to arise from batch effects (Supplementary Fig. 4a). Second, differential comparisons between individual samples and opposing group batches produced more consistent CRE-level signals than within-group comparisons (Supplementary Fig. 4b). Third, principal component analysis of model residuals showed no clustering by batches (Supplementary Fig. 4c). Together, these analyses suggested that batch effects might still impact differential results, however, were unlikely to be the main driver of the observed BD vs. control differences.

Enrichment of nearby genes for dCRE associated with BD or drug response is calculated by R package rGREAT (v1.2.1)^147^ with all CREs detected for each histone mark as background (Figs. 2a, 6a). We only kept terms with more than 10 genes and less than 500 genes and listed top 15 terms for each dCRE set.

### Genes with BD differential epigenomic signatures

We identified genes with BD differential epigenomic signatures based on the BD differential signal for each histone mark on the gene body and gene regulatory domain of a gene. We extracted information about the gene body from GENCODE (v26)^152^. We defined gene regulatory domain as CREs linking to TSS of a gene based on any chromatin loops inferred from promoter-capture Hi-C data of sorted circulating immune cell types from BLUEPRINT^71^, and filter any CREs overlapping with the gene body. We then aggregated differential signals of CREs over gene body and gene regulatory domain for each gene for each histone mark based on meta-analysis implemented in R package meta (v4.9-5) into z scores. We flipped the sign of z scores related to H3K27me3 since it is a repressive mark. With gene body H3K36me3 well accepted as a transcription signature, we found other scores including gene body H3K27ac, gene body H3K27me3, gene regulatory domain H3K27ac, and gene regulatory domain H3K36me3 to be strongly correlated with it. We thus included all five scores to define the genes with BD differential epigenomic signature with the following criteria: (1) at least one score passes the adjusted *P*-value cutoff (BH correction, 0.05); (2) the score for gene body H3K36me3 passes the permissive nominal *P* cutoff of 0.01; (3) at least two scores pass the nominal *P* cutoff of 0.01 and they should have a consistent sign of differential signal.

### GWAS signal enrichment in ARE groups by LDSC

We applied LD score regression (v1.0.0)^63^ with default parameters to estimate the enrichment of GWAS signals of multiple traits for different CRE groups. The GWASs included those based on UK biobank^153^, e.g., rheumatoid arthritis, inflammatory bowel disease, Multiple Sclerosis, Parkinson’s disease, Type 1 diabetes, Type 2 diabetes, Neuroticism, CRP level^154^, and neutrophil counts^155^, those from Psychiatric Genomic Consortium, e.g., bipolar disorder^156^, schizephrenia^157^, Major depressive disorder^158^, and cross disorder^52^, as well as Alzheimer’s disease^159^, high blood pressure^160^, smoking initiation, drink per week^161^, and height^162^. We carried out BH correction for testing multiple CRE groups for each trait (adjusted *P* cutoff 0.25).

### hQTL mapping

We adapted the pipeline of our previous eGTEx study^70^ for histone QTL mapping. Briefly, we inferred the latent factors in our samples for each histone mark by Peer (R package v1.0)^61^, and identified the optimum number of PEER factors (10 for all marks) to correct for. We confirmed that QC metrics such as fraction of reads in peaks (FRiP) and RSC as well as estimated cell fractions are highly correlated with these latent factors, thus these were considered during QTL calling (Supplementary Fig. 6a). We also added age and sex as covariates.

To assess the impact of population structure, we performed principal component analysis (PCA) on the genotype matrix and found minimal genetic variation (top two PCs explained <1.5% of variance). Inclusion of the top five genetic PCs as covariates in hQTL mapping yielded nearly identical effect sizes and test statistics (Pearson *r* > 0.999), indicating that population stratification had minimal impact. Therefore, genetic PCs were not included in the final hQTL analysis.

To ensure alignment with the genotype matrix in GRCh38, we used UCSC LiftOver^163^ to map CREs defined by various histone marks from hg19 to GRCh38. We then applied *FastQTL* (v2.184)^164^ to map hQTLs within 100 kb to the center of each CRE on autosomes for common variants with minor allele frequency ≥0.05. We applied a two-step multiple test correction: (1) first estimated an empirical *P*-value for each CRE based on the lead nominal *P*-value and permutation results (--permute 1000 10000) by fitting a beta distribution to account for multiple variants tested with *FastQTL*, and (2) calculated q-value for the empirical *P*-value to account for multiple CREs tested with R package *qvalue* (v2.12.0), defined gCREs based q-value cutoff of 0.05, and defined hQTL for each CRE with corresponding nominal *P*-value threshold to the q-value cutoff 0.05.

### hQTL sharing across histone marks and between the blood and brain

We followed the strategy used in our eGTEx studies^62^ to quantify QTL sharing between two histone marks in our blood data. Briefly, for each pair of discovery mark and replication mark, we checked the lead hQTL signal for each gCRE based on the discovery mark for the replication mark and showed patterns of increasing directionality consistency between both marks with stronger nominal *P*-value signal. We simply assumed that non-shared QTL events lead to a 50%–50% chance to show consistent sign or not between marks, thus we estimated the proportion of overall sharing as the proportion of directionality inconsistency deducted from that of directionality consistency between two marks (Supplementary Fig. 6d). We applied the same strategy to calculating the sharing of H3K27ac between blood and brain, with a permissive q-value cutoff of 0.2 to define gCREs in the blood (Supplementary Fig. 6f).

### Single-cell eQTL analysis

Based on the cell-type annotation results for the 14 major brain cell types in an ROSMAP cohort^74^, we constructed pseudobulk expression profiles from snRNA-seq by estimating the log-mean parameters of a Poisson-Gamma hierarchical model (https://github.com/causalpathlab/mmutilR). We first filtered in genes expressed in at least 30% of individuals while excluding individuals with fewer than 20 cells per cell type. For each cell type, we have an individual-by-gene expression matrix and quantile normalized across genes, following the pipeline recommended by the GTEx project^60^. In order to adjust putative population-level non-genetic confounding effects, we also performed principal component analysis with *K* = 77, which was found by cross-validated correlation of the polygenic prediction model. Within a ± 1 Mb window around each protein-coding gene’s boundary from the transcription start site to the end site, we calculated marginal eQTL (SNP-by-SNP) statistics by regressing common variants (MAF > 5%, genotyping rate >95%, HWE *P* > 10⁻⁶) on the normalized expression vectors. The whole pipeline is accessible here (https://github.com/YPARK/brain-eqtl).

### GWAS-hQTL, hQTL-eQTL, and GWAS-eQTL colocalization and Mendelian randomization (MR)

Since the Mayo Clinic cohort is part of the Psychiatric Genomics Consortium (PGC) BD GWAS study^85^, we regenerated and used BD GWAS without the Mayoc Clinic cohort for all the downstream analyses. To increase sensitivity in identifying potential regulatory variants that colocalize with hQTLs, we applied a subthreshold cutoff for GWAS (*P* ≤ 1 × 10^−6^) and a permissive q-value cutoff of 0.2 to define gCREs for colocalization analysis and Mendelian randomization. For both analyses, we only included gCREs within 100 kb of any GWAS signal passing subthreshold cutoff (*P* ≤ 1 × 10^−6^) or eQTL signal (defined in GTEx). We used coloc (v3.2-1)^64^, a Bayesian framework, to estimate the pairwise posterior probability (PP4) of causal SNPs colocalized among GWAS, haQTL, and eQTL signals based on summary statistics. We calculated enrichment of nearby genes for merged colocalized gCREs (PP4 > 0.5) by R package rGREAT (v1.2.1)^147^ with the gCREs from all histone marks as background. We also tried a more stringent cutoff PP4 > 0.7, which led to consistent enrichment results as in Fig. 3C. We applied Mendelian Randomization, specifically MR-Egger implemented in R package MendelianRandomization (v0.4.1)^165^, to infer the causal effect of CRE activity on either gene expression or phenotypic risk based on haQTL/eQTL/GWAS summary statistics. We filtered SNPs requiring its hQTL effect (adjusted hQTL *P*-value cutoff of 0.2, by BH test carried out for all SNPs in the locus) and pruned by r^2^ (cutoff 0.6) estimated from our cohort by PLINK (v1.9)^166^. We also summarized the BD GWAS loci that could be associated with our gCREs based on GWAS-haQTL colocalization. We clumped BD GWAS SNPs (*P* ≤ 1 × 10^−6^) with PLINK (v1.9, --clump-r2 = 0.2, --clump-kb = 250) into 203 independent loci and identified 97 of them could be associated with the colocalized gCREs above through hQTL (q-value cutoff of 0.2) (Supplementary Data 4).

### Gene regulatory domain and BD driver genes prioritization

We applied two strategies to define the gene regulatory domain, or CREs linking to a specific gene: (1) physical interaction-based evidence: we aggregated chromatin loops from promoter-capture Hi-C data across multiple immune cell types from BLUEPRINT^71^ and linked a CRE to a gene if its connection to any TSS of the gene was supported by chromatin loops; and (2) genetics-based evidence: we included four of gLink scores defined in our eGTEx study^70^, (a) gARE-dist-to-FMeQTL: the shortest distance between FM-eQTLs of an eGene to a specific gCRE; (b) coloc-PP4: the coloc PP4 between hQTL of a gCRE and eQTL of an eGene; (c) coloc-PP4/3: the ratio of PP4 to PP3; (d) MR: adjusted P-value (BH) for MR-Egger test with hQTL and eQTL summary statistics.

We defined all the gCREs within 100 kb of eGene TSS as candidate CREs and evaluated gLink scores’s performance to predict physical-interaction-based links with AUPRC, finding that top gLink scores enriched positive links. We permuted gLink scores within each CRE–gene distance bin 50 times and evaluated performance using the area under the precision-recall curve (AUPRC). The AUPRC based on the original gLink scores was consistently higher than that from the permuted scores, indicating improved predictive power over random expectation (Supplementary Fig. 7c). Both results indicated the two CRE-gene linking strategies above were consistent with each other and thus were used for the downstream analysis to define driver genes.

We defined BD driver genes as genes that are susceptible to upstream genetic signals and impact downstream changes during BD pathogenesis. Thus, we prioritized those genes whose gene regulatory domain—derived from either promoter-capture Hi-C data or gLink scores—included both gCREs sharing genetic signal with BD based on colocalization (coloc PP4 cutoff 0.5) or MR analysis (nominal *P* cutoff 0.01), a sign of acting in the upstream genetic susceptibility, and dCREs acting downstream of BD (adjusted *P* cutoff 0.05, BH correction).

### Latent factor analysis and annotation

We filtered dCREs with robust signals based on chromatin loops for downstream latent factor analysis. We first defined Hi-C blocks as groups of genomic regions linking with each other through chromatin loops no longer than 1 Mb. We found that as the density of links in the Hi-C blocks increased, the enrichment of differential peaks also increased (Supplementary Fig. 7a). The enrichment pattern is quite consistent across marks and also likely to be supported by BD GWAS signal (Supplementary Fig. 7b). We thus only focused on dCREs (nominal *P* cutoff 0.01) in the Hi-C blocks show significant enrichment (one-sided Fisher’s exact test, BH correction, adjusted *P* cutoff 0.1) as robust differential signals, and inferred latent factors across all individuals from all five marks with R package MOFA (v1.6.2, numFactors =40, dropFactorThreshold =0.005). We then annotated these factors by revealing the significant correlation (adjusted *P* cutoff 0.05, BH correction across all terms, R^2^ cutoff 0.1) of latent factors with EHR and cell fraction estimated across BD patients (Supplementary Fig. 8c).

### Patient subtyping, annotation and marker CREs

We calculated the Pearson’s correlation coefficients across latent factors among all individuals and clustered participants into five groups based on the correlation matrix with hierarchical clustering (R function *hclust*, Fig. 5a). To assess clustering robustness, we conducted a subsampling-based (90% samples) stability analysis using the Adjusted Rand Index (ARI) from R package mclust (v.5.4.3) (Supplementary Fig. 8d), which supported the selection of five clusters for downstream analysis. We next identified EHR terms enriched in each patient group compared to the rest of patients (one-sided Fisher’s exact test for categorical terms, one-sided *t*-test for numerical terms, nominal *P* cutoff 0.02, Fig. 5c), and visualized it with R package ComplexHeatmap (v2.4.3, R-4.0)^145^.

We only focused on the dCREs with robust signal above (nominal *P* cutoff 0.01, and within Hi-C blocks enriched for dCREs, adjusted *P* cutoff 0.1) for marker CREs detection. We applied linear regression to detect marker CREs for each patient group compared to the rest of the patients with sex and age as covariates (adjusted *P* cutoff 0.05, BH correction). We then annotated marker CREs with R package rGREAT (v1.2.1)^147^ (Fisher’ exact test, adjusted *P* cutoff 0.05, BH correction) with all CREs detected for each histone mark as background and visualized it with R package pheatmap (v1.0.12)^167^.

### Epi-partitioned PRSs

We followed a standard PRS pipeline of clumping to build the full PRS model^168^ based on BD GWAS summary statistics. During the quality control step of GWAS summary statistics, we filter SNPs based on their proportions of the presented individuals (call rate ≥ 0.9), minor allele frequency (MAF ≥ 0.01), and imputation quality score (INFO ≥ 0.8). We then carried out clumping by PLINK (v1.9)^166^ with parameters of linkage disequilibrium (LD) R^2^ cutoff (0.4) and distance cutoff (100 kb) based on the LD estimated from the Mayo Clinic cohort. We finally generated PRSs by aggregating effects of all selected SNPs (GWAS *P* < = 0.05 or 0.1, results only shown for *P* < = 0.05) for each genotyped individual by PLINK.

To generate epi-partitioned PRSs, we first collected various CRE sets, including those blood CRE groups showing co-activity patterns across reference epigenomes in our study (Fig. 1b), CRE groups detected in four tissues from our previous eGTEx report^70^, BD differential CREs in blood, blood gCREs, and the blood gCREs sharing BD genetics based on two statistical methods coloc and MR. Next, for each epi-partitioned PRS, we selected the SNPs located near the corresponding CRE set by bedtools v2.26.0 (bedtools window -w 2000) before clumping with the same parameters as the full model.

To evaluate the robustness of our epi-partitioned PRS framework across different PRS construction methods, we applied both a clumping-and-thresholding (C + T) approach and a Bayesian shrinkage model, PRS-CS v1.1.0^169^, using identical sets of CREs. Notably, when visualizing the correlation between C + T-based and PRS-CS-based scores across matched epigenomic partitions (Supplementary Fig. 9b), we found that the strongest correlations were consistently between the same partition (e.g., immune, Broadly active, or gCRE sets) regardless of the method used. We also found that several key results remained robust, including: (1) PRSs partitioned by CRE groups primarily active in the immune system and blood gCREs identified in our blood study continued to differentiate BD patients from controls and (2) PRSs based on gCREs associated with BD via GWAS-QTL colocalization or Mendelian randomization still distinguished patient subgroup 3 and remained correlated with the epigenomic latent factor LF9. This observation supports the robustness of our epi-partitioning strategy in capturing biologically meaningful inter-individual variation.

### Drug repurposing analysis

In this analysis, epigenomic signatures were used as proxies for downstream transcriptional states, under the assumption that disease-associated changes in histone modifications reflect altered gene regulatory programs that can be reversed by pharmacological perturbations, analogous to expression-based drug repurposing frameworks such as CMap. We downloaded 49,488 differential gene expression profiles of 12k genes from hematopoietic lineages in response to 33,627 compounds from CMap (LINCS 2020). We then tested in these profiles the enrichment of gene sets with BD up-/down-regulated epigenomic signatures identified in a previous step (Fig. 2b, c) by Gene Set Enrichment Analysis (GSEA)^100^ implemented in the Python (3.11.5) package gseapy (v1.1.1)^170^ and run with the following parameters: permutation_num=1000, max_size=2000. We only considered drugs/compounds “BD- repurposed” if their corresponding differential expression profiles (1) significantly enriched both BD gene sets (*P* < 0.001, the minimum *P*-value could be achieved with 1000 times of permutations) by GSEA, (2) showed down-regulation of BD up-regulated genes, and (3) showed up-regulation of BD down-regulated genes. We visualized networks with cytoscape v3.10.4^171^. Drug annotation with ATC code in Fig. 6c and Supplementary Fig. 11d, e was downloaded from DrugBank v5.1.12^172^.

### Reporting summary

Further information on research design is available in the Nature Portfolio Reporting Summary linked to this article.

## Supplementary information

Supplementary Information

Description of Additional Supplementary Files

Supplementary Data 1

Supplementary Data 2

Supplementary Data 3

Supplementary Data 4

Supplementary Data 5

Supplementary Data 6

Supplementary Data 7

Reporting Summary

Transparent Peer Review file

## Source data

Source Data

## Data Availability

Raw ChIP-seq data (in BAM format), processed count matrices, genotype matrix, and metadata including sex, age, race, and ethnics are available at GEO under accession number GSE316497. We confirm that no direct identifiers (such as names, exact dates, or contact information) are included in the manuscript or shared datasets. Sex is reported in accordance with the journal’s policy on sex and gender reporting. Individual-level clinical data, including ICD9 codes, are not shared to minimize the risk of re-identification. Genotype data are limited to processed forms of common variants (minor allele frequency >5%), and all shared data have been de-identified in accordance with institutional review board approvals and data-sharing guidelines. Blood hQTL summary statistics across five marks in this study, Bipolar Disorder GWAS summary statistics leaving the Mayo Clinic cohort out, and brain cell type eQTL summary statistics based on sn-RNA-seq data in ROSMAP cohort could be found in Zenodo (https://zenodo.org/records/19404437). Source data are provided with this paper.

## Code availability

All codes for this study were deposited in Github (https://github.com/HouGroup-CompBio-GxE/BD_blood_Epigenome_NatComm_2026), and archived in Zenodo (https://zenodo.org/records/19405608)^173^.
