# Supplementary material for: Genotype epigenome phenotype integration reveals peripheral immune contributions to type I bipolar disorder
Source: Nat Commun. 2026 May 15;17:7740. doi: 10.1038/s41467-026-72543-0 (PMC13434788; doi:10.1038/s41467-026-72543-0)
Supplement: Supplementary file 1 [file 41467_2026_72543_MOESM10_ESM.pdf]

nature portfolio

Corresponding author(s): Manolis Kellis & Tamas Ordog & Lei Hou

Last updated by author(s): 4/3/2026

Reporting Summary

Nature Portfolio wishes to improve the reproducibility of the work that we publish. This form provides structure for consistency and transparency in reporting. For further information on Nature Portfolio policies, see our Editorial Policies and the Editorial Policy Checklist.

Statistics

For all statistical analyses, confirm that the following items are present in the figure legend, table legend, main text, or Methods section.

n/a	Confirmed	
☐	☒	The exact sample size (*n*) for each experimental group/condition, given as a discrete number and unit of measurement
☐	☒	A statement on whether measurements were taken from distinct samples or whether the same sample was measured repeatedly
☐	☒	The statistical test(s) used AND whether they are one- or two-sided<br>*Only common tests should be described solely by name; describe more complex techniques in the Methods section.*
☐	☒	A description of all covariates tested
☐	☒	A description of any assumptions or corrections, such as tests of normality and adjustment for multiple comparisons
☐	☒	A full description of the statistical parameters including central tendency (e.g. means) or other basic estimates (e.g. regression coefficient) AND variation (e.g. standard deviation) or associated estimates of uncertainty (e.g. confidence intervals)
☐	☒	For null hypothesis testing, the test statistic (e.g. *F*, *t*, *r*) with confidence intervals, effect sizes, degrees of freedom and *P* value noted<br>*Give P values as exact values whenever suitable.*
☐	☒	For Bayesian analysis, information on the choice of priors and Markov chain Monte Carlo settings
☐	☒	For hierarchical and complex designs, identification of the appropriate level for tests and full reporting of outcomes
☐	☒	Estimates of effect sizes (e.g. Cohen's *d*, Pearson's *r*), indicating how they were calculated

Our web collection on statistics for biologists contains articles on many of the points above.

Software and code

Policy information about availability of computer code

Data collection	ChIP-seq profiling sequencing data was generated on an Illumina HiSeq 4000 instrument in the Mayo Clinic Center for Individualized Medicine Medical Genomics Facility. Raw sequencing data was aligned and processed using bwa 0.5.9. Whole Genome Sequencing data was generated by Illumina's HiSeq X, fastq files were then channeled through the Mayo Genome GPS v4.0 system. Using the Burrows–Wheeler Aligner, reads were aligned to the human reference sequence (GRCh38 build). The Genome Analysis Toolkit (GATK) was employed for local realignment surrounding indels. Variant identification was executed using the GATK HaplotypeCaller, followed by variant recalibration (VQSR) in line with the GATK's recommended best practices.
Data analysis	Analyses were performed using the ENCODE ChIP-seq pipeline (https://github.com/kundajelab/chipseq_pipeline), bwa (v0.5.9), samtools (v1.3.1), MACS2 (v2.1.1), bigWigAverageOverBed (v2), Homer (v4.11.1), UCSC LiftOver, bedtools (v2.26.0), FastQTL (v2.184), PLINK (v1.9), LDSC (v1.0.0), coloc (v3.2-1), MendelianRandomization (v0.4.1), Cytoscape (v3.10.4), gseapy (v1.1.1), PRS-CS (v1.1.0), and R v3.6.0 or R v4.0 packages including DESeq2 (v_1.20.0), gcapc (v1.4.0), flexclust (v1.4-0), ComplexHeatmap (v2.4.3), annotatr (v1.6.0), rGREAT (v1.2.1), addreg (v3.0), sva (v3.28.0), limma (v3.36.5), meta (v4.9-5), qvalue (v2.12.0), MOFA (v1.6.2), mclust (v5.4.3), and pheatmap (v1.0.12).

For manuscripts utilizing custom algorithms or software that are central to the research but not yet described in published literature, software must be made available to editors and reviewers. We strongly encourage code deposition in a community repository (e.g. GitHub). See the Nature Portfolio guidelines for submitting code & software for further information.

Data

Policy information about availability of data

All manuscripts must include a data availability statement. This statement should provide the following information, where applicable:

- Accession codes, unique identifiers, or web links for publicly available datasets
- A description of any restrictions on data availability
- For clinical datasets or third party data, please ensure that the statement adheres to our policy

De-identified ChIP-seq data are submitted to GEO. Raw ChIP-seq data (in BAM format) , processed count matrices, and metadata including sex, age, race, and ethnics are available at GEO under accession number GSE316497 and genotype data will be later accessible through Zenodo.

Research involving human participants, their data, or biological material

Policy information about studies with human participants or human data. See also policy information about sex, gender (identity/presentation), and sexual orientation and race, ethnicity and racism.

Reporting on sex and gender	We used sex through our manuscript. There are 73 males and 107 female in our cohort. We took sex as a covariate to remove in our differential CRE analysis and hQTL mapping.
Reporting on race, ethnicity, or other socially relevant groupings	Most of the cohort is non-Hispanic whites, ethnic information for 9 of them is not fully available.
Population characteristics	this is a cohort of bipolar I disorder patients (N=88) and age-matched controls (N=92), with median age of 44.5.
Recruitment	Case and control participants are registered in Mayo Clinic Bipolar Disorder Biobank and the Mayo Clinic Biobank respectively. Our IRB protocol excludes controls with diagnosed or likely psychiatric disorders based on a questionnaire covering diagnoses of bipolar disease, depression, Down syndrome, autism, ADHD, and other psychiatric or mental illness, as well as self-reported mood alterations scored as shown below. No potential bias are concerned.
Ethics oversight	This study was reviewed and approved by the Mayo Clinic Institutional Review Board and by the access committees from both Mayo Clinic Bipolar Disorder Biobank and the Mayo Clinic Biobank.

Note that full information on the approval of the study protocol must also be provided in the manuscript.

Field-specific reporting

Please select the one below that is the best fit for your research. If you are not sure, read the appropriate sections before making your selection.

- [x] Life sciences
- [ ] Behavioural & social sciences
- [ ] Ecological, evolutionary & environmental sciences

For a reference copy of the document with all sections, see nature.com/documents/nr-reporting-summary-flat.pdf

Life sciences study design

All studies must disclose on these points even when the disclosure is negative.

Sample size	we include all the samples available.
Data exclusions	ChIP-seq samples are excluded if they did not pass our quality control cretirions.
Replication	Due to limited data availabe, we cannot replicate our findings with existing BD histone modifcation data. Instead, we checked differential signals related to the diseases across histone marks, which shows strong overlap between marks. We also found substantial overlap between candidate genes we inferred and those supported by other evidence such as GWAS-eQTL cololcalization.
Randomization	Participants are separated into two groups based on whether they were diagnosed with bipolar disorder, and two groups are age-matched. There is no need for randomization in the design, since we focused on the differential signal between two groups
Blinding	Blinding was not performed in this study. Case and control samples were derived from distinct cohorts and processed separately, making blinding to group allocation not feasible during data collection. In addition, the primary data generation and analysis involved standardized, automated ChIP-seq and computational pipelines, which are objective and not subject to investigator-dependent bias. Therefore, blinding was not considered necessary for this study.

Reporting for specific materials, systems and methods

We require information from authors about some types of materials, experimental systems and methods used in many studies. Here, indicate whether each material, system or method listed is relevant to your study. If you are not sure if a list item applies to your research, read the appropriate section before selecting a response.

Materials & experimental systems

n/a	Involved in the study
☐	☒ Antibodies
☒	☐ Eukaryotic cell lines
☒	☐ Palaeontology and archaeology
☒	☐ Animals and other organisms
☐	☒ Clinical data
☒	☐ Dual use research of concern
☒	☐ Plants

Methods

n/a	Involved in the study
☐	☒ ChIP-seq
☒	☐ Flow cytometry
☒	☐ MRI-based neuroimaging

Antibodies

Antibodies used

Target Supplier Host Clone ID Isotype Final conc.
H3K27ac CST Rabbit mc 8173 IgG 2µg/4 x 106 cells, https://www.cellsignal.com/products/primary-antibodies/histone-h3-acetyl-lys27-d5e4-xp-rabbit-mab/8173;
H3K4me1 Abcam Rabbit pc ab8895 IgG 2µg/4 x 106 cells
H3K4me1 CST Rabbit mc 5326 IgG 2µg/4 x 106 cells
H3K4me3 EDL Rabbit pc EDL Lot1 IgG 2µg/4 x 106 cells
H3K27me3 CST Rabbit mc 9733 IgG 2µg/4 x 106 cells
H3K36me3 Active Motif Rabbit pc 61101 IgG 2µg/4 x 106 cells

Validation

These commercial antibodies is widely used and has been validated in human for western blotting and IP

Clinical data

Policy information about clinical studies

All manuscripts should comply with the ICMJE guidelines for publication of clinical research and a completed CONSORT checklist must be included with all submissions.

Clinical trial registration

Provide the trial registration number from ClinicalTrials.gov or an equivalent agency.

Study protocol

Note where the full trial protocol can be accessed OR if not available, explain why.

Data collection

Describe the settings and locales of data collection, noting the time periods of recruitment and data collection.

Outcomes

Describe how you pre-defined primary and secondary outcome measures and how you assessed these measures.

Plants

Seed stocks

Report on the source of all seed stocks or other plant material used. If applicable, state the seed stock centre and catalogue number. If plant specimens were collected from the field, describe the collection location, date and sampling procedures.

Novel plant genotypes

Describe the methods by which all novel plant genotypes were produced. This includes those generated by transgenic approaches, gene editing, chemical/radiation-based mutagenesis and hybridization. For transgenic lines, describe the transformation method, the number of independent lines analyzed and the generation upon which experiments were performed. For gene-edited lines, describe the editor used, the endogenous sequence targeted for editing, the targeting guide RNA sequence (if applicable) and how the editor was applied.

Authentication

Describe any authentication procedures for each seed stock used or novel genotype generated. Describe any experiments used to assess the effect of a mutation and, where applicable, how potential secondary effects (e.g. second site T-DNA insertions, mosiacism, off-target gene editing) were examined.

ChIP-seq

Data deposition

☐ Confirm that both raw and final processed data have been deposited in a public database such as GEO.

☒ Confirm that you have deposited or provided access to graph files (e.g. BED files) for the called peaks.

Data access links
May remain private before publication.

Reviewer can get access to the raw data through link here https://dataview.ncbi.nlm.nih.gov/object/PRJNA1400219?reviewer=n3k640uu6b6u5vdj5emk8v64bb, and to GSE316497 with token “ehmfwquqbjkhvuv”.

Files in database submission

Raw ChIP-seq data (in BAM format) , processed count matrices, and metadata including sex, age, race, and ethnics are available at GEO under accession number GSE316497

Genome browser session (e.g. UCSC)

Provide a link to an anonymized genome browser session for "Initial submission" and "Revised version" documents only, to enable peer review. Write "no longer applicable" for "Final submission" documents.

Methodology

Replicates

We do not have biological replicates for each individual.

Sequencing depth

pair-end data, reads length 51bp. Please refer to supplementary Table 1 for the total reads of each of ChIP experiment.

Antibodies

Target Supplier Host Clone ID Isotype Final conc. Validation source
H3K27ac CST Rabbit mc 8173 IgG 2µg/4 x 106 cells, https://www.cellsignal.com/products/primary-antibodies/histone-h3-acetyl-lys27-d5e4-xp-rabbit-mab/8173
H3K4me1 CST Rabbit mc 5326 IgG 2µg/4 x 106 cells, https://www.cellsignal.com/products/primary-antibodies/histone-h3-monmethyl-lys4-d1a9-xp-rabbit-mab/5326
H3K4me3 EDL Rabbit pc EDL Lot1 IgG 2µg/4 x 106 cells, PMID: 29268714
H3K27me3 CST Rabbit mc 9733 IgG 2µg/4 x 106 cells, https://www.cellsignal.com/products/primary-antibodies/histone-h3-trimethyl-lys27-c36b11-rabbit-mab/9733
H3K36me3 Active Motif Rabbit pc 61101 IgG 2µg/4 x 106 cells https://www.activemotif.com/catalog/details/61101/histone-h3-trimethyl-lys36-antibody-pab

Peak calling parameters

macs2 callpeak -t ${REP1_TA_FILE}.tagAlign.gz -f BED -n ${PEAK_OUTPUT_DIR}/${CHIP_TA_PREFIX} -g ${GENOMESIZE} -p 1e-2 --nomodel --shift 0 --extsize ${FRAGLEN} --keep-dup all -B --SPMR

Data quality

Samples were then filtered based on three QC metrics: a) Relative Strand Cross-correlation coefficient (RSC), a key ChIP-seq QC on signal/noise; b) total reads; and c) correlation between the signal of samples and those from blood samples in Roadmap reference epigenome. We define “Tier 1” samples as those RSC score ≥ 2, total reads ≥ 30M, and for which their matched Roadmap tissue was in the top three most correlated Roadmap epigenomes. We defined "Tier 2" samples with relatively permissive cutoffs: RSC score ≥ 1, total reads ≥ 20M, and the same criteria of matched-tissue correlation.

Software

Raw sequencing data was aligned and processed using bwa and picard, followed by AQUAS pipeline (https://github.com/kundajelab/chipseq_pipeline)
