# Supplementary material for: Genotype epigenome phenotype integration reveals peripheral immune contributions to type I bipolar disorder
Source: Nat Commun. 2026 May 15;17:7740. doi: 10.1038/s41467-026-72543-0 (PMC13434788; doi:10.1038/s41467-026-72543-0)
Supplement: Supplementary file 2 [file 41467_2026_72543_MOESM11_ESM.pdf]

nature portfolio

Peer Review File

Genotype Epigenome Phenotype Integration Reveals Peripheral Immune Contributions to Type I Bipolar Disorder

Corresponding Author: Dr Lei Hou

This file contains all reviewer reports in order by version, followed by all author rebuttals in order by version.

Version 0:

Reviewer comments:

Reviewer #1

(Remarks to the Author)
This manuscript analyses 833 genome-wide ChIP-seq dataset from 180 IDs (88 BD patients, 92 controls) for 5 histone modifications in whole blood in order to investigate differential cis regulatory elements (dCRE) in BD cases vs. controls. Using whole genome seq histoneQTLs are generated to identify genetically driven CREs (gCREs). Using GWAS data, colocalization and Mendelian Randomization approaches dCRE and gCRE are linked with genes, revealing important novel, potentially causal immune specific candidates. Combined analyses with drug induced perturbation signatures identify interesting compounds and clustering approaches based on these epigenetic signatures reveal 5 potential subtypes of patients that correlate with disease PRS.

Strengths:
-very rich and comprehensive analyses to validate function
-added also other publicly available datasets
-largest BD histone dataset
-all code is available
-link this to drug targets for possible treatment strategies

Limitations:
Lack of information on clinical and epidemiological features of cases and controls
Lack of information on potential batch effects
largest BD histone dataset but also no replication dataset available
-clustering is based on 88 patients only
-homogenous population (mainly white non-Hispanic) => generalizability to other ethnicities might be limited, especially for the PRS

Minor:
- “gender” - I suppose this means “genetic sex”, please correct throughout the manuscript
- In the figure legends it states type BD but not in the manuscript. I would mention this at least once.
-Figure3 b: add GWAS abbreviations to the legend
-Figure 3 d: unclear what “rest” means

Detailed comments:

Intro
- page 2, line 33: “for five histone modifications”: I would once name these here

- page 2, line 39 “802-22k dCREs per histone mark”: add average number of dCREs across all five histone marks and state for which modification you found the lowest and for which the highest amount of dCREs

Results

An epigenomic atlas of 439k blood CREs across 180 individuals
-page 3, line 21: please include more information on the 180 individuals in a demographic table including sex, age, medication and type of BD (if applicable). The fact that the population was mainly white and non-Hispanic ensures homogeneity which might be especially important for the PRS but could also lead to reduced generalizability to other populations. This should be noted in the discussion later.

Differential activity of CREs in bipolar disorder
-page 4, line 21: even though different across individuals, estimated cell type proportions look quite comparable across BD cases and controls. Where there any significant differences between these groups?

-extended figure 2: while the majority is explained by celltype proportions, a small amount of variance in the SVA is explained by "gapapentin_current" -> any comment on this? Furthermore, the proportion of variance explained by neutrophils in the figure seems to be negative. How significant is the SVA, why was only one chosen?

-page 4, line 28: How do the overall numbers of detected dCREs relate to detected CREs in general? Do you find more dCREs for those marks that are more frequent in general?

-page 4, line 32: GO: how was the background defined?

-page 5, line 4: it would be interesting to evaluate if hQTLs differ between BD cases and controls, at least in their effect direction. This could indicate that the genetic regulation is mediated by disease.

-page 5, line 8: should this be Figure 2e?

-page 5, line 19: unclear why you exactly look into lower back pain.

-page 5, line 30: why did you not control for genetic PCs in the hQTL analysis?

-page 6, line 5: You could also include GWAS on cross-disorder psychiatric traits as well as immune markers (on top of immune-related diseases themselves). You later state that MDD is closely related to BD (page 6, line 32) – why was no GWAS on MDD included?
Did you add a negative control GWAs for which no immune signal and no association with psychiatric disorder should be expected, e.g. height? This could also be interesting later on for the colocalization analysis.

Heterogeneity of CRE activities in BD patients reveals new phenotypic subtypes
-page 8, line 28: I think it is worth mentioning that L1 is correlated with lithium which is a therapy for BD, even though this correlation is not significant after correction. How high was the correlation and the nominal p-value. How many patients were treated with lithium?

-page 8, line 40 onwards: it should be noted here that patient subclusters are quite low in sample size. Furthermore, would you find similar enrichments if you tested all individuals per cluster or only controls or are the enrichments specific to the cases? Low N per cluster could also relate to non-significance of some relevant correlations, such as for example with lithium

-page 9, line 14: the PRS analysis needs more detail, please include the number of SNPs per PRS as well as the correlation between the different PRS.

-page9, line 28: I think it is noteworthy that all PRS significantly different between cases and controls are either based on overlap with blood, multi-tissue or placenta data. This is quite interesting given that placental biology has been linked to psychiatric disorders.

Discussion
-page 11, line 36: as you note BD has been associated with elevated immune markers, did you also find this in your dataset based on the EHR data?

-page 13, line 44: limitations: even though the largest sample, it is still a limited number of BD patients, mainly white non-Hispanic. This could influence generalizability, especially of the PRS and hQTLs. Furthermore, no replication dataset is available.

Are any comparable datasets for other psychiatric disorders available which could be used to at least compare the number of identified dCREs?

Methods

Sample description.
Rich data on cases and controls are available. A table summarizing potential confounds, including medication, psychiatric comorbidity, somatic disease, smoking would be very helpful. Also could the PRS for BD be shown for cases vs. control.
It appears that cases and controls were collected from different biobanks– information on comparability of collection

procedures and storage are lacking so that batch effects cannot be excluded.

ChIP Seq:
More detail is needed on how samples were randomized and processed for ChIP-Seq and how possible batch-effects were dealt with.
Also were Tier 1 and Tier 2 samples equally distributed between cases and controls?

WGS
-page 19, line 13: reads were aligned based on hg38, however later it is stated that ChIPSeq peaks were aligned based on hg19. Was any up/downlifting performed to map these annotations?

Driver genes priorization
-page 23, line 13: what does "worked significantly better" exactly mean?

Epi-partitioned PRS
-page 24, line 1: more information on the construction of PRS is needed. Which program was used? From the reference, it seems that you used PRSice which is based on clumping and thresholding. If so, which p-value threshold was chosen? Usually, using all SNPs and correction for LD between them performs better than clumping and thresholding, e.g. using PRScs or LDpred.

(Remarks on code availability)
NA

Reviewer #2

(Remarks to the Author)
I co-reviewed this manuscript with one of the reviewers who provided the listed reports. This is part of the Nature Communications initiative to facilitate training in peer review and to provide appropriate recognition for Early Career Researchers who co-review manuscripts.

(Remarks on code availability)

Reviewer #3

(Remarks to the Author)
Hou et al. present a very interesting manuscript exploring the immune-psychiatry axis in bipolar disorder through the generation and integrative analysis of genomic, epigenomic and health record data. The dataset will be a valuable resource for the research community and the results offer intriguing insights for further analysis.

Please find comments listed below:

• For readability, needs a clear definition in introduction of what a "CRE" vs a "candidate CRE" is and how they differ from a histone peak. Furthermore, how do you know the regulatory elements act in cis?

• Heterogeneity in histone peaks within the cohort has been investigated. However, it is unclear how many of the 800k+ CREs are common across subjects within control and bipolar groups. These data would simply illustrate inter-individual variability for histone peaks.

• Relating to Figure 1b, the results section would benefit from a brief description of the method for clustering profiles into 84 modules and 13 groups. Also, in the figure legend for this figure, it seems the axis labels are in opposite orientation. 242 reference epigenomes are columns (bottom) and 84 modules are rows (left).

• [Page 4, Line 36] "These results confirm an elevated level of inflammation and reduced adaptive immune response in BD" – these results support this notion but are not confirmatory, I suggest rewording this statement to reflect this.

• [Page 4, Line 45] "15 were associated with BD, 46 based on GWAS signals, differential signals, or mechanistic evidence" – mechanistic evidence requires supporting citations to be added to Supp. Table S3 or in text.

• [Page 5, Line 29] Define "peer factors" in explanation of hQTL analysis.

• [Page 5, Line 33] It is unclear how the millions of hQTLs map to thousands of CREs, I suggest further explanation around this process. Are these hQTLs loci or specific SNPs with significant associations.

• [Page 5, Line 43] "We also identified 4k gCREs shared between H3K27ac and H3K4me1, and 1k gCREs shared between H3K27me3 and H3K4me1 (Fig. 3a)." – how is sharing defined? Much of authors' interpretation depends on sharing or not sharing; needs to be clear early on in text how this is defined. Would you need to do coloc to confirm these are shared?

• Results section generally light on the how analysis was approached, e.g. deconvolution described as 'our method', brief description within results text would be useful.

• Clustering needs some analysis of stability to participant inclusion to be convincing (e.g. subsampling).

• Why look for LDSC enrichment in this smaller dataset of groups within dataset rather than in whole of EpiMap? How do the G groups relate to EpiMap groups?

• In relation to the clustering of participants into 5 groups, dCREs which are differential elements between cases and controls were used for MOFA to identify latent factors, yet cases and controls were poorly discriminated in the group clustering, it would be useful to add discussion on why this may be the case.

• Following this clustering, enrichment of EHR terms was then performed exclusively on cases within these groups, yet the controls contributed to the grouping. How does this enrichment result change with/without the controls included?

• The authors found that genes near BD GWAS-colocalizing gCREs related to ER function, potentially implicating ER function in BD pathogenesis. How do the pathways highlighted by coloc with hQTLs differ from pathways highlighted by coloc with eQTLs? In a direct comparison, how correlated is pathway enrichment by blood hQTL with enrichment by blood eQTL? It's of significant interest to understand how the information from different omic layers differs.

• [Page 9, Line 43] It is unclear how the medication GO enrichment was performed to derive the 6 immune related medications described, further clarification required.

• In relation to the medication repurposing analysis, these medications largely act on proteins, yet the analysis is mostly based on epigenomic profiles. This inference limitation should be discussed. In addition, this analysis could be expanded to include the mode of action of these medications, are any of them modifiers of the epigenome or transcriptional machinery?

• Was there evidence that in patients in their cohort, antipsychotics reversed the BD GWAS-colocalizing gCREs or dCREs?

• Can the authors highlight the drugs prioritised as most likely to worsen BD? Discussion of flutamide exacerbating mania in discussion, but CMap-prioritised drugs predicted to worsen BD would be good to highlight in results.

• Extended Data Figure 2d – within the network the edge width looks to represent a data feature, but this is not defined.

(Remarks on code availability)

Reviewer #4

(Remarks to the Author)
I co-reviewed this manuscript with one of the reviewers who provided the listed reports. This is part of the Nature Communications initiative to facilitate training in peer review and to provide appropriate recognition for Early Career Researchers who co-review manuscripts.

(Remarks on code availability)

Reviewer #5

(Remarks to the Author)
The study is well-designed, and the manuscript is well-crafted. Authors profiled histone modification in Type I bipolar disorder patients and controls and mapped hQTLs. By integrating dCREs, gCREs, and CRE–gene links, they identified disease driver genes and grouped cases by epigenomic profiles. Additionally, they discussed the potential for drug repurposing based on expression signatures in the Connectivity Map. Throughout their analyses, they revealed the important role of immune related genes and pathways in disease pathogenesis. The limitations of this study were thoroughly discussed, particularly the lack of paired brain samples and gene expression data.

I have the following minor comments:
1. The study cohort includes 88 Type I BD patients, not Type II BD. Type I and Type II BD are different in terms of genetics, epigenetics and clinical manifestations. I suggest authors to tone done – state that the findings only apply to Type I BD, not BD.

2. Italic gene names, e.g. page 1, line 32, ORMDL3; page 5, line 20-26.

3. Page 2, line 8, 9: please cite the most recent GWAS:
depression: https://www.cell.com/cell/fulltext/S0092-8674(24)01415-6;
bipolar: https://doi.org/10.1038/s41586-024-08468-9

4. Results – section 1: Authors discussed TF motif and GO enrichment analyses results for G3 and G12, but did not mention other groups. Even if the results were not promising, I suggest to briefly discuss, instead of cherry picking the positive ones.

5. Typo: page 4, line 29: adjust P 0.05.

6. Authors set PP4>0.5 as the cutoff in coloc analysis (page 6, line 20; page 7, line 38). This is too liberal. Usually, PP4>0.7 or 0.8 is used.

7. Page 6, line 23: P<= 1e-6 was used for GWAS genome-wide significance cutoff. Is there any reason why not use P<5e-8, which is widely accepted in the field?
8. Figure legend - figure 3b: What do the red box and black box indicate? What's the significance cutoff? It was mentioned in the main text, but not in the figure legend.

9. Please double check paragraph format throughout the manuscript upon publication, e.g. spacing, especially in the method section.

10. Figure 4b: background (grey) bars are missing in the figure, and figure 4b was not discussed in the main text.

11. Figure 4d: blood-specific and brain-blood shared coloc signals were plotted. I am wondering if there is any brain-specific coloc signal.

(Remarks on code availability)

Version 1:

Reviewer comments:

Reviewer #1

(Remarks to the Author)
The authors have addressed all remaining concerns

(Remarks on code availability)

Reviewer #2

(Remarks to the Author)
I co-reviewed this manuscript with one of the reviewers who provided the listed reports. This is part of the Nature Communications initiative to facilitate training in peer review and to provide appropriate recognition for Early Career Researchers who co-review manuscripts.

(Remarks on code availability)
I co-reviewed this manuscript with one of the reviewers who provided the listed reports. This is part of the Nature Communications initiative to facilitate training in peer review and to provide appropriate recognition for Early Career Researchers who co-review manuscripts.

Reviewer #3

(Remarks to the Author)
We thank the authors for their comprehensive responses to our queries. The revised paper is in our view strong and of significant novelty and interest. Most of our concerns are fully addressed.

Two outstanding issues:

Batch effects - cases/controls are largely in different batches as the authors describe. Their rebuttal attempts to address this with permutation testing (but this is more a reflection of whether this signal is just random noise not intrinsic technical variation) and then differential testing within group vs between groups. From our understanding, since cases/controls were batched separately this could be highlighting batch effects not case-control differences. The permutation testing would seem to confirm that not all of the signal is driven by batch. The authors then perform PCA and label based on batch, but this plot is not shown. Please can this plot be shown, and can the authors ensure the text reflects the degree to which the effects may be in part driven by batch.

Inter-individual variability – the authors now show peak presence across samples for H3K27ac (Response Fig 10). Please can the authors add histograms separated by case/control status and include all 5 marks as supplementary figures. We think this variability is important to report so readers can assess which marks are less variable between individuals within group.

(Remarks on code availability)

Reviewer #4

(Remarks to the Author)
I co-reviewed this manuscript with one of the reviewers who provided the listed reports. This is part of the Nature Communications initiative to facilitate training in peer review and to provide appropriate recognition for Early Career Researchers who co-review manuscripts.

(Remarks on code availability)

Reviewer #5

(Remarks to the Author)
Thanks for the authors to anwser and reply my comments and questions. They are fully addressed.

(Remarks on code availability)

Open Access This Peer Review File is licensed under a Creative Commons Attribution 4.0 International License, which permits use, sharing, adaptation, distribution and reproduction in any medium or format, as long as you give appropriate credit to the original author(s) and the source, provide a link to the Creative Commons license, and indicate if changes were made.
In cases where reviewers are anonymous, credit should be given to 'Anonymous Referee' and the source.
The images or other third party material in this Peer Review File are included in the article's Creative Commons license, unless indicated otherwise in a credit line to the material. If material is not included in the article's Creative Commons license and your intended use is not permitted by statutory regulation or exceeds the permitted use, you will need to obtain permission directly from the copyright holder.
To view a copy of this license, visit https://creativecommons.org/licenses/by/4.0/

REVIEWER COMMENTS

Reviewer #1 (Remarks to the Author):

This manuscript analyses 833 genome-wide ChIP-seq dataset from 180 IDs (88 BD patients, 92 controls) for 5 histone modifications in whole blood in order to investigate differential cis regulatory elements (dCRE) in BD cases vs. controls. Using whole genome seq histoneQTLs are generated to identify genetically driven CREs (gCREs). Using GWAS data, colocalization and Mendelian Randomization approaches dCRE and gCRE are linked with genes, revealing important novel, potentially causal immune specific candidates. Combined analyses with drug induced perturbation signatures identify interesting compounds and clustering approaches based on these epigenetic signatures reveal 5 potential subtypes of patients that correlate with disease PRS.

Strengths:
-very rich and comprehensive analyses to validate function
-added also other publicly available datasets
-largest BD histone dataset
-all code is available
-link this to drug targets for possible treatment strategies

We appreciate the reviewers' positive comments.

Limitations:
Lack of information on clinical and epidemiological features of cases and controls
Lack of information on potential batch effects
largest BD histone dataset but also no replication dataset available
-clustering is based on 88 patients only
-homogenous population (mainly white non-Hispanic) => generalizability to other ethnicities might be limited, especially for the PRS

We agree with the reviewers' comments about limitations and we have summarized our response to each of them as follows:

1. EHR/clinical records are subject to privacy considerations. We are currently awaiting guidance from the committee on the next steps and will keep you updated.
2. Thank you for your comment about potential batch effects. Indeed, over 1000 ChIP-seq experiments were performed in 62 mini-batches during 1.5 years. This was necessitated by logistic issues related to sample accessioning, the labor-intensive nature of the epigenomic analyses, and the very limited scalability of these assays. Nevertheless, we conducted comprehensive assessments of batch effects and found that, even when present, they had a limited impact on downstream differential analyses. In all, the small variability in quality reflected

the robust nature of the lab's standard operating procedures, which sufficiently mitigated the risk of significant batch effects.

3. So far as we know, there are no other blood histone modification studies for BD, and developing an independent validation cohort and replicate the 5 ChIP-seq studies and whole-genome sequencing was well beyond the scope of our study and the resources available. Therefore, we have provided a comparison among histone marks as in Suppmentary Fig.2e as a surrogate for replication.

4. Since our main focus was on evaluating patient heterogeneity, we were compelled to limit clustering of data from the 88 patients in most downstream analyses. However, we clustered all samples when it was necessary, for example, in Fig. 5a.We also compared EHR enrichment in clusters of all donors and only patients as dictated by the specific analysis.

5. The patients included in the study reflect the demographics of the BD patient population seen at Mayo Clinic in Rochester, Minnesota. We agree that having our cohort from a single center limits our ability to generalize our conclusion to other ethnicities, especially in relation to PRS. We have updated our discussion to acknowledge this fact.

Please refer to the detailed responses under each specific point below.

Minor:

- "gender" - I suppose this means "genetic sex", please correct throughout the manuscript

Thanks for the comment! It is genetically confirmed and we have replaced "gender" with "sex".

- In the figure legends it states type BD but not in the manuscript. I would mention this at least once.

Very important comment. We have now mentioned it in the introduction, the first sentence in the results, and the first sentence in the methods under "Mayo Clinic participants".

-Figure3 b: add GWAS abbreviations to the legend

Thanks for the comment. We have added the full spelling of GWAS to the legend in Fig3b.

-Figure 3 d: unclear what "rest" means

Thanks for the comment. We have changed it to "Non-hQTL genetic variants."

Detailed comments:

Intro

- page 2, line 33: "for five histone modifications": I would once name these here

Thanks for the comment. We have added "namely, H3K27ac, H3K4me1, H3K4me3, H3K36me3, and H3K27me3"

- page 2, line 39 "802-22k dCREs per histone mark": add average number of dCREs across all five histone marks and state for which modification you found the lowest and for which the highest amount of dCREs

That's a much clearer way to present the results! We have changed it to "We first identified on average 8.4k *cis*-regulatory elements (CREs) defined as peaks in ChIP-seq profiles, across the five histone marks (range: 802 for H3K4me1, and 22k for H3K27ac)"

Results

An epigenomic atlas of 439k blood CREs across 180 individuals

-page 3, line 21: please include more information on the 180 individuals in a demographic table including sex, age, medication and type of BD (if applicable). The fact that the population was mainly white and non-Hispanic ensures homogeneity which might be especially important for the PRS but could also lead to reduced generalizability to other populations. This should be noted in the discussion later.

We included metadata (age, sex, race) in the supplementary table S1, as well as GEO under accession GSE316497 now (Reviewer can get access to the raw data through link here https://dataview.ncbi.nlm.nih.gov/object/PRJNA1400219?reviewer=n3k640uu6b6u5vdj5emk8v64bb, and to GSE316497 with token "ehmfwquqbjkhvuv"). We have clearly mentioned it in the main text, and all the patients are Type I BD.

Genotype data and EHR/clinical records are subject to privacy considerations. We have obtained approval to publicly release genotype matrices for common variants (minor allele frequency >5%). Guidance regarding access and submission of EHR and clinical data is currently under review by the relevant committee. We intend to submit both data types together, potentially to GEO or another approved repository, and will provide updates once a decision has been reached.

We have added "Finally, our study cohort consists primarily of non-Hispanic White individuals diagnosed with Type I bipolar disorder. While this enhances the accuracy of PRS-based analyses and reduces heterogeneity within the cohort, it also limits the generalizability of our findings to other ancestral populations and to individuals with type II BD." to the limitation part of the discussion.

Differential activity of CREs in bipolar disorder

-page 4, line 21: even though different across individuals, estimated cell type proportions look quite comparable across BD cases and controls. Were there any significant differences between these groups?

Important comments! We have tested the difference with linear regression and found there were significant changes in the cell proportions, including lower $CD4^{+}$ T cell (P=0.0036) and NK cell (P=0.05), and increased neutrophil fractions (P=0.0002) in BD. Together with the results of the inhibition of the innate immune system (e.g., CD4+ T cells) and activation of the adaptive system (e.g., neutrophils) based on differential peak analysis after controlling cell fracting, this suggests that in BD, not only is the cell fraction changing, but the epigenomic marks are also likely to change in the corresponding cell types, given the fact that cell fractions were already corrected for by SVA.

-extended figure 2: while the majority is explained by celltype proportions, a small amount of variance in the SVA is explained by "gapapentin_current" -> any comment on this? Furthermore, the proportion of variance explained by neutrophils in the figure seems to be negative. How significant is the SVA, why was only one chosen?

Thanks for checking our results in great detail! The weak association between the SV and "gapapentin_current" suggests that the current medicine taken may have impacted the

epigenome of BD patients. That's why we carried on the differential analysis associated with the medicine, presented in Fig. 6A. Consistently, gabapentin did impact histone marks near genes, enriching terms such as neutrophil-mediated immunity.

Thanks for identifying the negative proportion of variance explained by neutrophils in Supplementary Fig 2b! We checked it, and it was due to a coding bug, which we have corrected in the revised manuscript.

The number of surrogate variables (SVs) included in our model was determined using the num.sv() function from the sva R package[1,2], with the "leek" method. This method estimates the number of significant latent factors by applying an asymptotic conditional singular value decomposition approach[3]. Specifically, it computes the eigenvalues of the residual matrix after removing the effects of known covariates and compares them to an empirically derived null distribution. The "leek" method does not use a fixed significance threshold (such as α = 0.1 in the "be" permutation-based method in the same function), but instead identifies a stable window of rank estimates across multiple subsampling scales and uses a data-driven heuristic to infer the number of SVs. The final estimate is the mode of significant components consistently observed across resampled data. In our data, this approach identified only one surrogate variable as stable and significant for H3K27ac.

-page 4, line 28: How do the overall numbers of detected dCREs relate to detected CREs in general? Do you find more dCREs for those marks that are more frequent in general?
Interesting comment. Not necessarily. Together, we identified 22k dCREs out of 157k H3K27ac CREs, 805 out of 212k for H3K4me1, 1.6k out of 58k for H3K4me3, 16.1k out of 161k H3K36me3, 1.6k out of 248k H3K27me3, suggesting histone mark-dependent impact of BD on the epigenome.

-page 4, line 32: GO: how was the background defined?
We have added "Enrichment of nearby genes for dCRE is calculated by the R package rGREAT (v1.2.1).[4] All CREs detected for each histone mark served as background (**Fig. 2A**)." in the Methods under "Differential CRE analysis and motif enrichment". We have also added similar sentences where the background information was missing.

-page 5, line 4: it would be interesting to evaluate if hQTLs differ between BD cases and controls, at least in their effect direction. This could indicate that the genetic regulation is mediated by disease.
We agree that this is an interesting direction. However, our primary goal in this study is to understand the genetic regulation of histone modifications that are either shared between brain and blood or specific to blood, with a focus on identifying mechanisms contributing to bipolar disorder (BD) risk. While BD may modulate the effect sizes of some hQTLs, potentially revealing how BD influences other disease risks, testing for interaction effects between genotype and disease status falls beyond the scope of our current study.

Nevertheless, the genotype and histone modification data we generated provide a valuable resource for future efforts to explore such disease-modulated regulatory effects. We have now added a sentence in the Discussion to acknowledge this potential and propose it as

an avenue for future research. We have now added one sentence to the limitation part of the discussion — “Third, although our hQTL analysis captured genetic effects across the full cohort, hQTL effect sizes may differ between BD cases and controls, offering insights into how BD may alter genetic risk for other diseases. This represents an important future direction enabled by the genotype and epigenomic data we provide.”

-page 5, line 8: should this be Figure 2e?
We have double checked and verified it is the right panel called. To avoid any misunderstanding, we have simplified this sentence as follows: “[...] two CRE–gene mapping strategies as illustrated in **Fig. 2b (Methods**) ”.

-page 5, line 19: unclear why you exactly look into lower back pain.
Important comment! It was initially used to validate the epigenomic signatures. We agree it is less relevant, and we have removed this part of the results.

-page 5, line 30: why did you not control for genetic PCs in the hQTL analysis?
Thank you for this important comment. We evaluated the potential impact of population stratification on our hQTL analysis in two ways.

First, we performed PCA on the genotype matrix of our cohort. As shown below in the left panel, there is only minimal genetic structure, with the top two PCs explaining only 0.77% and 0.66% of the variance, respectively.

Second, we explicitly assessed the effect of including genetic PCs in the hQTL mapping. For each CRE, we compared the effect sizes (β) and Z-scores of top hQTLs (filtered by a nominal P-value cutoff of 0.001) between models without genetic PCs and models including the top five PCs as covariates. As shown in the middle (effect size) and right (Z-score) panels (H3K27ac shown as an example), the results from the two analyses were nearly identical, with Pearson correlation coefficients exceeding 0.999 (**Response Fig. 1**).

Together, these results indicate that population stratification is minimal in our cohort and that inclusion of genetic PCs is not likely to substantially impact hQTL effect estimates. Therefore, genetic PCs were not included in the final hQTL analysis. We have added the following clarification to the Methods section under hQTL mapping: “To assess the impact of population structure, we performed principal component analysis (PCA) on the genotype matrix and found minimal genetic variation (top two PCs explained <1.5% of variance). Inclusion of the top five genetic PCs as covariates in hQTL mapping yielded nearly identical effect sizes and test statistics (Pearson r > 0.999), indicating that population stratification had minimal impact. Therefore, genetic PCs were not included in the final hQTL analysis”.

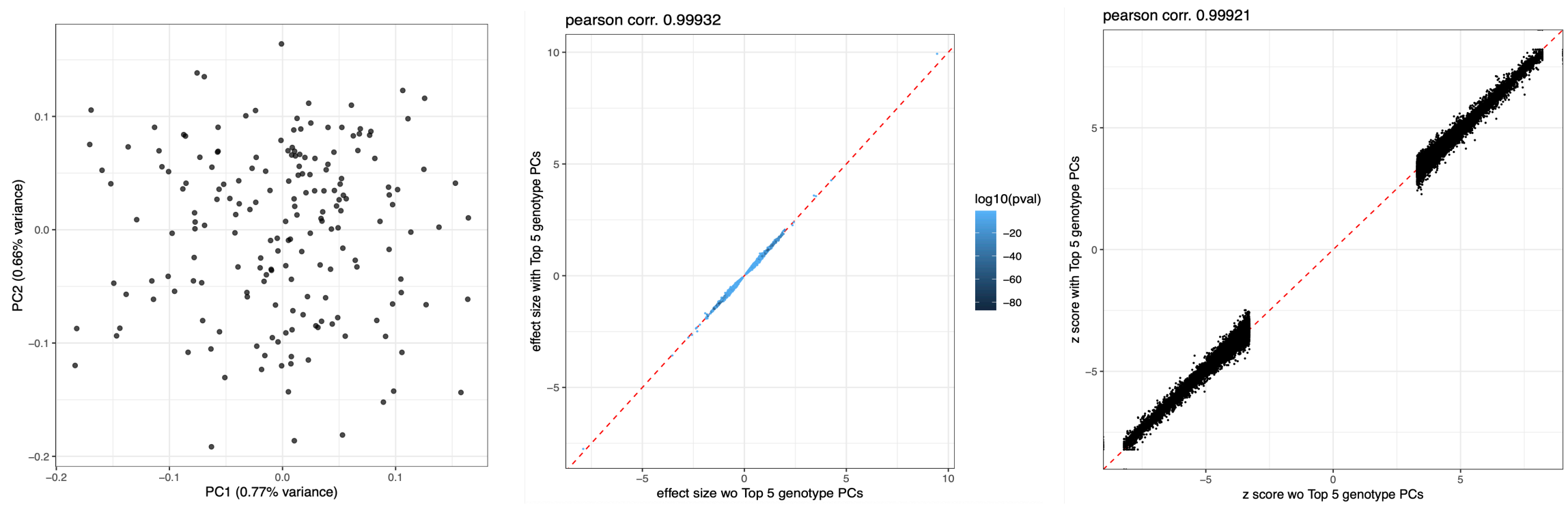

Response Fig. 1. Effect of Top PCs in hQTL analysis.

-page 6, line 5: You could also include GWAS on cross-disorder psychiatric traits as well as immune markers (on top of immune-related diseases themselves). You later state that MDD is closely related to BD (page 6, line 32) – why was no GWAS on MDD included?
Did you add a negative control GWAs for which no immune signal and no association with psychiatric disorder should be expected, e.g. height? This could also be interesting later on for the colocalization analysis.

We appreciate this insightful suggestion. In response, we have extended our stratified LD score regression (LDSC) analysis to include additional traits: immune markers (neutrophil counts and C-reactive protein [CRP]), major depressive disorder (MDD), a cross-disorder psychiatric GWAS meta-analysis, and height as a negative control. The updated results are shown below, with newly added traits indicated by red arrows (**Response Fig. 2**).

Consistent with expectations, neutrophil counts showed strong enrichment in the G3 (blood/immune) CRE group, similar to other immune-related traits such as MS and RA. Interestingly, CRP was enriched in G2 (multi-tissue) and G1 (broadly active) CRE groups, suggesting a regulatory architecture involving broadly active enhancer programs rather than immune-cell-specific ones. Both traits also showed enrichment in H3K27ac and H3K4me1 gCREs in blood, reinforcing the role of enhancer regulation in shaping their genetic architecture.

For psychiatric traits, MDD exhibited an enrichment pattern similar to BD, SCZ, and neuroticism, with a significant signal observed in G12, a group of blood CREs also active in brain and neuronal contexts. In contrast, the cross-disorder GWAS meta-analysis showed a distinct enrichment profile, suggesting that genetic signals shared broadly across psychiatric disorders may not fully capture the immune components revealed in individual disorder analyses. As a specificity control, height showed no significant enrichment in either immune-specific or immune-brain shared CRE groups, reinforcing the biological relevance and context specificity of the observed enrichments.

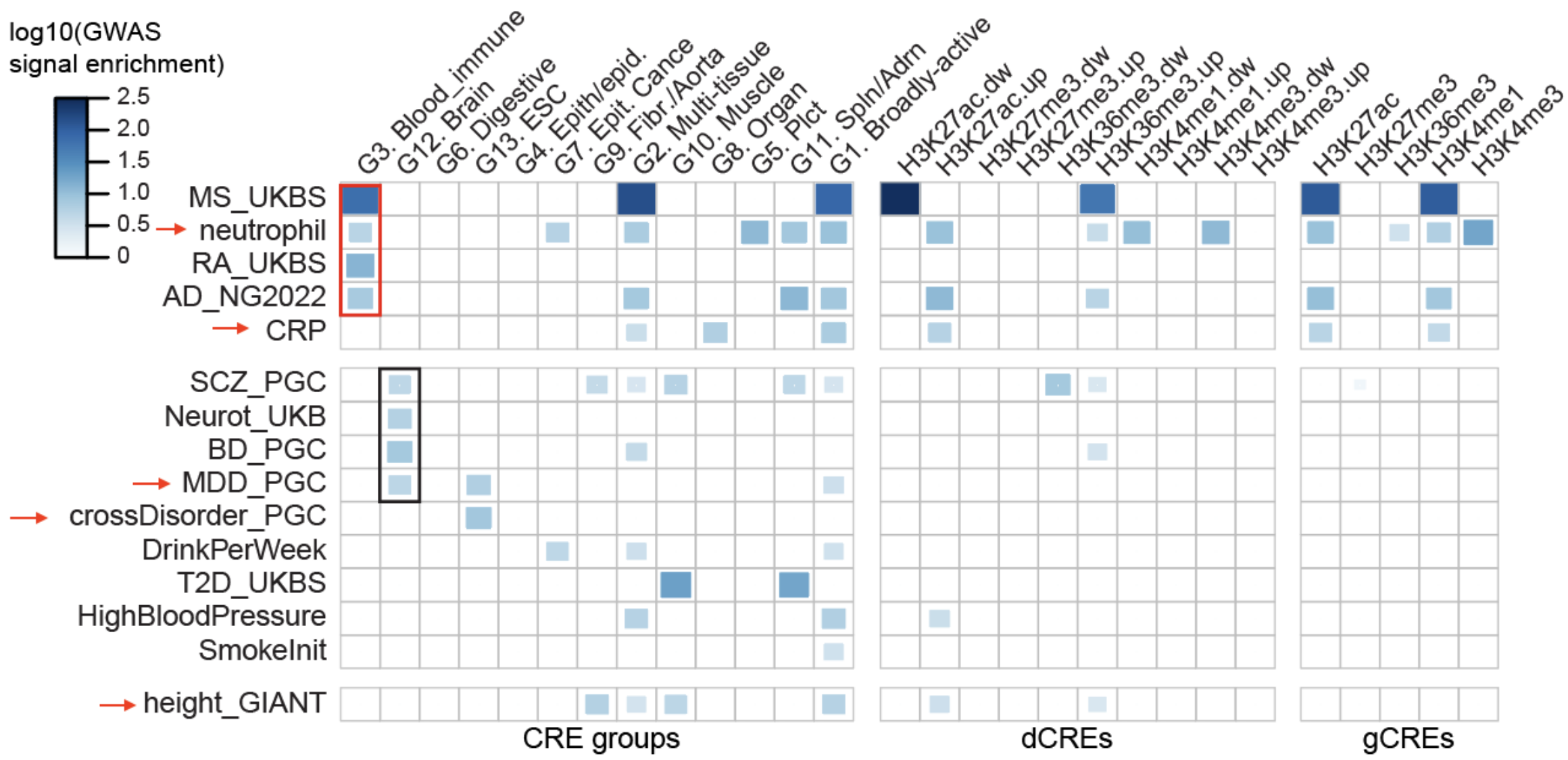

Response Fig. 2. GWAS enrichment including traits newly added.

Taken together, these results imply that, unlike classical immune-related traits or neurodegenerative diseases such as Alzheimer's, psychiatric disorders may engage immune components through regulatory programs that are active in both the brain and the peripheral immune system, rather than through immune-cell-specific regulatory circuits.

We have updated Fig. 3b and Supplementary Fig. 3e, and revised the main text accordingly. In the Discussion, we now include: "We highlighted distinct enrichment patterns between immune-related traits (e.g., autoimmune diseases, Alzheimer's disease, and immune markers) and psychiatric disorders (e.g., BD, SCZ, and MDD). While the former show strong enrichment in immune-specific regulatory elements, psychiatric risk variants appear to impact enhancer programs that are active across both brain and circulating immune cells, pointing to a more systemic and less lineage-specific immune involvement."

Heterogeneity of CRE activities in BD patients reveals new phenotypic subtypes
-page 8, line 28: I think it is worth mentioning that L1 is correlated with lithium which is a therapy for BD, even though this correlation is not significant after correction. How high was the correlation and the nominal p-value. How many patients were treated with lithium?
Thanks for carefully reading our results! The R^2 for LF1 and lithium total score is 0.34 with P-value 0.03. There are 33 patients with current lithium treatment. Instead of LF1, we highlighted LF8 and LF15, which were significantly correlated with lithium, with adjusted P-value<=0.05. We have also identified lithium-associated dCREs and found that they were enriched for processes related to telomere and DNA repair, consistent with previous knowledge, mentioned in the discussion and tab "enrichment for drug epi (2)" of **Supplementary Table 6.** These results suggest current BD medicine may impact the blood epigenomic profiles.

We have now added "LF8 and LF15 were correlated with lithium total score, reflecting cumulative lithium exposure in BD treatment" in the results section.

-page 8, line 40 onwards: it should be noted here that patient subclusters are quite low in sample size. Furthermore, would you find similar enrichments if you tested all individuals per cluster or only controls or are the enrichments specific to the cases? Low N per cluster could also relate to non-significance of some relevant correlations, such as for example with lithium

It is a valid concern. Due to the limited number of patients in our cohort, it is insufficient to reveal all the potential associations, for example, between lithium and subtypes.

We performed EHR enrichment across the full cohort (patients + controls), grouped by the same clustering. The overall patterns were consistent with the patient-only results shown in Fig. 5c: Groups 1 and 2 remained enriched for infection- and inflammation-related terms, group 3 for impaired glucose tolerance, and group 4 for quetiapine usage and hypertension. Some additional terms emerged, such as substance use–related annotations (e.g., nicotine and cannabis) in group 1 (**Response Fig. 3**). These results further support the link between blood epigenomic profiles and physiological states.

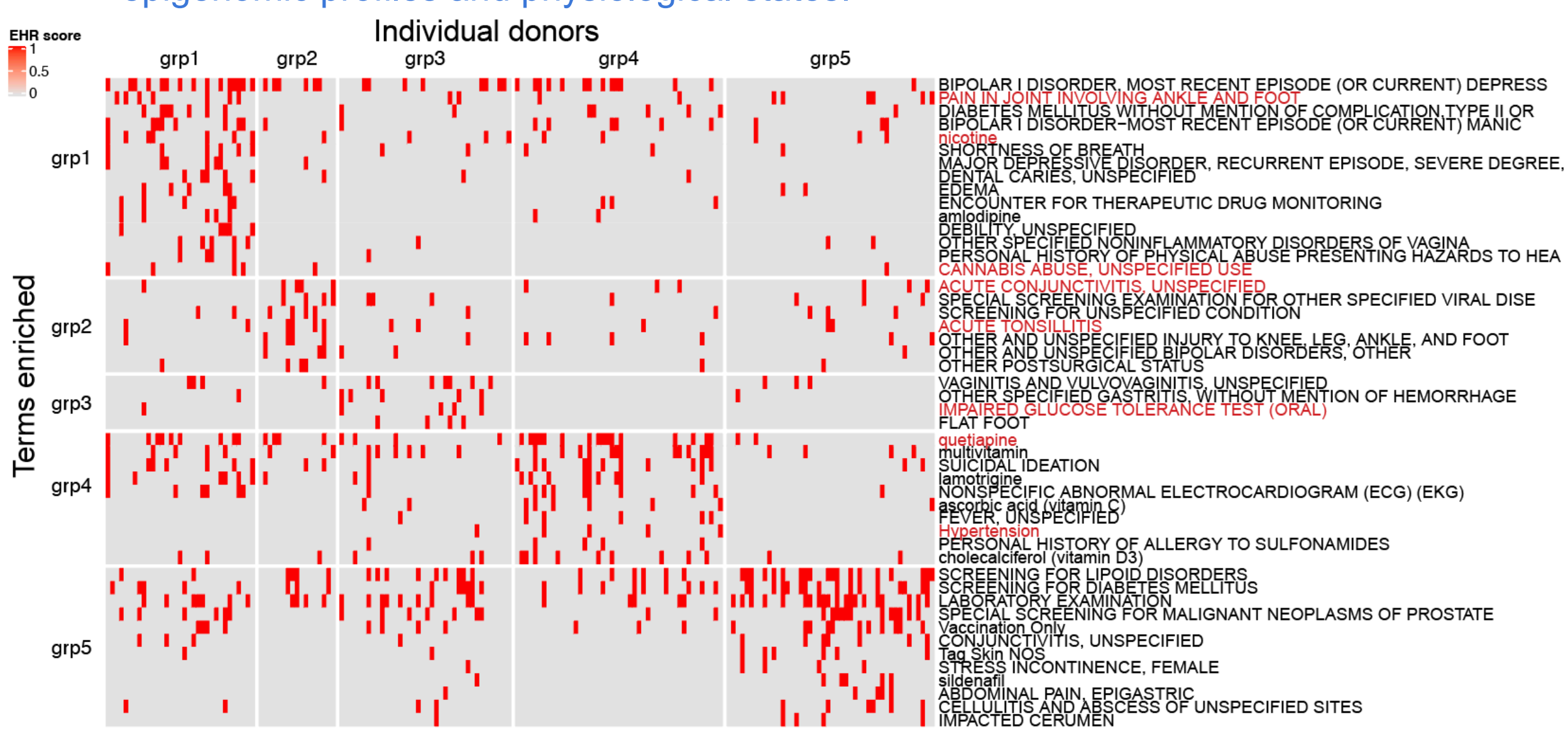

Response Fig. 3. Enrichment of EHR terms for subgroups of all donors

However, including controls in the enrichment analysis introduces interpretational challenges, as the proportion of controls varies across groups. This imbalance can bias enrichment results and confound group-specific signals. We decided to only include results for patient clusters only. We have included a statement about the limitation of sample size to reveal other subgroups (potentially due to treatment such as lithium) in the discussion as follows: "Fifth, our patient subtyping and enrichment analyses are limited by small sample sizes in certain subclusters, reducing statistical power to detect associations with clinical traits or treatment exposures, such as lithium, that may drive meaningful subgroups. Larger cohorts will be required to robustly capture these effects."

-page 9, line 14: the PRS analysis needs more detail, please include the number of SNPs per PRS as well as the correlation between the different PRS.

Thanks for your comment! We have now included the number of SNPs and the correlation among PRS in the Supplementary Fig. 5f (**Response Fig. 4**).

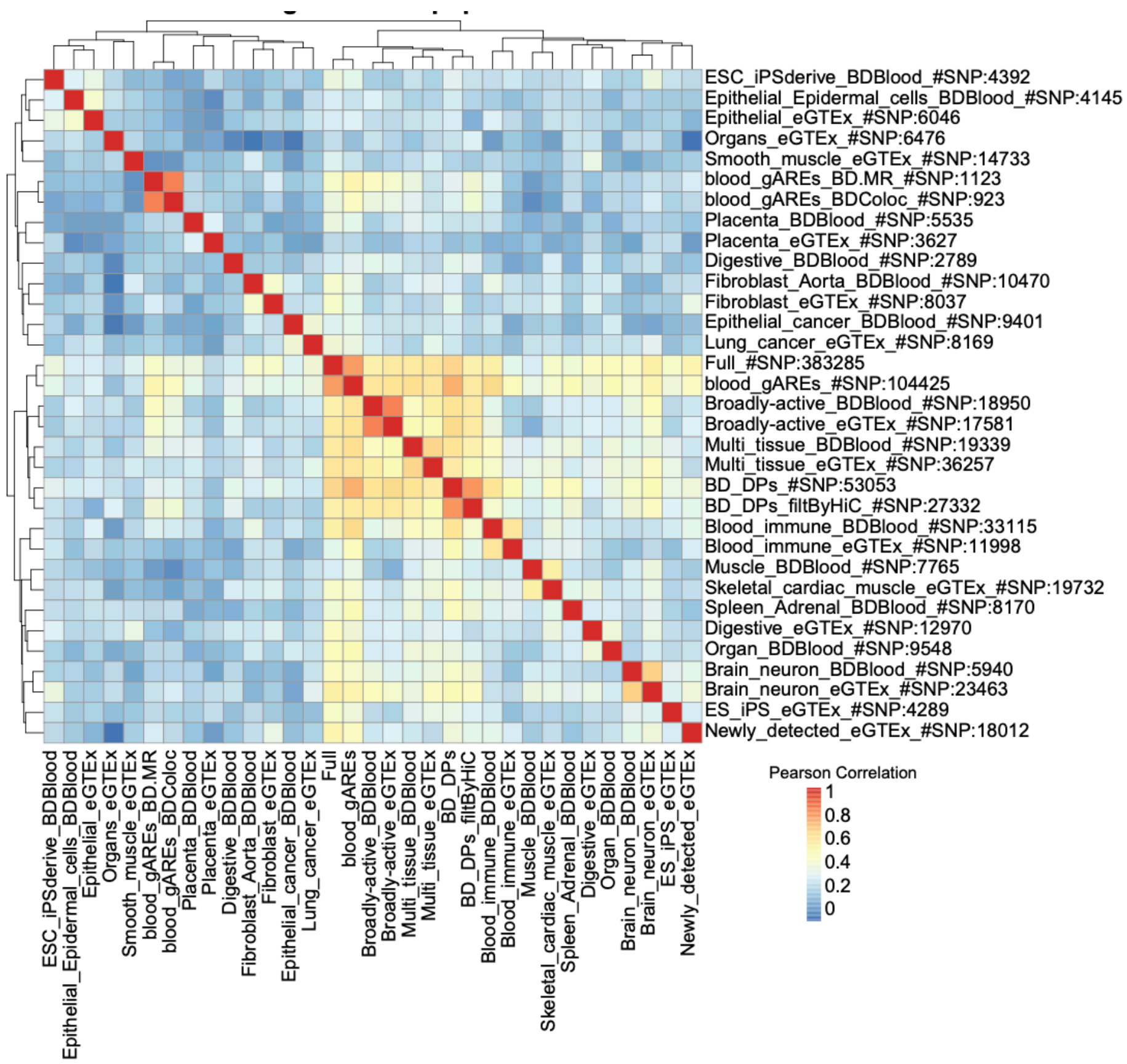

Response Fig. 4. Correlation among different Epi-partitioned PRSs

-page9, line 28: I think it is noteworthy that all PRS significantly different between cases and controls are either based on overlap with blood, multi-tissue or placenta data. This is quite interesting given that placental biology has been linked to psychiatric disorders.
Thank you for highlighting this important point. We have now added "placenta" to the main results to emphasize its relevance. This observation suggests that the blood–placenta–brain axis may represent a novel and promising direction for future research into BD biology.

Discussion
-page 11, line 36: as you note BD has been associated with elevated immune markers, did you also find this in your dataset based on the EHR data?
Thank you for the question. In addition to testing EHR term enrichment for each BD patient subgroup against the rest of the BD cohort, we also compared each group to all control samples. As expected, many enriched terms were psychiatric in nature, such as "Bipolar disorder," "Eating disorders," "Alcohol abuse," "Suicidal ideation," and "Cannabis abuse."

We also identified several non-psychiatric terms that were enriched in BD subgroups compared to controls, including “Leukocytosis, unspecified” (P = 0.01) and “Pain in joint involving hand” (P = 0.02) in Group 1, “Constipation, unspecified” (P = 0.04) and “Impaired glucose tolerance test (oral)” (P = 0.02) in Group 3, and “Hypertension” in Group 4 (P = 0.01). These findings are consistent with the enrichment results obtained when comparing patient subgroups against other BD patients.

Together, these results suggest that the blood epigenomic profiles not only reflect overall case–control differences, such as elevated immune activation, but also capture meaningful heterogeneity within the BD population. In particular, some subgroups appear to show stronger inflammatory or metabolic signatures, supported by both EHR terms and epigenomic patterns.

We have amended to main text as follows: “Importantly, we observed similar EHR enrichment patterns when comparing each subgroup to controls, including leukocytosis (P=0.01) and joint pain (P=0.02) for Group 1, constipation (P=0.04) and impaired glucose tolerance test (P=0.02) for Group 3, and hypertension (0.01) for Group 4, further confirming that the latent factor-based clusters capture meaningful clinical heterogeneity.” We have also amended the Discussion as follows: “(6) EHR phenotypic validation**:** Finally, we leveraged clinical data to test whether patient subgroups defined by epigenomic profiles were associated with immune-related phenotypes. Indeed, inflammation-related EHR terms such as *leukocytosis* and *joint pain* were enriched in specific BD subgroup (group 1), providing clinical support for the immune dysregulation hypothesis”.

-page 13, line 44: limitations: even though the largest sample, it is still a limited number of BD patients, mainly white non-Hispanic. This could influence generalizability, especially of the PRS and hQTLs. Furthermore, no replication dataset is available.
Important comments! We have added the following text to the last part of the discussion: “Finally, our study cohort consists primarily of non-Hispanic White individuals diagnosed with Type I bipolar disorder. While this enhances the accuracy of PRS-based analyses and reduces heterogeneity within the cohort, it also limits the generalizability of our findings to other ancestral populations and to individuals with type II BD”.

Are any comparable datasets for other psychiatric disorders available which could be used to at least compare the number of identified dCREs?
We thank the reviewer for this insightful question. To our knowledge, there are currently no publicly available datasets with sufficient sample size that profile histone modifications or chromatin accessibility (e.g., ATAC-seq) in peripheral blood immune cells from individuals with psychiatric disorders. Most available epigenomic profiles in psychiatry, such as those from the PsychENCODE consortium, have focused on postmortem brain tissue or stem cell-derived neuronal models. In contrast, peripheral blood datasets have largely been limited to DNA methylation, leaving two major gaps in the field: (1) the lack of cross-modality mapping between DNA methylation and cis-regulatory element (CRE) profiles defined by histone ChIP-seq or ATAC-seq, and (2) the limited integration between brain- and blood-based regulatory landscapes.

Our study represents the first large-scale ChIP-seq effort across five histone marks in peripheral immune cells from BD patients and matched controls, enabling the identification of

tens of thousands of differential CREs and their genetic and clinical associations. We agree that future comparative studies across psychiatric disorders—and across molecular modalities in peripheral immune cells—will be highly valuable. Our dataset lays the foundation for such integrative and comparative analyses.

We have also added the following sentence to the last paragraph of our Discussion to reflect this point "Our datasets also offer a unique epigenomic modality that complements existing blood transcriptomic and methylation-based studies and opens new avenues for cross-tissue and cross-modality comparisons."

Methods

Sample description.

Rich data on cases and controls are available. A table summarizing potential confounds, including medication, psychiatric comorbidity, somatic disease, smoking would be very helpful. Also could the PRS for BD be shown for cases vs. control.

We have included sex, age, case/control in the Supplementary table 1. We have also included other EHR information in our GEO submission. We have also included various PRS scores for each individual in Supplementary Table 6.

It appears that cases and controls were collected from different biobanks– information on comparability of collection procedures and storage are lacking so that batch effects cannot be excluded.

We appreciate the reviewer's concern regarding potential batch effects due to case and control samples originating from different biobanks. Below we provide detailed information on sample processing and quality control measures to assess and mitigate batch-related confounding:

Sample Processing and Quality Control

Buffy coat samples were processed in 62 mini-batches, each containing mostly 3 samples (with a few batches of 2 or 4). All five histone marks were processed simultaneously per sample, using our previously published titration-based and normalized ChIP-seq protocol (Caride et al., 2023; https://link.springer.com/article/10.1186/s12864-023-09253-0). This method ensures robust experimental consistency across batches. In that study, we demonstrated consistent ChIP yields and high reproducibility in peak numbers across 412 samples processed over 1.5 years (see Figure 4B and Additional file 12).

We performed rigorous quality control at multiple stages:

Pre-library QC:

- Fragmentation to mononucleosomes verified by fragment analysis
- Chromatin prep optimized based on pellet size
- ChIP reactions titrated per chromatin input
- Same-lot antibodies used throughout

Library QC:

- Enrichment confirmed by qPCR using histone mark-relevant loci vs. a chr19 gene desert control

Batch-Case Confounding and Evaluation

Due to logistical constraints from working with different hospital-based cohorts, cases and controls could not be mixed within batches: only 2 of 62 batches included both. While this could raise concern about serious confounding, it's important to note that batch effects are generally random and unlikely to consistently shift the epigenomic profiles of all case batches away from those of control batches.

To evaluate the impact of batch effects on differential analysis, we conducted several diagnostic tests:

1. **Batch label permutation (Response Fig. 5):**
   To assess whether batch effects could be driving the observed differences between BD and control samples, we performed a label permutation test. Specifically, we randomly permuted the batch labels (case vs. control) 500 times, preserving the original number of batches assigned to each group, followed by the same differential analysis strategy. If substantial batch effects were systematically shifting the epigenomic profiles beyond the true biological signal, we would expect similar or even greater numbers of differential CREs after permutation. However, as shown below for each histone mark, the number of detected differential CREs (X-axis) dropped substantially compared to the observed value (red dashed line), with fewer than 1% of permutations yielding more differential CREs than observed for four of the histone marks (2.22% for H3K4me3). This indicates that the differential signals are more likely driven by biological differences between BD and control groups rather than by batch assignments.

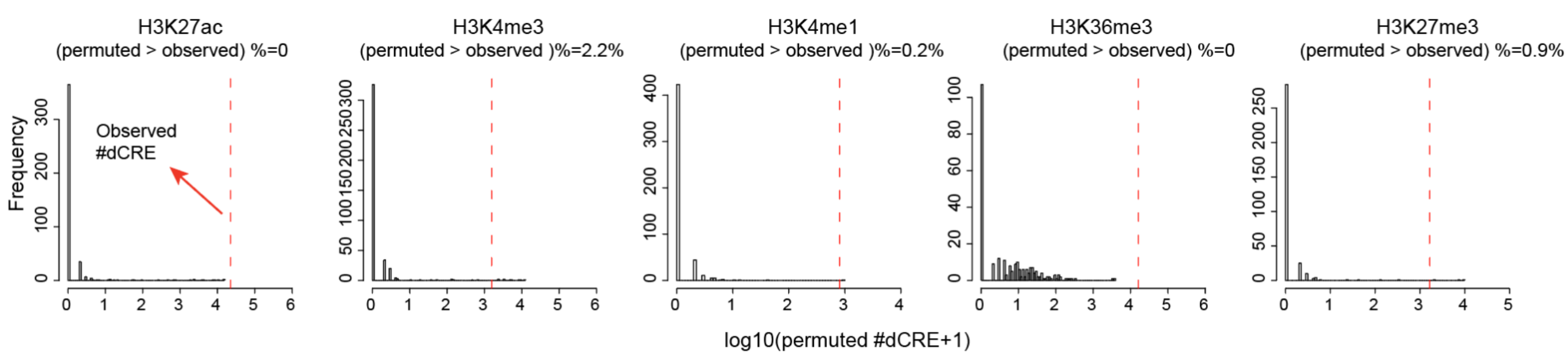

Response Fig. 5. dCREs detection with batch label permutated.

2. **Robustness of differential signal across each sample (Response Fig. 6):**
   To further distinguish true biological signals (BD vs. control) from technical noise (batch effects), we assessed the robustness of differential signals across samples. For each individual sample, we conducted differential analyses against all samples from either the BD or control batches. As shown below, comparisons across biological groups (e.g., a BD sample vs. control batches, in red) consistently produced more similar differential signals, reflected by higher cross-CRE correlations of differential *t* statistics among samples. In contrast, comparisons within the same group (e.g., a BD sample vs. BD batches, in blue) resulted in correlations centered around zero, suggesting these reflect random noise rather

than systematic effects. These results support that the observed differential signals are primarily driven by biological differences rather than batch-related artifacts.

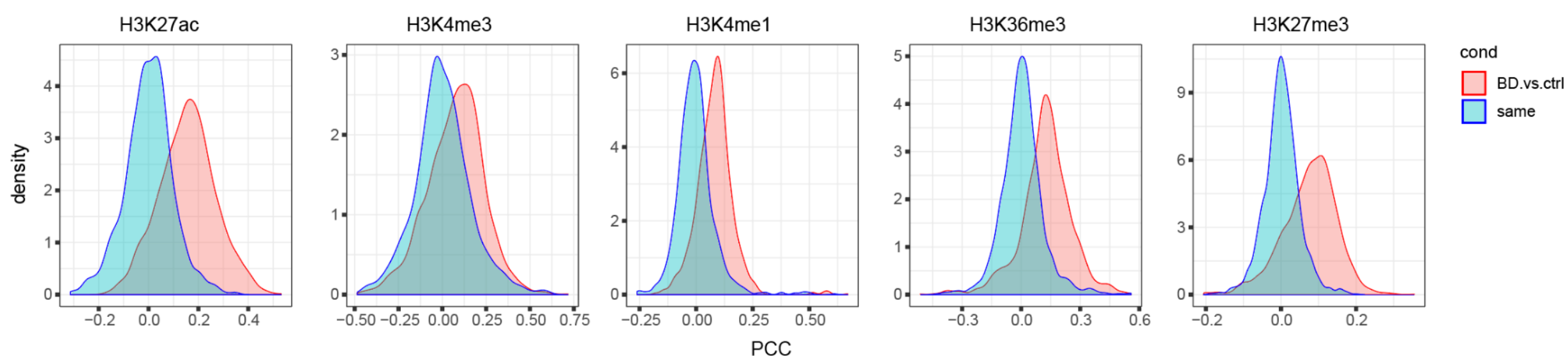

Response Fig. 6. Robustness of differential signal between one sample vs. samples from a different group.

3. **Residual Pattern Analysis (Response Fig. 7):**
   We finally tested whether residuals for each CRE from the previous BD vs. control differential analysis showed a strong clustering pattern for each batch. Using principal component analysis, our results below (H3K27ac as an example, similar for other marks) showed no clear clustering by batch (samples in each batch highlighted in red in each panel), indicating that batch structure does not drive major variation after accounting for disease status.

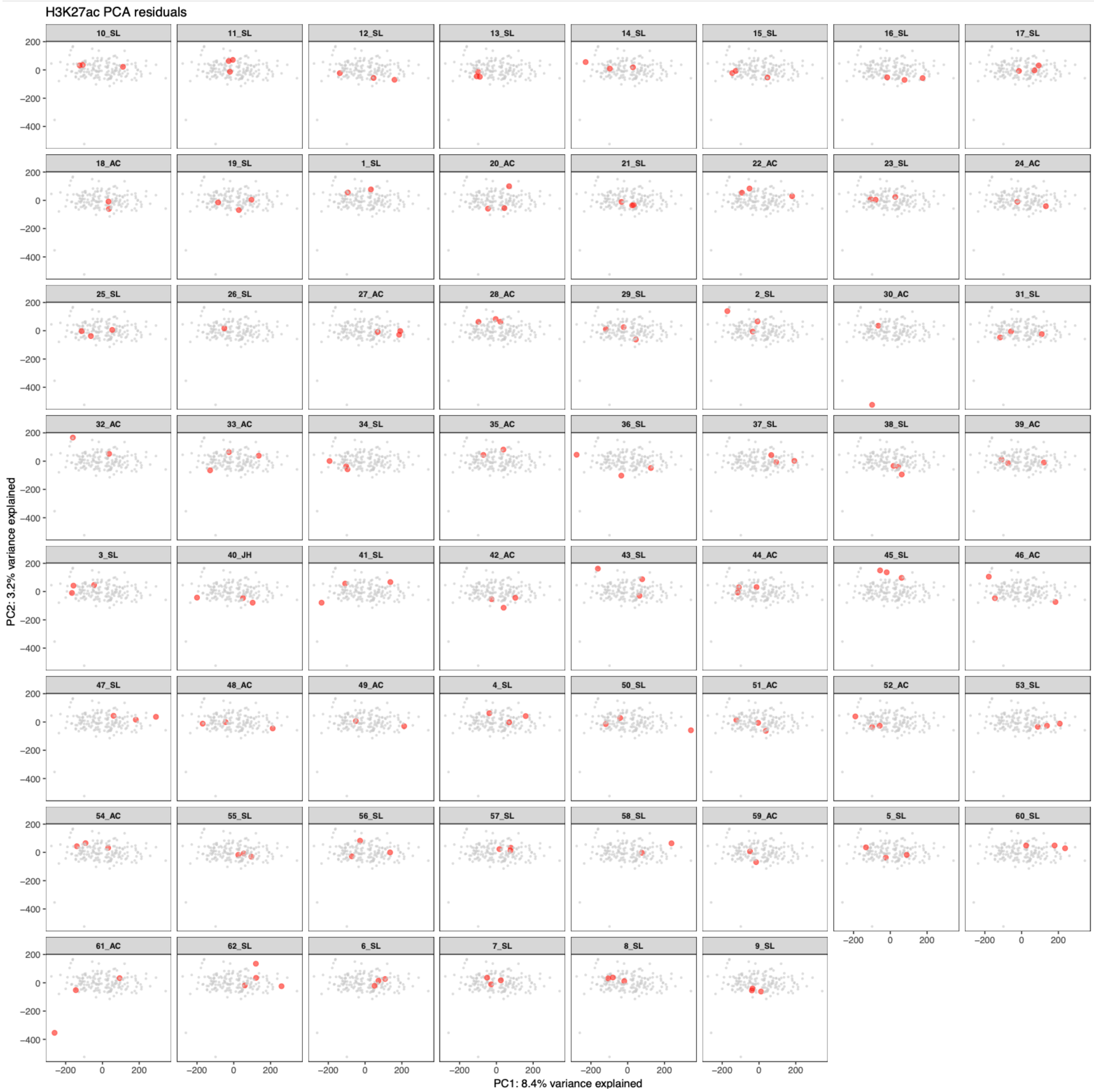

Response Fig. 7. Residuals of samples from the same batch spreading out ins PCA plots

Conclusion

While we acknowledge that batch confounding cannot be entirely ruled out, multiple lines of evidence suggest that batch effects are unlikely to drive the observed BD vs. control differences. The consistency of our differential signals across multiple mini-batches and control analyses supports their biological relevance. We have added this clarification to the Methods section to better address this concern.

We have added a paragraph under Differential CRE analysis and motif enrichment in methods and Supplementary Figures for clarification. “Due to logistical constraints, cases and controls were processed in separate batches, with only 2 of 62 containing both. To evaluate whether batch structure confounded differential analyses, we performed several checks. First, permutation of batch labels (case vs. control) showed a significant reduction in the number of differential CREs across 500 iterations, indicating the observed signals were unlikely to arise from batch effects (**Supplementary Fig. 2c**). Second, differential comparisons between individual samples and opposing group batches produced more consistent CRE-level signals than within-group comparisons (**Supplementary Fig. 2d**). Third, principal component analysis of model residuals showed no clustering by batch (results not shown). Together, these analyses suggest that batch effects are unlikely to drive the observed BD vs. control differences.”

ChIP Seq:
More detail is needed on how samples were randomized and processed for ChIP-Seq and how possible batch-effects were dealt with.
Please refer to the answers above.

Also were Tier 1 and Tier 2 samples equally distributed between cases and controls?
We examined the distribution of Tier 1 (T1) and Tier 2 (T2) samples between cases and controls. As shown in the table below, the distribution varies across histone marks. In general, fewer T1 samples are from cases compared to controls, while T2 samples are more enriched in cases.

Percentage in cases	H3K27ac	H3K27me3	H3K36me3	H3K4me1	H3K4me3
T1	45%	47%	43%	21%	19%
T2	73%	58%	51%	63%	66%

To understand this imbalance, we further compared the underlying QC metrics, including total reads and relative strand correlation (RSC), between cases and controls. As shown in the figures below (**Response Fig. 8)**, for each histone mark, cases consistently had significantly fewer total reads, which primarily explains the lower proportion of T1 samples among cases.

RSC scores, which indicate signal-to-noise ratio, showed no significant difference between cases and controls for H3K27ac, H3K27me3, and H3K36me3. For H3K4me1 and H3K4me3, RSC scores were higher in controls, likely due to their higher read depth.

Importantly, read depth was normalized, and technical covariates were properly adjusted for in all downstream differential analyses. Therefore, we do not expect these differences in sample quality distribution to substantially affect our results.

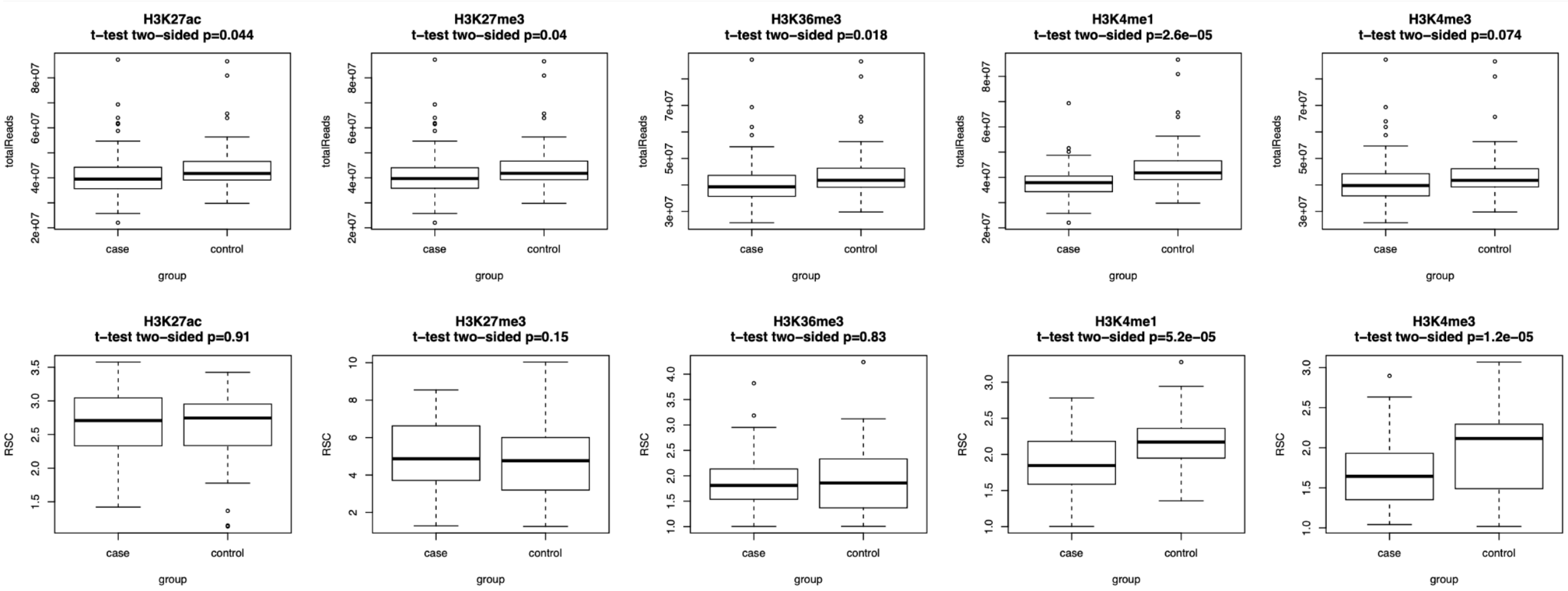

Response Fig. 8. Comparisons of QC metrics between case and control across histone marks

WGS

-page 19, line 13: reads were aligned based on hg38, however later it is stated that ChIPSeq peaks were aligned based on hg19. Was any up/downlifting performed to map these annotations?

Important point! We used UCSC liftover to transfer from different genome assembly builds. We have added the following text to the hQTL mapping section in methods: "To ensure alignment with the genotype matrix in GRCh38, we used UCSC LiftOver [HL1] to map CREs defined by various histone marks from hg19 to GRCh38".

Driver genes priorization

-page 23, line 13: what does "worked significantly better" exactly mean?

Thank you for the comment. We quantified performance using the Area Under the Precision-Recall Curve (AUPRC). As shown in Supplementary Fig. 4c, the AUPRC based on the original gLink scores was consistently higher than those from the 50 permutations of gLink scores within each CRE–gene distance bin, demonstrating improved predictive power over random expectation. To clarify this point, we have revised the text as follows: "We permuted gLink scores within each CRE–gene distance bin 50 times and evaluated performance using the area under the precision-recall curve (AUPRC). The AUPRC based on the original gLink scores was consistently higher than that from the permuted scores, indicating improved predictive power over random expectation (Supplementary Fig. 4c)".

Epi-partitioned PRS

-page 24, line 1: more information on the construction of PRS is needed. Which program was used? From the reference, it seems that you used PRSice which is based on clumping and thresholding. If so, which p-value threshold was chosen? Usually, using all SNPs and correction for LD between them performs better than clumping and thresholding, e.g. using PRScs or LDpred.

We thank the reviewer for this important comment. Our original analyses employed a clumping and thresholding (C+T) approach just like PRSice. We used GWAS *P* threshold 0.05 and 0.1,

reaching similar results, and we only show results based on threshold of 0.05. We have added "GWAS *P*<=0.05 or 0.1, results only shown for *P*<=0.05" to the Epi-partitioned PRSs section of the methods.

As the Reviewer mentioned, Bayesian methods like PRS-CS are generally considered superior for overall polygenic prediction by modeling LD structure genome-wide. To evaluate the robustness of our epi-partitioned PRS framework across different PRS construction methods, we applied both a clumping-and-thresholding (C+T) approach and a Bayesian shrinkage model (via PRS-CS), using identical sets of CREs.

As we visualized the correlation between C+T-based and PRS-CS-based scores across matched epigenomic partitions (**Response Fig. 9**), we found that the strongest correlations were consistently between the same partition (e.g., full model, immune, ubiquitous, or gCRE sets) regardless of the method used.

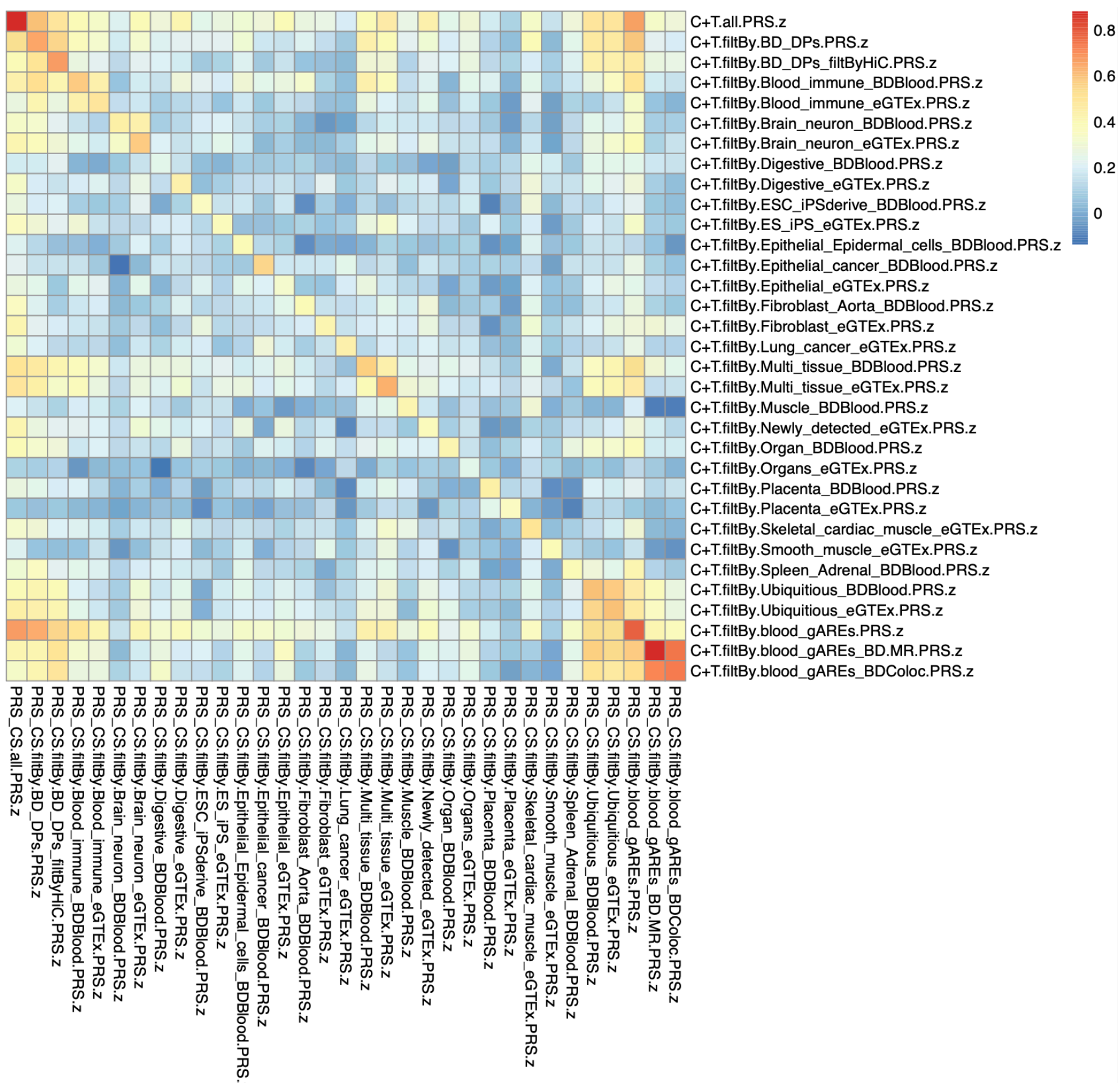

Response Fig. 9. Correlations betwen PRSs with C+T and PRS-CS.

We also found that several key results remained robust, including: (1) PRSs partitioned by CRE groups primarily active in the immune system and blood gCREs identified in our blood study continued to differentiate BD patients from controls and (2) PRSs based on gCREs associated with BD via GWAS-QTL colocalization or Mendelian randomization still distinguished patient subgroup 3 and remained correlated with the epigenomic latent factor LF9, reinforcing their value in capturing inter-individual heterogeneity. However, we note that associations with

CMV reactivation markers (e.g., CMV IgM) observed in the original C+T-based analysis did not replicate under the PRS-CS model, possibly due to differences in effect size calibration or sparsity patterns. Despite of these, these results still support the central conclusion in the main text: “the integration of profiles based on five epigenomic marks could capture the heterogeneity of the patients—which may arise from a complex interplay of genetic factors, medicine taken, and physiological conditions—providing a unique perspective for therapeutic intervention.”

Importantly, while Bayesian methods are known to improve prediction accuracy in general settings, **there is currently limited evidence on their superiority in uncovering biological or clinical heterogeneity when PRS are partitioned by regulatory annotations**. Given this, and the fact that our key biological conclusions were robust to both frameworks, we believe our use of C+T is justified for the purpose of stratifying patients. We have added this to the Methods section for clarity:

“To evaluate the robustness of our epi-partitioned PRS framework across different PRS construction methods, we applied both a clumping-and-thresholding (C+T) approach and a Bayesian shrinkage model (via PRS-CS), using identical sets of CREs. Notably, when visualizing the correlation between C+T-based and PRS-CS-based scores across matched epigenomic partitions (**Extend Data Fig. 5g**), we found that the strongest correlations were consistently between the same partition (e.g., immune, ubiquitous, or gCRE sets) regardless of the method used. We also found that several key results remained robust, including: (1) PRSs partitioned by CRE groups primarily active in the immune system and blood gCREs identified in our blood study continued to differentiate BD patients from controls; (2) PRSs based on gCREs associated with BD via GWAS-QTL colocalization or Mendelian randomization still distinguished patient subgroup 3 and remained correlated with the epigenomic latent factor LF9. This observation supports the robustness of our epi-partitioning strategy in capturing biologically meaningful inter-individual variation.”

Reviewer #1 (Remarks on code availability):

NA

Reviewer #2 (Remarks to the Author):

I co-reviewed this manuscript with one of the reviewers who provided the listed reports. This is part of the Nature Communications initiative to facilitate training in peer review and to provide appropriate recognition for Early Career Researchers who co-review manuscripts.

Reviewer #3 (Remarks to the Author):

Hou et al. present a very interesting manuscript exploring the immune-psychiatry axis in bipolar disorder through the generation and integrative analysis of genomic, epigenomic and health record data. The dataset will be a valuable resource for the research community and the results offer intriguing insights for further analysis.

We appreciate the very positive comments from the Reviewer.

Please find comments listed below:
• For readability, needs a clear definition in introduction of what a “CRE” vs a “candidate CRE” is and how they differ from a histone peak. Furthermore, how do you know the regulatory elements act in cis?
We appreciate the reviewer’s comment. In our study, we follow the convention established by the ENCODE project, where candidate cis-regulatory elements (cCREs) refer to genomic regions marked by characteristic chromatin features (e.g., open chromatin, H3K27ac, or H3K4me1 peaks) that are predicted to have regulatory potential. In this manuscript, we use the term CRE to refer broadly to such regions identified from histone ChIP-seq peaks, without further distinguishing between candidate and experimentally validated elements, to make it easier to distinguish between dCREs (differential CREs) and gCREs (genetically driven CREs), and also to maintain consistency with our previous work and other large-scale epigenomic studies.

Regarding “cis,” the term historically denotes regulatory elements that act on nearby genes along the same chromosome, in contrast to trans-regulatory factors such as transcription factors or RNA-binding proteins. While some CREs can exert long-range or inter-chromosomal effects, their predominant mode of action, inferred from epigenomic assays, is cis-regulation, which is the context used here.

Now we have added sentences to clarify the definition of CRE in the manuscript: “We annotate 450k candidate cis-regulatory elements (CREs) based on ChIP-seq peaks” in the abstract, and “We first identified on average 8.4k *cis*-regulatory elements (CREs), defined as peaks in ChIP-seq profiles, across the five histone marks” in the introduction.

• Heterogeneity in histone peaks within the cohort has been investigated. However, it is unclear how many of the 800k+ CREs are common across subjects within control and bipolar groups. These data would simply illustrate inter-individual variability for histone peaks.
Thank you for raising this important point. In fact, exploring case-control differences in histone peaks was a key motivation of this project. After generating a merged peak set across individual

samples, we examined how frequently each merged peak is detected across the cohort (i.e., the proportion of samples in which a peak is present) (**Response Fig. 10**). As illustrated in the left panel of the figure below (H3K27ac as an example, all other histone marks showing a similar pattern), a large fraction of peaks are present in fewer than 50% of samples, highlighting substantial inter-individual variability. This variability likely reflects both technical factors (e.g., sequencing depth, local signal-to-noise ratio) and biological variation (e.g., dynamic chromatin states across individuals). We further compared peak presence frequencies between cases and controls, and as shown in the right panel, most peaks exhibit similar detection rates between the two groups.

These findings suggest that defining "case-specific" or "control-specific" peaks based solely on presence/absence is not statistically robust, due to high variability within groups and limited power to detect subtle differences. Instead, we chose to rely on quantitative differential analysis that properly adjusts for key covariates (e.g., cell type composition, age) to identify robust and biologically meaningful case–control differences in histone signal.

We have added one sentence at the start of the differential CRE section in the main text: "Due to high inter-individual variability in peak detection and the lack of statistical robustness in defining BD-specific CREs, we then focused on whether the activity of CREs in blood is different in BD patients compared to controls."

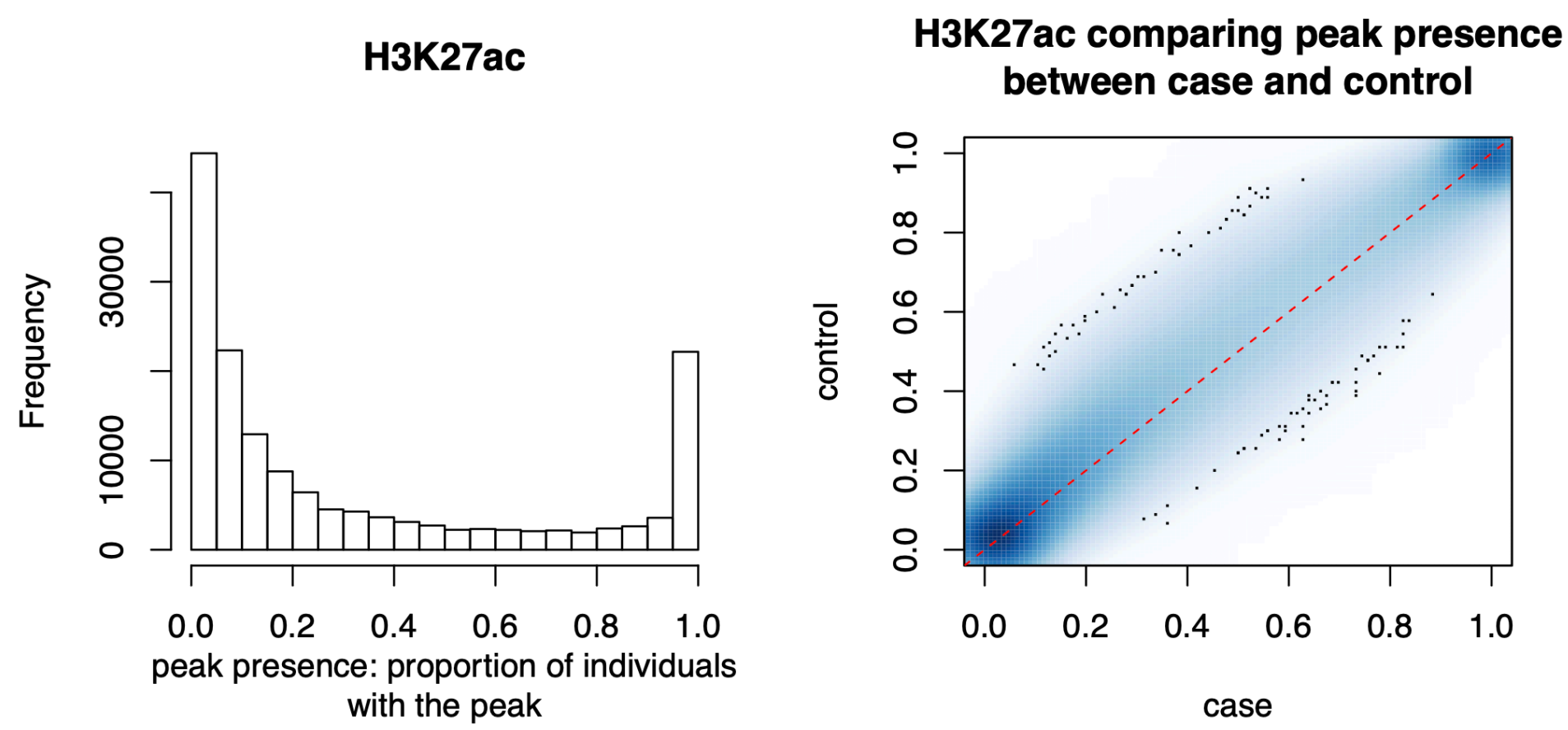

Response Fig. 10. H3K27ac CRE presence across individuals.

• Relating to Figure 1b, the results section would benefit from a brief description of the method for clustering profiles into 84 modules and 13 groups. Also, in the figure legend for this figure, it seems the axis labels are in opposite orientation. 242 reference epigenomes are columns (bottom) and 84 modules are rows (left).

Thanks for the suggestion and great catch! We have now added a brief introduction of the clustering as follows: "We then binarized CRE activities based on these profiles across tissue and clustered them into 84 modules and 13 groups (G1-G13, **Fig. 1b**) using the k-centroids algorithm with the Jaccard distance as in EpiMap". We have also modified the figure legend accordingly.

• [Page 4, Line 36] “These results confirm an elevated level of inflammation and reduced adaptive immune response in BD” – these results support this notion but are not confirmatory, I suggest rewording this statement to reflect this.
We agree, and we now replace “confirm” with “suggest”.

• [Page 4, Line 45] “15 were associated with BD, 46 based on GWAS signals, differential signals, or mechanistic evidence” – mechanistic evidence requires supporting citations to be added to Supp. Table S3 or in text.
Thanks for reminding us! We have now added the citation to both the main text and the table.

• [Page 5, Line 29] Define “peer factors” in explanation of hQTL analysis.
Thanks for catching that! We now clarified it by “identify latent factors inferred by a probabilistic model of probabilistic estimation of expression residuals (referred to as PEER factors)”, and replace all the “peer factors” with “PEER factors”.

• [Page 5, Line 33] It is unclear how the millions of hQTLs map to thousands of CREs, I suggest further explanation around this process. Are these hQTLs loci or specific SNPs with significant associations?
Thanks for the suggestions! Here, hQTLs refer to specific SNPs significantly associated with histone modification levels at CREs, where multiple SNPs may map to a single CRE due to linkage disequilibrium or polygenicity. To clarify this, we have revised the first sentence of the paragraph as follows: “To dissect BD-associated genetic variants, we next sought to identify the impacts of genetic variants on CRE activity by mapping histone quantitative trait loci (hQTLs), i.e., specific SNPs significantly associated with histone modification levels at CREs across individuals. These CREs are referred to as genetically influenced CREs (gCREs).”

• [Page 5, Line 43] “We also identified 4k gCREs shared between H3K27ac and H3K4me1, and 1k gCREs shared between H3K27me3 and H3K4me1 (Fig. 3a).” – how is sharing defined? Much of authors’ interpretation depends on sharing or not sharing; needs to be clear early on in text how this is defined. Would you need to do coloc to confirm these are shared?
Great catch! It means simply genomic “overlap” in this sentence, while “sharing” in the later text of “QTL sharing” means shared genetic regulation. We agree it is very confusing. We now replaced revised the sentence to “We also identified 4k genomic regions with overlapped gCREs from H3K27ac and H3K4me1, and 1k regions with overlapped gCREs from H3K27me3 and H3K4me1 (**Fig. 3a**). Following our previous studies on QTL sharing across tissues based on directionality consistency of lead QTL effect size[5], we revealed pairs of histone marks with higher degree of hQTL sharing”.

We agree coloc is more rigorous way to identify shared genetic regulation between individual pairs of CREs. However, here, we were only interested in the global level of sharing between histone marks; therefore, we decided to use a more simply strategy based on directionality consistency of head QTL effect size we established in our previous works[5,6].

• Results section generally light on the how analysis was approached, e.g. deconvolution described as ‘our method’, brief description within results text would be useful.

Thanks for the suggestion. We have added a brief description of deconvolution as follows: " We therefore estimated for each patient the cell fractions of six major white blood cell types, including CD4⁺ T cells, CD8⁺ T cells, B cells, natural killer cells, monocytes, and neutrophils, with our in-house deconvolution approach based on cell-type-specific CRE activity from reference epigenomes".

• Clustering needs some analysis of stability to participant inclusion to be convincing (e.g. subsampling).

Thank you for the suggestion. We interpreted "clustering" as referring to the participant clustering based on MOFA latent factors shown in Fig. 5a. To assess robustness, we performed a subsampling analysis by randomly retaining 90% of the samples, repeating the hierarchical clustering, and comparing the resulting clusters to the original clustering using the Adjusted Rand Index (ARI) (**Response Fig. 11**). ARI quantifies clustering similarity based on pairwise sample agreements, adjusting for chance. We repeated this procedure across a range of cluster numbers (k = 3 to 7). The result below shows a modest robustness of clustering (ARI=0.2-0.5), which is acceptable considering the small sample size (N=180), and a mild increase in ARI values with k = 5, supporting the choice of five clusters as a reasonable and stable solution.

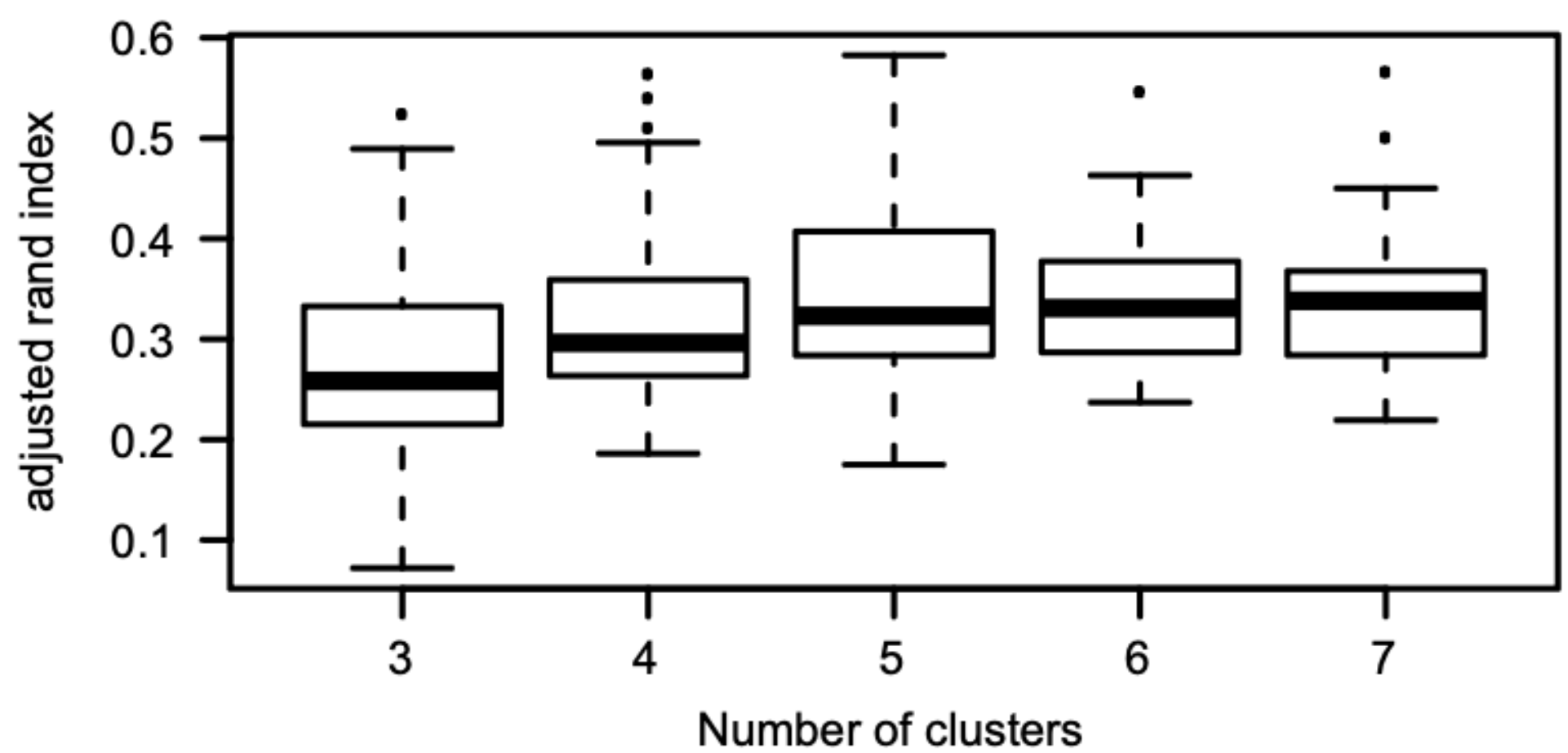

Response Fig. 11. Robustness of clustering across different cluster numbers.

We have also added this result to **Supplementary Fig. 5d**, and one sentence to the Methods section on patient subgrouping as follows: "To assess clustering robustness, we conducted a subsampling-based (90% samples) stability analysis using the Adjusted Rand Index (ARI) from R package mclust (v.5.4.3) (**Supplementary Fig. 5d**), which supported the selection of five clusters for downstream analysis".

• Why look for LDSC enrichment in this smaller dataset of groups within dataset rather than in whole of EpiMap? How do the G groups relate to EpiMap groups?

Thank you for the insightful question. While EpiMap provides a comprehensive reference of CREs across a wide range of tissues (~2.1 million elements), our primary goal was to understand the tissue-sharing patterns specifically for the 169k CREs active in blood identified

from our data. Applying the same k-centroids clustering approach as EpiMap allowed us to define 13 tissue-sharing groups (G1–G13) based on the activity of blood CREs across 242 reference epigenomes. Because the underlying CRE set in our study is distinct and much smaller than that of EpiMap, the resulting groupings are not directly comparable to EpiMap's global clusters. Therefore, we performed LDSC enrichment within these blood-derived CRE groups to assess the functional relevance of regulatory programs captured by our dataset, rather than reusing predefined EpiMap groups which may not reflect blood-specific regulatory architecture.

• In relation to the clustering of participants into 5 groups, dCREs which are differential elements between cases and controls were used for MOFA to identify latent factors, yet cases and controls were poorly discriminated in the group clustering, it would be useful to add discussion on why this may be the case.
We appreciate this important point. While MOFA latent factors were derived from dCREs (with permissive cutoff) and not explicitly optimized for case-control separation, we did observe that clusters 1 and 4 were enriched for BD cases, whereas cluster 5 was enriched for controls (shown in Fig. 5b). This suggests that certain epigenomic regulatory patterns partially reflect diagnostic status. Still, individual-level heterogeneity and shared profiles across cases and controls remain substantial. Since our goal was to capture axes of biological variation across all individuals, especially among patients, rather than to build a discriminative classifier, this heterogeneity partially orthogonal to diagnostic labels is still biologically meaningful, supported by EHR enrichment.

We have added the following text to the Discussion: "Notably, clusters 1 and 4 were enriched for BD cases, while cluster 5 was enriched for controls (**Fig. 5b**), suggesting that the subtyping partially recapitulates diagnostic status. However, the clusters also captured phenotypic and biological heterogeneity beyond diagnosis, as evidenced by their enrichment for diverse phenotypic, clinical, and treatment features, highlighting the clinical relevance of our findings".

• Following this clustering, enrichment of EHR terms was then performed exclusively on cases within these groups, yet the controls contributed to the grouping. How does this enrichment result change with/without the controls included?
Thank you for the helpful suggestion. Our primary goal was to characterize heterogeneity among patients, that's why we focused on EHR enrichment analysis within patient subgroups in the manuscript. Besides, some clinical data are available for most of the patients but not for all control donors, such as CRP protein level.

Nonetheless, we performed EHR enrichment across the full cohort (patients + controls), grouped by the same clustering (**Response Fig. 12)**. The overall patterns were consistent with the patient-only results shown in Fig. 5c: Groups 1 and 2 remained enriched for infection- and inflammation-related terms, group 3 for impaired glucose tolerance, and group 4 for quetiapine

usage and hypertension. Some additional terms emerged, such as substance use–related annotations (e.g., nicotine and cannabis) in group 1. These results further support the link between blood epigenomic profiles and physiological states.

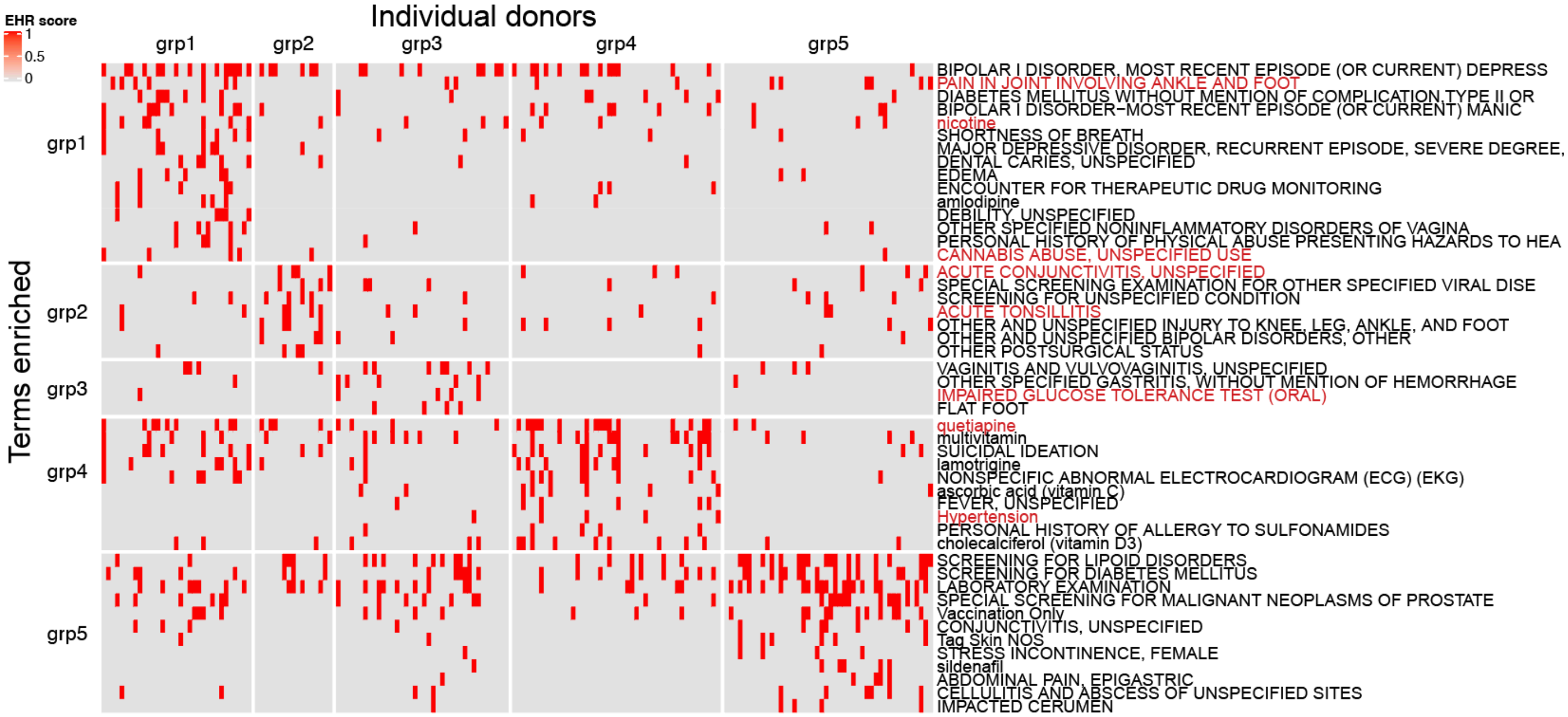

Response Fig. 12. Enrichment of EHR terms for subgroups of all donors.

However, including controls in the enrichment analysis introduces interpretational challenges, as the proportion of controls varies across groups. This imbalance can bias enrichment results and confound group-specific signals. For example, a lot of new terms appear for group 5 now due to a large fraction of control donors (80%) in this group. For this reason, we chose to focus on patient-only enrichment in the manuscript.

• The authors found that genes near BD GWAS-colocalizing gCREs related to ER function, potentially implicating ER function in BD pathogenesis. How do the pathways highlighted by coloc with hQTLs differ from pathways highlighted by coloc with eQTLs? In a direct comparison, how correlated is pathway enrichment by blood hQTL with enrichment by blood eQTL? It's of significant interest to understand how the information from different omic layers differs.

Thank you for this thoughtful comment. We agree that it is important to understand the shared and different genetic signals and downstream biological pathways between epigenomic and transcriptomic layers.

To this end, we examined GO term (biological process) enrichment for genes supported by BD GWAS–eQTL colocalization in **brain (36 genes)** and **blood (63 genes)** (Fig. 4b), using EnrichR. We compared these results to those terms highlighted by **hQTL-based colocalized gCREs (Fig. 3c)**. While the brain and blood eQTL-derived gene sets showed some overlap–for example, both were enriched for *regulation of protein localization to chromosome, telomeric region*–they also differed: brain eQTLs pointed to terms, such as *IRE1-mediated unfolded protein response* (P=0.011), *glutathione catabolic process* (P=0.008), and *membrane lipid biosynthesis* (P=0.003), whereas blood eQTLs highlighted terms, such as *to Golgi vesicle-*

mediated transport (P=0.005), and "Regulation of Fc Receptor Mediated Stimulatory Signaling Pathway" (P=0.015), more consistent with the results based GWAS-hQTL colocalization (**Fig. 3c**). These differences likely reflect the tissue-specific regulatory landscape and suggest that BD genetic risk may impact distinct yet biologically related processes in brain and blood.

Importantly, several processes were uniquely enriched among hQTL-colocalized gCREs but not in either eQTL gene set, such as *negative regulation of neurotrophin TRK receptor signaling pathway*, *regulation of replicative cell aging*, *caveolin-mediated endocytosis*, and *retrograde vesicle-mediated transport, Golgi to ER,* (Fig. 3c). These hQTL-specific enrichments underscore how the epigenomic layer can capture upstream regulatory perturbations that may not manifest at the transcriptomic level.

Together, these results highlight the shared yet **complementary roles** of eQTL and hQTL analyses in capturing distinct aspects of BD genetic risk, with the epigenomic layer offering additional resolution on chromatin-level regulatory disruptions, particularly in circulating immune cells.

We have included the following sentences to the text discussing Fig. 4b: "We then used EnrichR to identify enriched GO terms for both gene sets. Notably, only blood-eQTL-based candidate genes enriched terms such as *Golgi vesicle-mediated transport* (P=0.005) and *regulation of Fc receptor mediated stimulatory signaling pathway* (P=0.015), closely mirroring our findings based on blood hQTL-colocalized gCREs (Fig. 3c), suggesting convergence between BD transcriptomic and epigenomic signals."

• [Page 9, Line 43] It is unclear how the medication GO enrichment was performed to derive the 6 immune related medications described, further clarification required.

Thanks for the suggestion. We have edited the sentence for clarification: "GO enrichment analysis showed that the CREs affected by six of these drugs enrich genes from immune-related pathways". We have also added details to the main Methods section as follows: "Enrichment of nearby genes for dCRE associated with BD or drug response is calculated by R package rGREAT (v1.2.1)[4] with all CREs detected for each histone mark as background (**Fig. 2A, Fig. 6A**).".

• In relation to the medication repurposing analysis, these medications largely act on proteins, yet the analysis is mostly based on epigenomic profiles. This inference limitation should be discussed. In addition, this analysis could be expanded to include the mode of action of these medications, are any of them modifiers of the epigenome or transcriptional machinery?

Thank you for this important comment. We agree that most drugs act on proteins, whereas our drug repurposing analysis is based on epigenomic signatures, which represent downstream regulatory consequences rather than direct drug targets. Our analysis therefore rests on the assumption that changes in histone modification patterns can serve as a surrogate for transcriptional states, analogous to gene expression–based drug repurposing frameworks such as Connectivity Map. Under this framework, drugs are prioritized based on their ability to reverse disease-associated regulatory programs, rather than by directly targeting the epigenomic machinery itself.

Importantly, while this approach does not imply direct physical interaction between drugs and chromatin regulators, we note that a subset of the identified compounds do target

transcriptional or epigenomic regulators, providing biological plausibility for the inferred mechanisms. For example, several predicted compounds inhibit cyclin-dependent kinases (e.g., CDK7, CDK9) or Aurora kinases, which are known to regulate RNA polymerase II activity, enhancer transcription, and histone phosphorylation. These drugs can therefore exert direct effects on transcriptional and chromatin states potentially linked to the epigenomic reversals observed in our analysis.

Together, these observations suggest that while most predicted drugs likely act upstream of the epigenome through signaling pathways, epigenomic profiling provides a powerful readout of their downstream regulatory impact. Moreover, the identification of drugs that directly modulate transcriptional or chromatin-regulatory machinery highlights the potential of this framework to generate mechanistic hypotheses about drug action in bipolar disorder–associated immune dysfunction.

We have added a clarification of this assumption under "Drug repurposing analysis" of the Methods section: "In this analysis, epigenomic signatures were used as proxies for downstream transcriptional states, under the assumption that disease-associated changes in histone modifications reflect altered gene regulatory programs that can be reversed by pharmacological perturbations, analogous to expression-based drug repurposing frameworks such as CMap". We have also added the following explanation to the Discussion: "Unlike direct target–based approaches, this regulatory reversal–based strategy prioritizes compounds that counteract disease-associated gene programs inferred from epigenomic changes. For compounds that directly target epigenetic or transcriptional regulators, e.g., CDK7, CDK9, or Aurora kinases (**Fig. 6c),** this approach may also help elucidate mechanisms of action by revealing their effects on regulatory pathways."

• Was there evidence that in patients in their cohort, antipsychotics reversed the BD GWAS-colocalizing gCREs or dCREs?

Thank you for the comment. We investigated whether BD-associated epigenomic signals, specifically dCREs and BD GWAS-colocalized gCREs, could be reversed by antipsychotic or mood-stabilizing drugs identified as impacting immune function (Fig. 6a). To do so, we tested whether the effect sizes of BD vs. control correlated with drug-induced changes based on our differential analysis.

We observed significant anti-correlations between drug responses and BD effects across multiple histone marks:

Aripiprazole: anti-correlated with BD effect sizes on H3K36me3 dCREs ($P=3.2\times 10^{-66}$) and H3K27me3 dCREs ($P=7.2\times 10^{-10}$);

Topiramate: anti-correlated with BD-colocalizing H3K27me3 gCREs (P = 0.016);

Trazodone: anti-correlated with BD effects on H3K4me3 dCREs ($P=2.4\times 10^{-18}$) and BD-colocolized gCREs ($P=0.058$), H3K27ac dCREs ($P<2.2\times 10^{-308}$) and BD-colocolized gCREs ($P=1.5\times 10^{-6}$), H3K27me3 dCREs ($P=1\times 10^{-8}$) and BD-colocolized gCREs ($P=0.014$);

Gabapentin: strongly anti-correlated with BD effects on H3K4me3 dCREs ($P=5.3\times 10^{-41}$) and BD-colocolized gCREs ($P=0.0087$), H3K27ac dCREs ($P<2.2\times 10^{-308}$) and BD-colocolized gCREs ($P=4.9\times 10^{-6}$), H3K36me3 dCREs ($P=3\times 10^{-138}$);

Divalproex: anti-correlated with H3K4me1 dCREs (*P* = 0.015) and colocalizing gCREs (*P* = 0.017).

As an example, we highlight Gabapentin's reversal of BD-associated H3K27ac signals (**Response Fig. 13**). These results suggest that BD-relevant drugs may reverse disease-associated epigenomic alterations in a drug- and histone mark–specific manner.

We have added the following to the main text: "We also found that these drugs may reverse the epigenomic impact associated with BD in a histone mark–dependent manner. For example, Gabapentin anti-correlates with BD effects on H3K27ac dCREs (P $<2.2\times 10^{-308}$) and BD-colocalizing gCREs (P = 4.9×10^{-6}) (**Supplementary Fig. 6a**)."

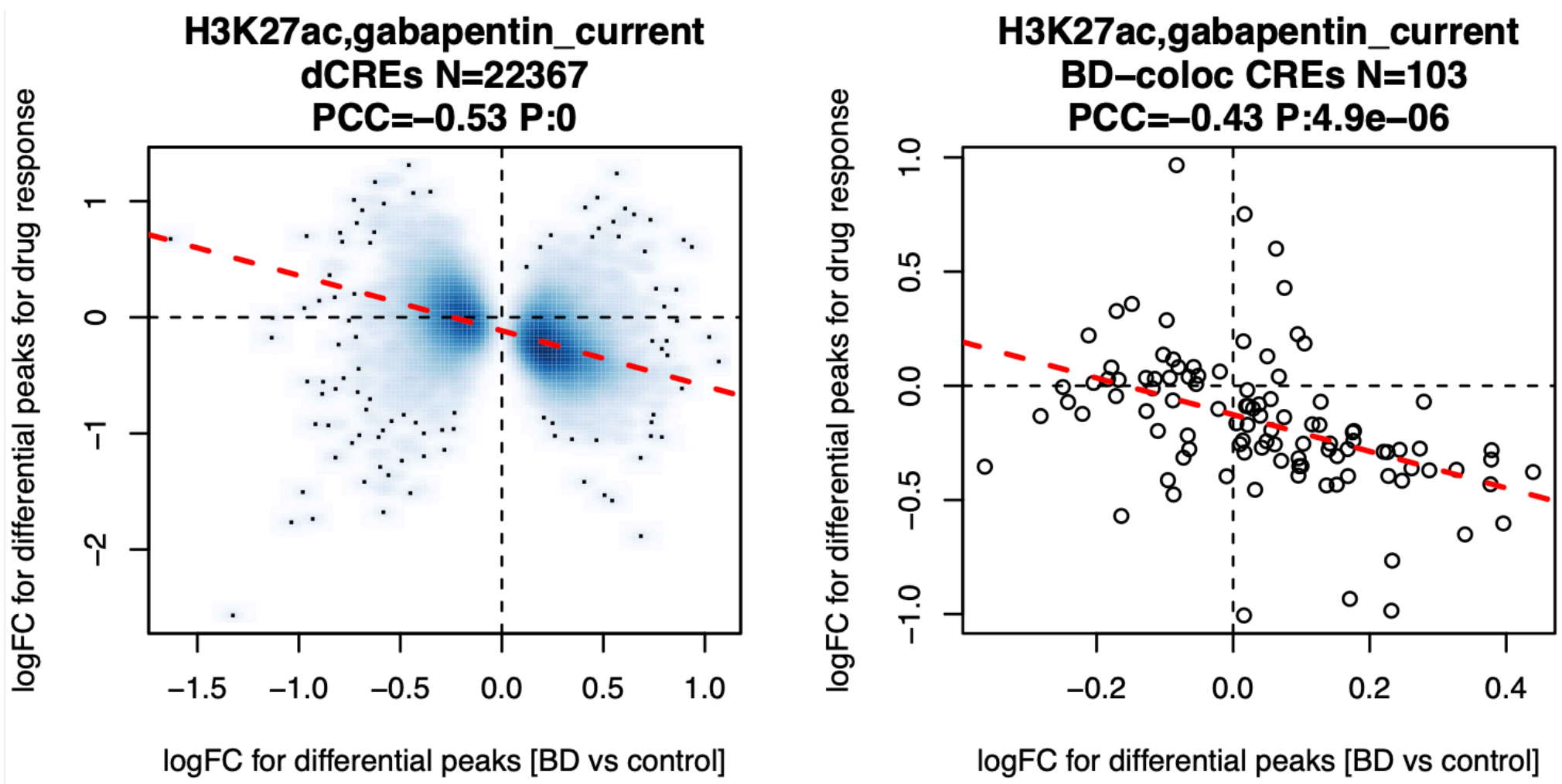

Response Fig. 13. Gabapentin reverses the differential activity of H3K27ac CREs associated with BD.

• Can the authors highlight the drugs prioritised as most likely to worsen BD? Discussion of flutamide exacerbating mania in discussion, but CMap-prioritised drugs predicted to worsen BD would be good to highlight in results.

We indeed noticed that some of the suggested drugs might lead to detrimental, rather than beneficial effectseffect onto BD. For example, flutamide may induce manic-like episodes[130] (**Supplementary Fig. 6e**) and nicotine could exacerbate manic episodes in individuals with BD[131] (**Supplementary Fig. 6f**). We have added this comment to the Discussion.

However, our intention is not to use the current analysis to predict drug-induced worsening of BD, as such efforts would require more direct and systematic evidence of adverse psychiatric effects. Instead, we view the epigenomic signature-based approach as a means to explore the potential immune impact of pharmacological agents, highlighting how certain drugs might modulate or even reverse immune pathways dysregulated in BD. This framework may ultimately aid in understanding mechanisms of action or off-target immune effects rather than serving as a predictive tool for clinical risk of drug misuse.

• Supplementary Figure 2d – within the network the edge width looks to represent a data feature, but this is not defined.

Great catch! We have added “edge width indicates the strength of the functional association” in the figure legend.

Reviewer #4 (Remarks to the Author):

I co-reviewed this manuscript with one of the reviewers who provided the listed reports. This is part of the Nature Communications initiative to facilitate training in peer review and to provide appropriate recognition for Early Career Researchers who co-review manuscripts.

Reviewer #5 (Remarks to the Author):

The study is well-designed, and the manuscript is well-crafted. Authors profiled histone modification in Type I bipolar disorder patients and controls and mapped hQTLs. By integrating dCREs, gCREs, and CRE–gene links, they identified disease driver genes and grouped cases by epigenomic profiles. Additionally, they discussed the potential for drug repurposing based on expression signatures in the Connectivity Map. Throughout their analyses, they revealed the important role of immune related genes and pathways in disease pathogenesis. The limitations of this study were thoroughly discussed, particularly the lack of paired brain samples and gene expression data.
We appreciate the reviewers' positive comments!

I have the following minor comments:
1. The study cohort includes 88 Type I BD patients, not Type II BD. Type I and Type II BD are different in terms of genetics, epigenetics and clinical manifestations. I suggest authors to tone done – state that the findings only apply to Type I BD, not BD.
Thank you for this important point. We agree that type I and type II bipolar disorder have distinct clinical and biological characteristics. To clarify this in the manuscript, we now explicitly state in the Abstract, Introduction, beginning of main text (Results), Discussion, and Methods that our cohort includes only **individuals diagnosed with type I BD**. Specifically, We also added "in type I BD (referred as BD in the rest of results)" at the beginning of the Results.

To avoid repetition, we use "BD" throughout the manuscript for readability, but the scope of our findings should be interpreted as referring specifically to **type I BD**, unless otherwise noted. We have also added clarifying language in the limitations sections of discussion "Finally, our study cohort consists primarily of non-Hispanic White individuals diagnosed with Type I bipolar disorder. While this enhances the accuracy of PRS-based analyses and reduces heterogeneity within the cohort, it also limits the generalizability of our findings to other ancestral populations and to individuals with type II BD."

2. Italic gene names, e.g. page 1, line 32, ORMDL3; page 5, line 20-26.
Thanks for the suggestion! We went through the whole manuscript and fixed all of them.

3. Page 2, line 8, 9: please cite the most recent GWAS:
depression: https://www.cell.com/cell/fulltext/S0092-8674(24)01415-6;
bipolar: https://doi.org/10.1038/s41586-024-08468-9
Thanks for the reminder! We have updated the citations.

4. Results – section 1: Authors discussed TF motif and GO enrichment analyses results for G3 and G12, but did not mention other groups. Even if the results were not promising, I suggest to briefly discuss, instead of cherry picking the positive ones.

We thank the reviewer for the thoughtful suggestion. In our main text, we highlighted G3 and G12 as examples to illustrate the immune- and neuron-related specificity of CRE modules, respectively. Many of the broadly active groups (e.g., G1 and G2) were enriched for general transcription machinery or housekeeping functions, which were less informative in the context of tissue-specific regulatory roles and thus were not emphasized initially. Other groups with mixed or weakly tissue-enriched activity showed either limited enrichment or results that were difficult to interpret biologically. Nevertheless, we agree it is necessary to briefly acknowledge these outcomes to provide a full picture of our CRE module characterization, especially for CRE groups that are later associated with significantly difference epi-partitioned PRSs between BD and control, including broadly-active, multi-tissue, and placenta. We have added a sentence in the Results to reflect this in the revised manuscript "Additionally, CREs broadly active across tissues (G1) were enriched for terms such as *mRNA metabolic processes*, multi-tissue CREs (G2) for *negative regulation of apoptotic processes*, and placenta-predominant CREs (G5) for *cell migration and developmental regulation*. Together, these results highlighted that CREs detected in blood may originate from distinct regulatory programs across diverse tissue contexts."

5. Typo: page 4, line 29: adjust P 0.05.

Thanks for the comments. Changed to "adjusted *P*", and also fixed several other typos.

6. Authors set PP4>0.5 as the cutoff in coloc analysis (page 6, line 20; page 7, line 38). This is too liberal. Usually, PP4>0.7 or 0.8 is used.

Thank you for pointing this out. We agree that 0.5 is permissive, and stringent cutoffs (0.7, 0.8) could lead to higher-confident hits. The main reason for applying 0.5 as a cutoff in the manuscript was to allow broader alignment across different histone marks, between blood and brain, and between eQTL and hQTL in the downstream analyses.

We applied PP4>0.7, resulting in 196 merged CREs hits across marks, enriching similar terms such as "negative regulation of neurotrophin TRK receptor signaling pathway", "regulation of replicative cell aging", "retrograde vesicle−mediated transport, Golgi to ER", and "caveolin−mediated endocytosis" in Fig. 3c, supporting our main conclusion that colocalized CREs are enriched for **ER-related functions**.

We have now added under GWAS-hQTL, hQTL-eQTL, and GWAS-eQTL colocalization and Mendelian randomization (MR) in the methods section that "We also tried a more stringent cutoff PP4>0.7, leading to consistent enrichment results as in Fig. 3C". We have also included a tab "coloc pp4" in Supplementary Table S4, listing gCREs with PP4 values, so that readers of interest could look for higher-confidence colocalization events using their preferred confidence thresholds.

7. Page 6, line 23: P<= 1e-6 was used for GWAS genome-wide significance cutoff. Is there any reason why not use P<5e-8, which is widely accepted in the field?
Thanks for the comments. While $P < 5\times10^{-8}$ is the widely accepted genome-wide significance threshold for GWAS discovery, previous reports also support that subthreshold signals could also be causal variants[7]. Thus, in integrative analyses that prioritize regulatory variants through QTL colocalization, it is common to use a more suggestive threshold (e.g., $P \leq 1\times10^{-6}$) to capture additional loci with supportive regulatory evidence, particularly when combined with colocalization or MR as downstream analysis. Here, the primary evidence for shared genetic effects between BD risk and histone mark regulation comes from the coloc posterior probability (PP4 > 0.5), which explicitly quantifies the probability of a shared causal variant, rather than just the nominal GWAS P-value alone. By using a suggestive locus threshold together with a PP4 cutoff, we aim to balance sensitivity and specificity in identifying regulatory loci that may underlie BD risk as we applied in prior integrative QTL and colocalization studies[5,6]. We have clarified this rationale in the Methods section by adding the following statement: "To increase sensitivity in identifying potential regulatory variants that colocalize with hQTLs, we applied a subthreshold cutoff for GWAS ($P \leq 1\times10^{-6}$)".

8. Figure legend - figure 3b: What do the red box and black box indicate? What's the significance cutoff? It was mentioned in the main text, but not in the figure legend.
Great catch! We have now added "red box, enrichment of the immune component; black boxes, enrichment of the brain component" and "red boxes, enrichment of the immune component" in the legends of Fig.3b and Ext Data Fig.3e, respectively.

9. Please double check paragraph format throughout the manuscript upon publication, e.g. spacing, especially in the method section.
Thanks for the comments. We fixed the format due to the different fonts misapplied.

10. Figure 4b: background (grey) bars are missing in the figure, and figure 4b was not discussed in the main text.
Thanks for catching it. In Fig. 4b background bars are there, it is very small because the value is very low near to 0. We have added "all eGenes as background (in gray)" to the figure legend. Also, we have discussed Fig.4b as "The driver genes identified via both types of linking evidence showed significant enrichment (one-sided proportion test $P < 1\times10^{-85}$) for the genes associated with BD genetics based on GWAS–eQTL colocalization in blood or brain bulk samples (*coloc* PP4 ≥0.5) (**Fig. 4b**)".

11. Figure 4d: blood-specific and brain-blood shared coloc signals were plotted. I am wondering if there is any brain-specific coloc signal.

Thanks for the comments. Our data is from blood, that's why we focus on interpreting signals coming from blood, either blood-specfic signals or signals shared between the blood and the brain. We found that a small proportion of driver genes were only supported by brain-specific GWAS-eQTL coloc signals as shown in the upper left corner in Fig. 4C, including *MED24*, *VPS45*, *FADS2*, *GLT8D1*, which may be due to the misalignment of different linking strategies between eQTL, HiC linking, and our gLink scores.

Reference

1. Leek, J. T. & Storey, J. D. Capturing heterogeneity in gene expression studies by surrogate variable analysis. *PLoS Genet* **3**, 1724–1735 (2007).
2. Leek, J. T., Johnson, W. E., Parker, H. S., Jaffe, A. E. & Storey, J. D. The sva package for removing batch effects and other unwanted variation in high-throughput experiments. *Bioinformatics* **28**, 882–883 (2012).
3. Leek, J. T. Asymptotic conditional singular value decomposition for high-dimensional genomic data. *Biometrics* **67**, 344–352 (2011).
4. Gu, Z. & Hübschmann, D. rGREAT: an R/bioconductor package for functional enrichment on genomic regions. *Bioinformatics* **39**, btac745 (2022).
5. Xiong, X. *et al.* Genetic drivers of m6A methylation in human brain, lung, heart and muscle. *Nat. Genet.* (2021).
6. Hou, L. *et al.* Multitissue H3K27ac profiling of GTEx samples links epigenomic variation to disease. *Nature Genetics* **55**, 1665–1676 (2023).
7. Wang, X. *et al.* Discovery and validation of sub-threshold genome-wide association study loci using epigenomic signatures. *Elife* **5**, (2016).

REVIEWER COMMENTS

We thank all the reviewers' positive feedback! Please check our point-to-point response in blue.

Reviewer #1 (Remarks to the Author):

The authors have addressed all remaining concerns

Thank you!

Reviewer #2 (Remarks to the Author):

I co-reviewed this manuscript with one of the reviewers who provided the listed reports. This is part of the Nature Communications initiative to facilitate training in peer review and to provide appropriate recognition for Early Career Researchers who co-review manuscripts.

Reviewer #2 (Remarks on code availability):

I co-reviewed this manuscript with one of the reviewers who provided the listed reports. This is part of the Nature Communications initiative to facilitate training in peer review and to provide appropriate recognition for Early Career Researchers who co-review manuscripts.

Reviewer #3 (Remarks to the Author):

We thank the authors for their comprehensive responses to our queries. The revised paper is in our view strong and of significant novelty and interest. Most of our concerns are fully addressed.

Thank you!

Two outstanding issues:

Batch effects - cases/controls are largely in different batches as the authors describe. Their rebuttal attempts to address this with permutation testing (but this is more a reflection of whether this signal is just random noise not intrinsic technical variation) and then differential testing within group vs between groups. From our understanding, since cases/controls were batched separately this could be highlighting batch effects not case-control differences. The permutation testing would seem to confirm that not all of the signal is driven by batch. The authors then perform PCA and label based on batch, but this plot is not shown. Please can this plot be shown, and can the authors ensure the text reflects the degree to which the effects may be in part driven by batch.

Thanks for this important comment! We have now added the PCA plot for residuals as **Supplementary Figure 4c**. And refined the statement of batch effects in methods, "these analyses suggested that batch effects might still impact differential results, however, were unlikely to be the main driver of the observed BD vs. control differences."

Inter-individual variability – the authors now show peak presence across samples for H3K27ac

(Response Fig 10). Please can the authors add histograms separated by case/control status and include all 5 marks as supplementary figures. We think this variability is important to report so readers can assess which marks are less variable between individuals within group.

Thank you for this helpful suggestion. We have now included histograms of peak presence across individuals separated by case and control status for all five histone marks as **Supplementary Figure 2** (also shown below). These plots allow readers to assess variability across individuals for each mark.

Based on these results, we observed a distribution shift for H3K4me1, while only a limited subset of peaks showed differential presence between cases and controls (third column), suggesting that differences between groups may exist. However, peak presence can also be strongly influenced by various confounding factors. To more robustly capture variability across samples, we therefore performed statistical differential analyses, as described in the following sections. Nevertheless, we agree that presenting these distributions is important for transparency and allows readers to evaluate variability across histone marks.

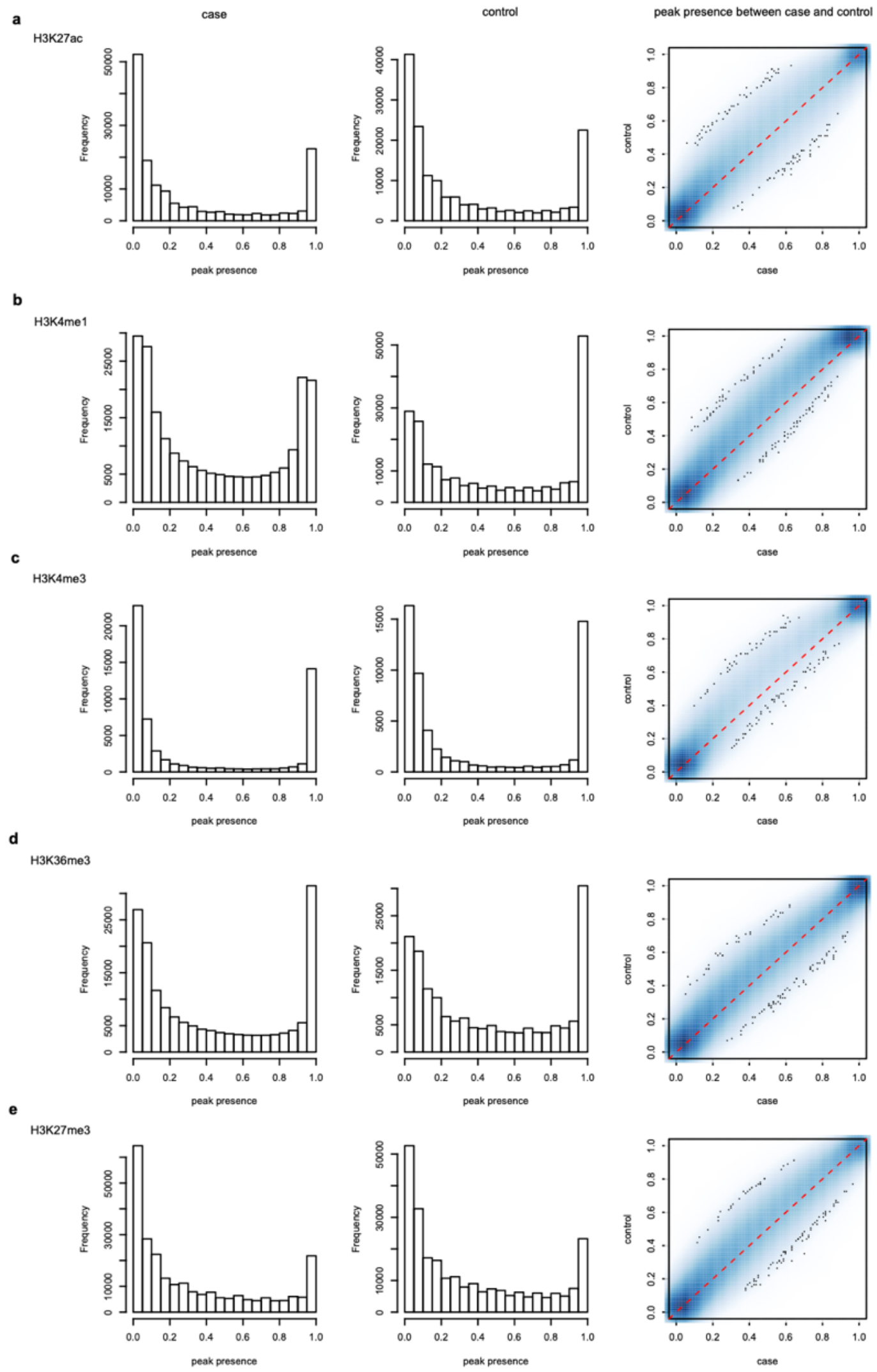

Reviewer #4 (Remarks to the Author):

I co-reviewed this manuscript with one of the reviewers who provided the listed reports. This is part of the Nature Communications initiative to facilitate training in peer review and to provide appropriate recognition for Early Career Researchers who co-review manuscripts.

Reviewer #5 (Remarks to the Author):

Thanks for the authors to anwser and reply my comments and questions. They are fully addressed.

Thank you!
