# Supplementary material for: Genotype epigenome phenotype integration reveals peripheral immune contributions to type I bipolar disorder
Source: Nat Commun. 2026 May 15;17:7740. doi: 10.1038/s41467-026-72543-0 (PMC13434788; doi:10.1038/s41467-026-72543-0)
Supplement: Supplementary file 3 [file 41467_2026_72543_MOESM1_ESM.pdf]

Genotype Epigenome Phenotype Integration Reveals Peripheral Immune Contributions to Type I Bipolar Disorder

Supplementary Figures

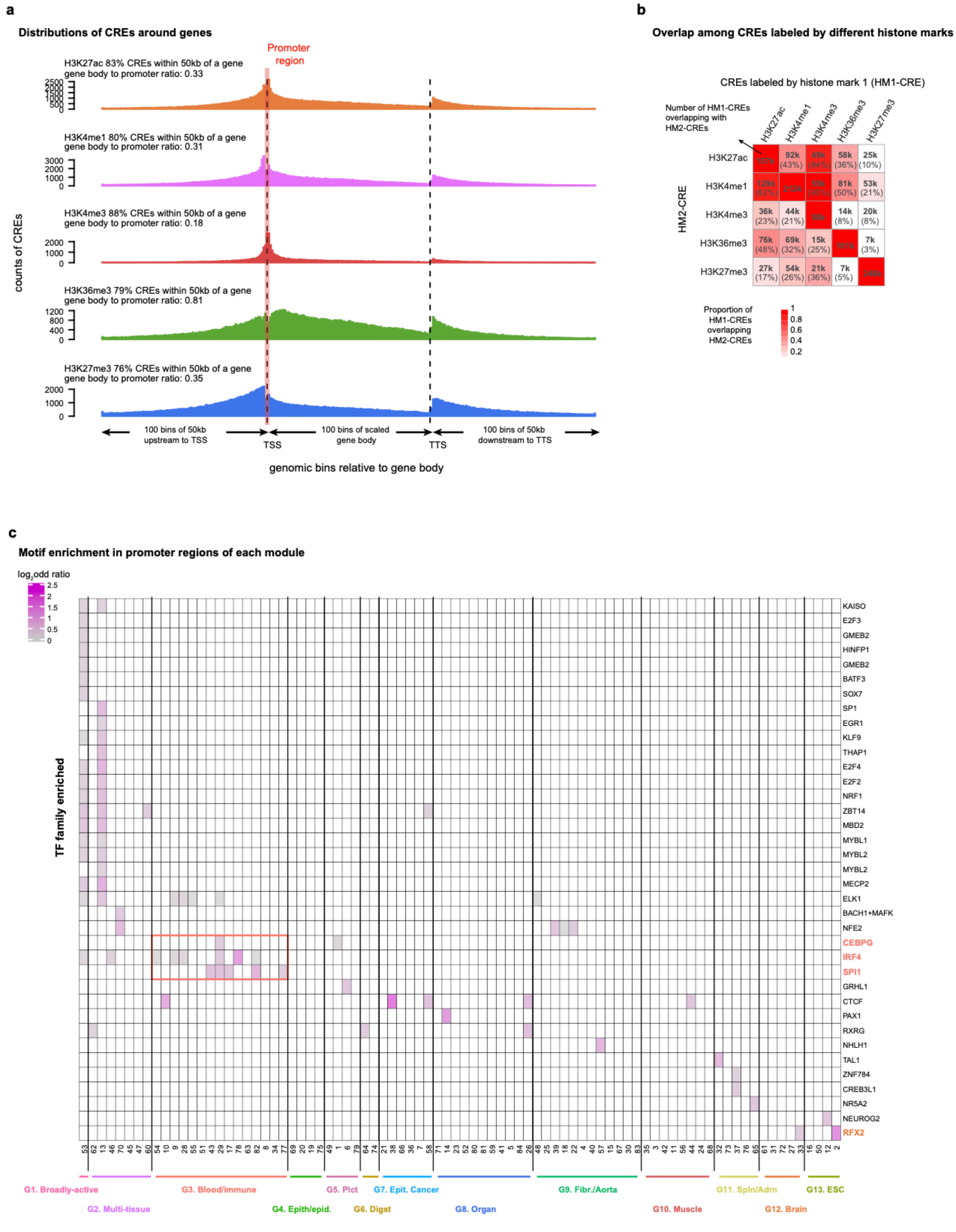
a
Distributions of CREs around genes
Promoter region
H3K27ac 83% CREs within 50kb of a gene
gene body to promoter ratio: 0.33
H3K4me1 80% CREs within 50kb of a gene
gene body to promoter ratio: 0.31
H3K4me3 88% CREs within 50kb of a gene
gene body to promoter ratio: 0.18
H3K36me3 79% CREs within 50kb of a gene
gene body to promoter ratio: 0.81
H3K27me3 76% CREs within 50kb of a gene
gene body to promoter ratio: 0.35
counts of CREs
100 bins of 50kb upstream to TSS
TSS
100 bins of scaled gene body
TTS
100 bins of 50kb downstream to TTS
genomic bins relative to gene body
b
Overlap among CREs labeled by different histone marks
CREs labeled by histone mark 1 (HM1-CRE)
Number of HM1-CREs overlapping with HM2-CREs
H3K27ac
H3K4me1
H3K4me3
H3K36me3
H3K27me3
HM2-CRE
92k (43%)
58k (36%)
25k (10%)
81k (50%)
53k (21%)
36k (23%)
44k (21%)
14k (8%)
20k (8%)
76k (48%)
69k (32%)
15k (25%)
7k (3%)
27k (17%)
54k (26%)
21k (36%)
7k (5%)
Proportion of HM1-CREs overlapping HM2-CREs
1
0.8
0.6
0.4
0.2
c
Motif enrichment in promoter regions of each module
log2odd ratio
2.5
2
1.5
1
0.5
0
TF family enriched
KAISO
E2F3
GMEB2
HINFP1
GMEB2
BATF3
SOX7
SP1
EGR1
KLF9
THAP1
E2F4
E2F2
NRF1
ZBT14
MBD2
MYBL1
MYBL2
MYBL2
MECP2
ELK1
BACH1+MAFK
NFE2
CEBPG
IRF4
SPI1
GRHL1
CTCF
PAX1
RXRG
NHLH1
TAL1
ZNF784
CREB3L1
NR5A2
NEUROG2
RFX2
G1. Broadly-active
G2. Multi-tissue
G3. Blood/immune
G4. Epith/epid.
G5. Plct
G6. Digst
G7. Epit. Cancer
G8. Organ
G9. Fibr./Aorta
G10. Muscle
G11. Spln/Adrn
G12. Brain
G13. ESC

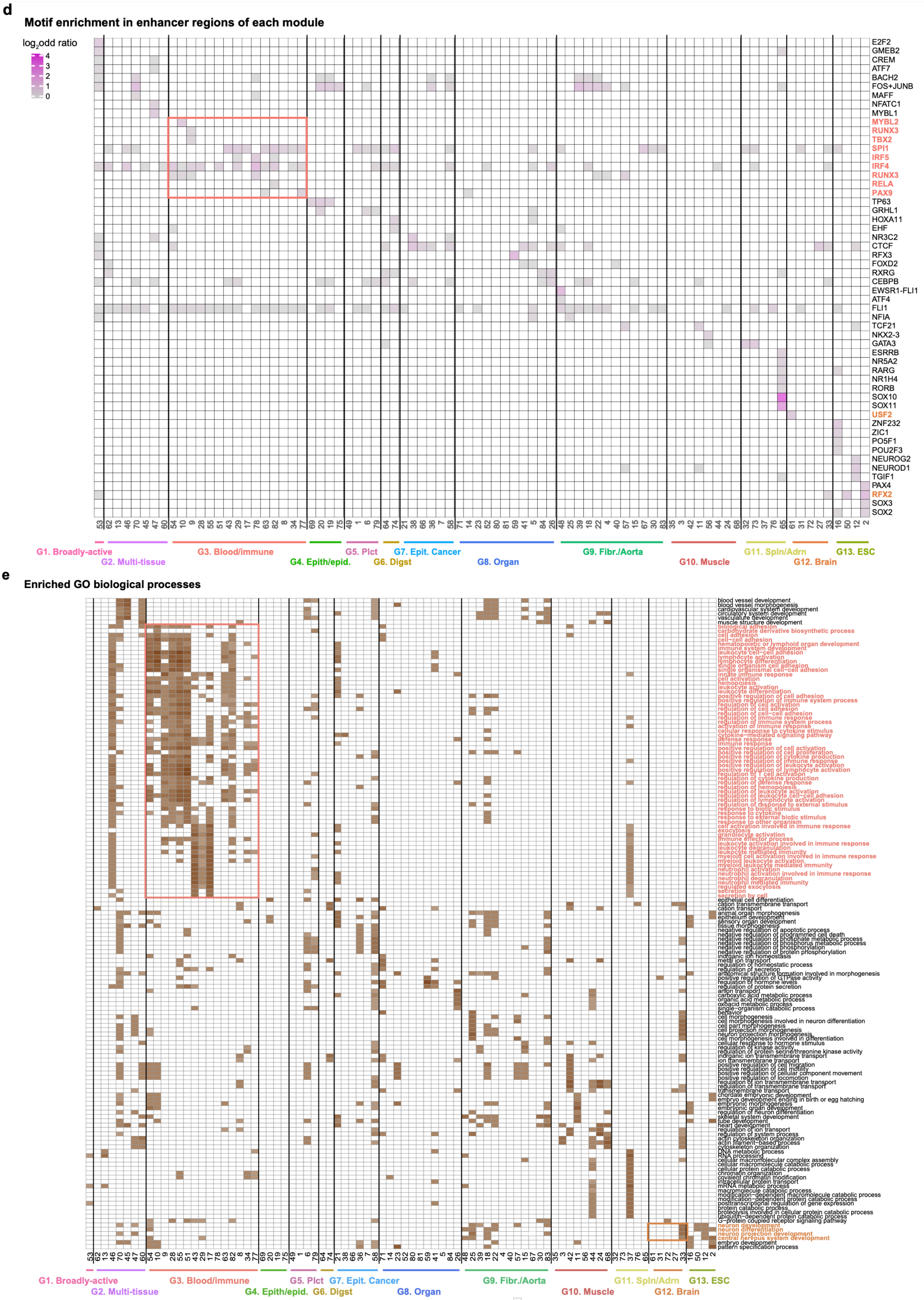
d
Motif enrichment in enhancer regions of each module
log2odd ratio
4
3
2
1
0
E2F2
GMEB2
CREM
ATF7
BACH2
FOS+JUNB
MAFF
NFATC1
MYBL1
MYBL2
RUNX3
TBX2
SPI1
IRF5
IRF4
RUNX3
RELA
PAX9
TP63
GRHL1
HOXA11
EHF
NR3C2
CTCF
RFX3
FOXD2
RXRG
CEBPB
EWSR1-FLI1
ATF4
FLI1
NFIA
TCF21
NKX2-3
GATA3
ESRRB
NR5A2
RARG
NR1H4
RORB
SOX10
SOX11
USF2
ZNF232
ZIC1
PO5F1
POU2F3
NEUROG2
NEUROD1
TGIF1
PAX4
RFX2
SOX3
SOX2
G1. Broadly-active
G2. Multi-tissue
G3. Blood/immune
G4. Epith/epid.
G5. Plct
G6. Digst
G7. Epit. Cancer
G8. Organ
G9. Fibr./Aorta
G10. Muscle
G11. Spln/Adrn
G12. Brain
G13. ESC
e
Enriched GO biological processes
G1. Broadly-active
G2. Multi-tissue
G3. Blood/immune
G4. Epith/epid.
G5. Plct
G6. Digst
G7. Epit. Cancer
G8. Organ
G9. Fibr./Aorta
G10. Muscle
G11. Spln/Adrn
G12. Brain
G13. ESC

Supplementary Figure 1. Epigenomic signals and CRE modules. a Distributions of CREs around genes. The bar plot shows the counts of CREs (y-axis) at each genomic bin (x-axis) upstream of the TSS, between the TSS and the TES, or downstream of the TES for different histone marks. **b** Overlap among CREs labeled by different histone marks. The heatmap shows the proportion of CREs associated with the first histone mark (by column) and the overlapping CREs associated with the second histone mark (by row). Both numbers and proportions of overlapping signals are shown in each cell. **c** Motif enrichment in promoter regions of each module. The heatmap shows the enrichment ($\log_2$ odds ratio) of TF motifs (by row) in CREs in promoter regions for each module defined in **Fig. 1b** (by row)**,** with the TFs enriched in immune modules highlighted. **d** Motif enrichment in enhancer regions of each module. Same as panel b except for showing CREs related to enhancers. **e,** Enriched GO biological processes. The heatmap shows the enrichment ($\log_2$ fold enrichment) of GO terms (by row) for each CRE module. Source data are provided as a Source Data file.

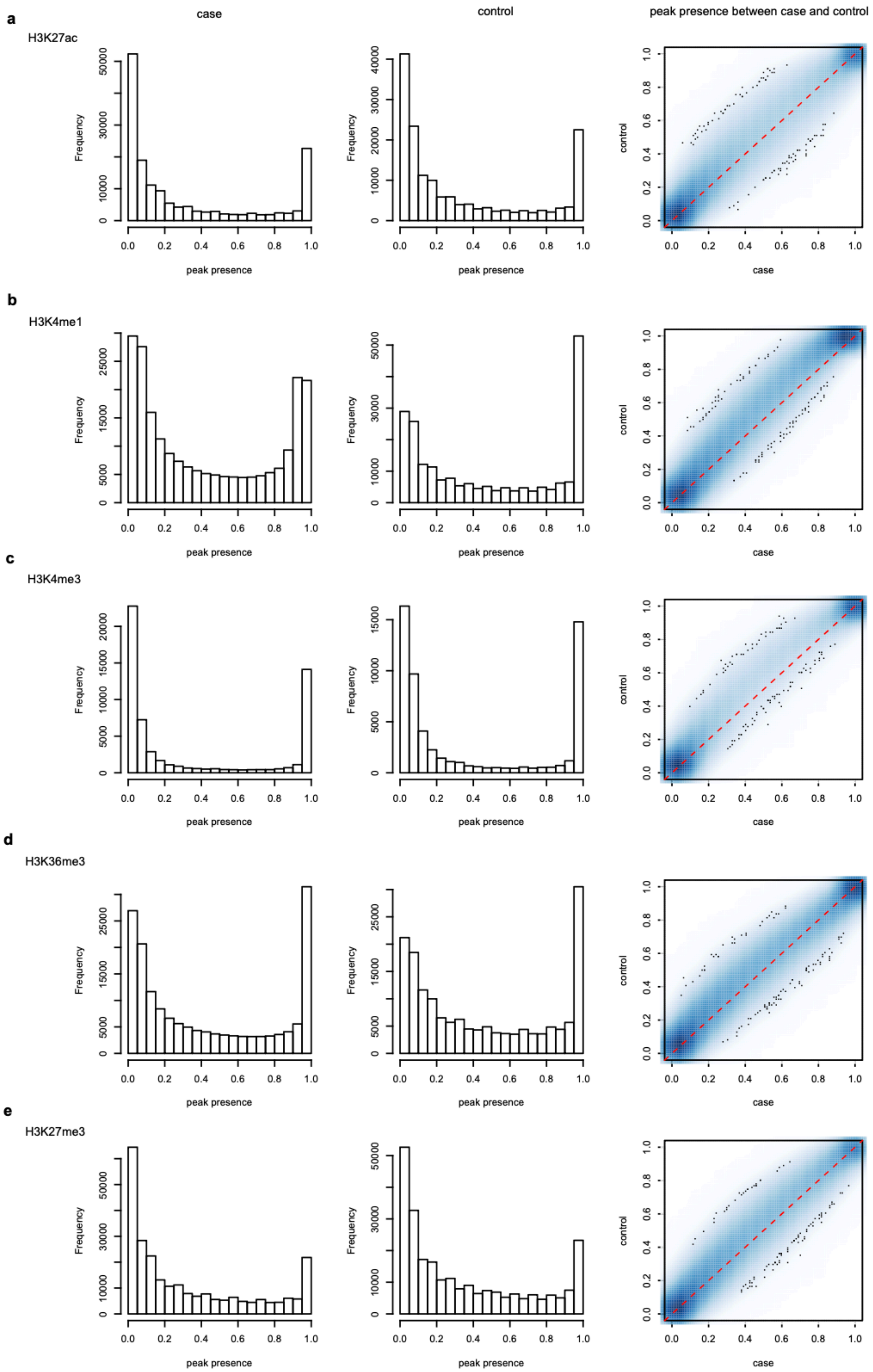

a
b
c
d
e
case
control
peak presence between case and control
H3K27ac
H3K4me1
H3K4me3
H3K36me3
H3K27me3
Frequency
peak presence
control
case

Supplementary Figure 2. Peak detection across individuals and between case/control groups. The bar plots show the distribution of peak presence, defined as the proportion of individuals with a certain peak detected, in case (the first column) and control (the second column), across histone marks (H3K27ac, H3K4me1, H3K4me3m, H3K36me3, and H3K27me3, in panel **a-e**, respectively). Scatter plots in the third column show case-control comparisons of peak presence for each histone marks. Source data are provided as a Source Data file.

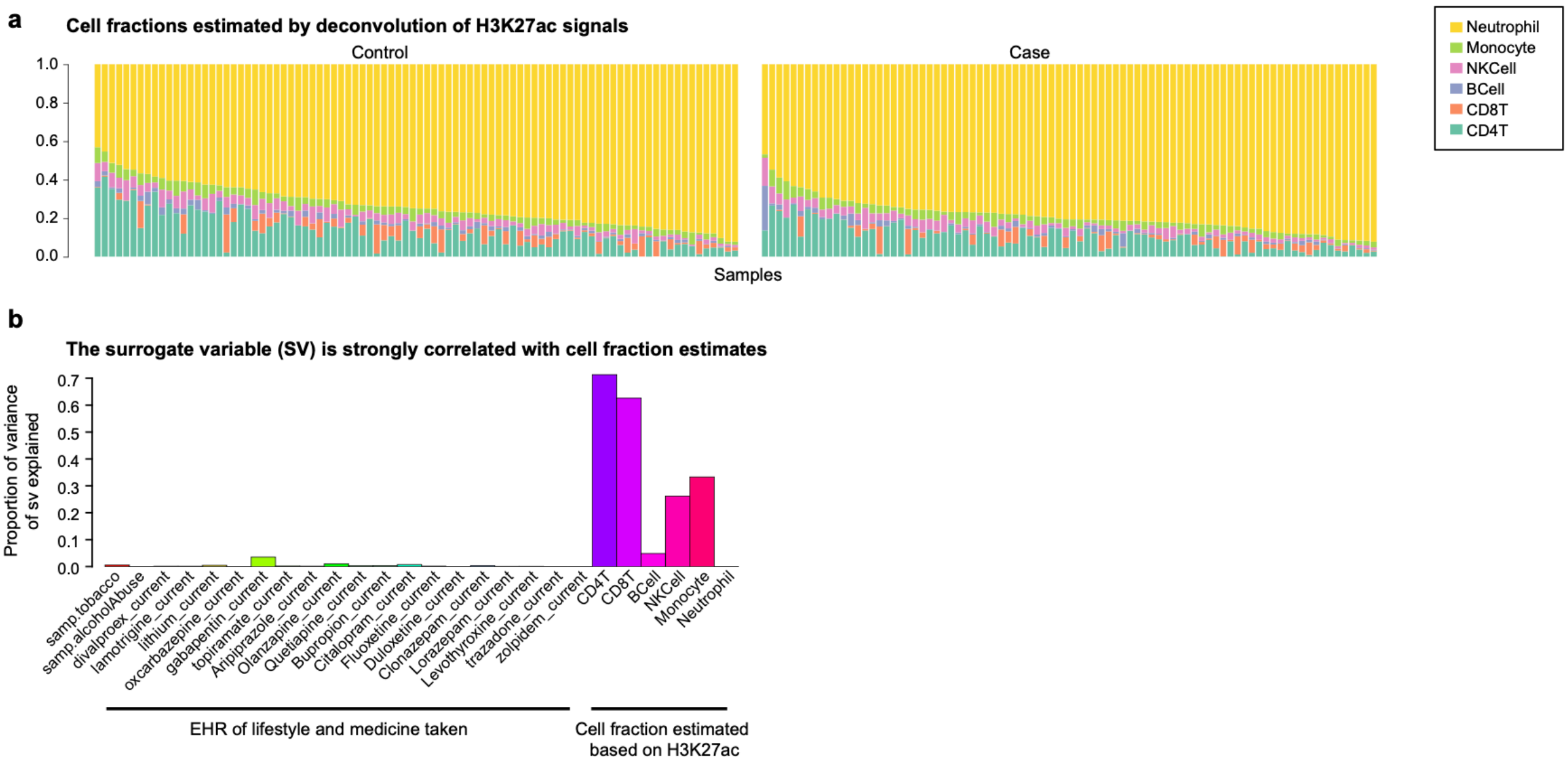

Supplementary Figure 3. Cell fraction estimation. a Cell fractions estimated by deconvolution of the H3K27ac signal. The bar plots show the estimated cell fraction (y-axis) for controls and patients with different cell types in different colors. **b** The surrogate variable (SV) is strongly correlated with the cell fraction estimates. The bar plot shows the proportion of variance of SV explained (y-axis) by the different covariates (x-axis). Source data are provided as a Source Data file.

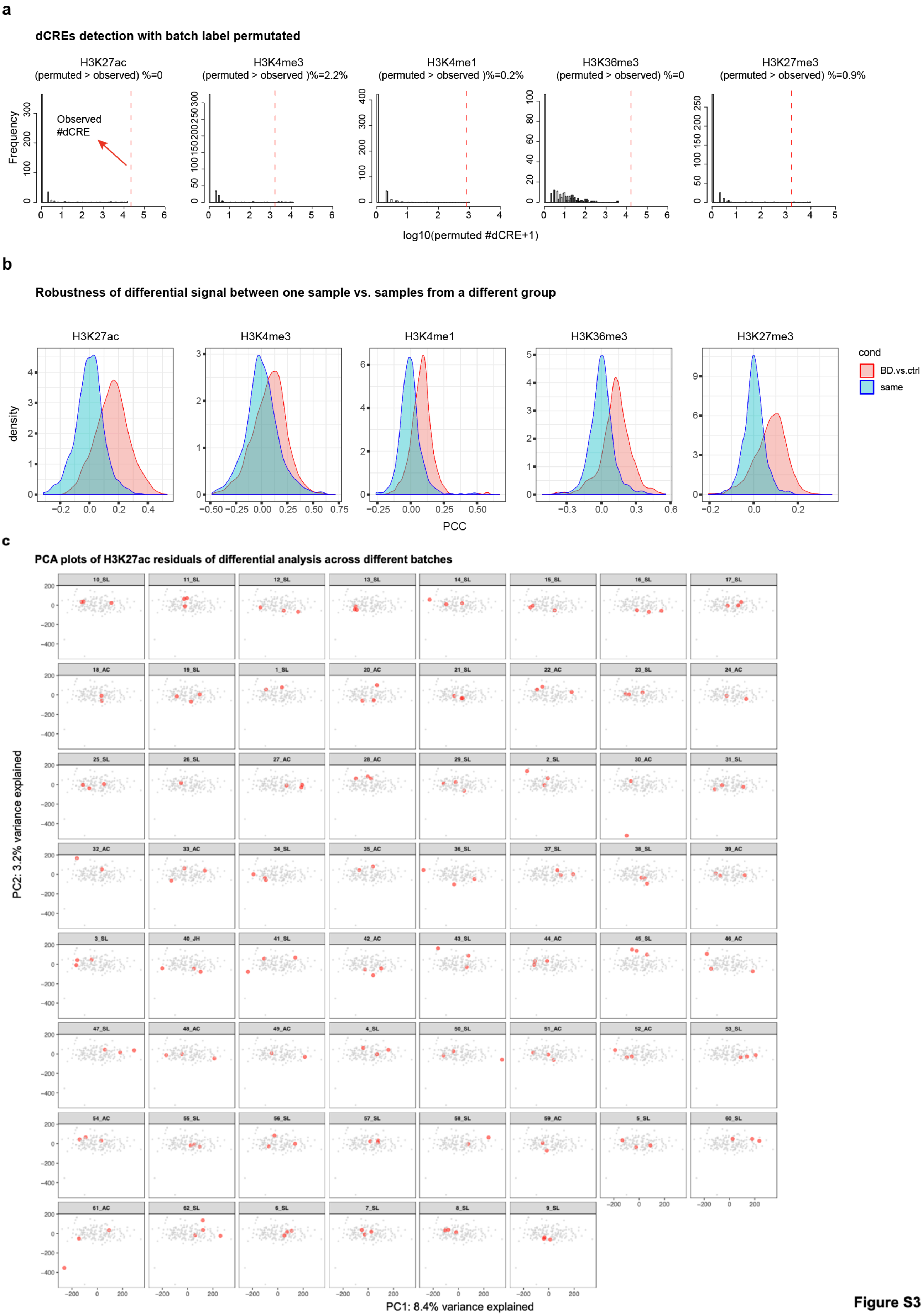
a
dCREs detection with batch label permutated
H3K27ac
(permuted > observed) %=0
H3K4me3
(permuted > observed)%=2.2%
H3K4me1
(permuted > observed)%=0.2%
H3K36me3
(permuted > observed) %=0
H3K27me3
(permuted > observed) %=0.9%
Frequency
Observed
#dCRE
log10(permuted #dCRE+1)
b
Robustness of differential signal between one sample vs. samples from a different group
H3K27ac
H3K4me3
H3K4me1
H3K36me3
H3K27me3
density
PCC
cond
BD.vs.ctrl
same
c
PCA plots of H3K27ac residuals of differential analysis across different batches
PC2: 3.2% variance explained
PC1: 8.4% variance explained

Figure S3

Supplementary Figure 4. Batch effects in differential CREs analysis. a dCREs detection with batch label permutated. The barplots show the distribution of the number dCREs detected under the permutation of batch labels for each histone mark**.** Red dashed lines represent the observed number of dCREs. **b** Robustness of differential signal between one sample vs. samples from a different group**.** Density plots compare the distribution of PCCs of differential activity across CREs under two conditions. "BD vs. ctrl" represents the comparisons between a sample and samples from a different case/control group; "same" represents the comparisons between a sample and others from the same case/control group. **c** PCA plots of H3K27ac residuals of differential analysis across different batches. Each point of the PCA plots represents a H3K27ac profile of a sample, each panel represents a batch, red circles highlight samples from the corresponding batch. Source data are provided as a Source Data file.

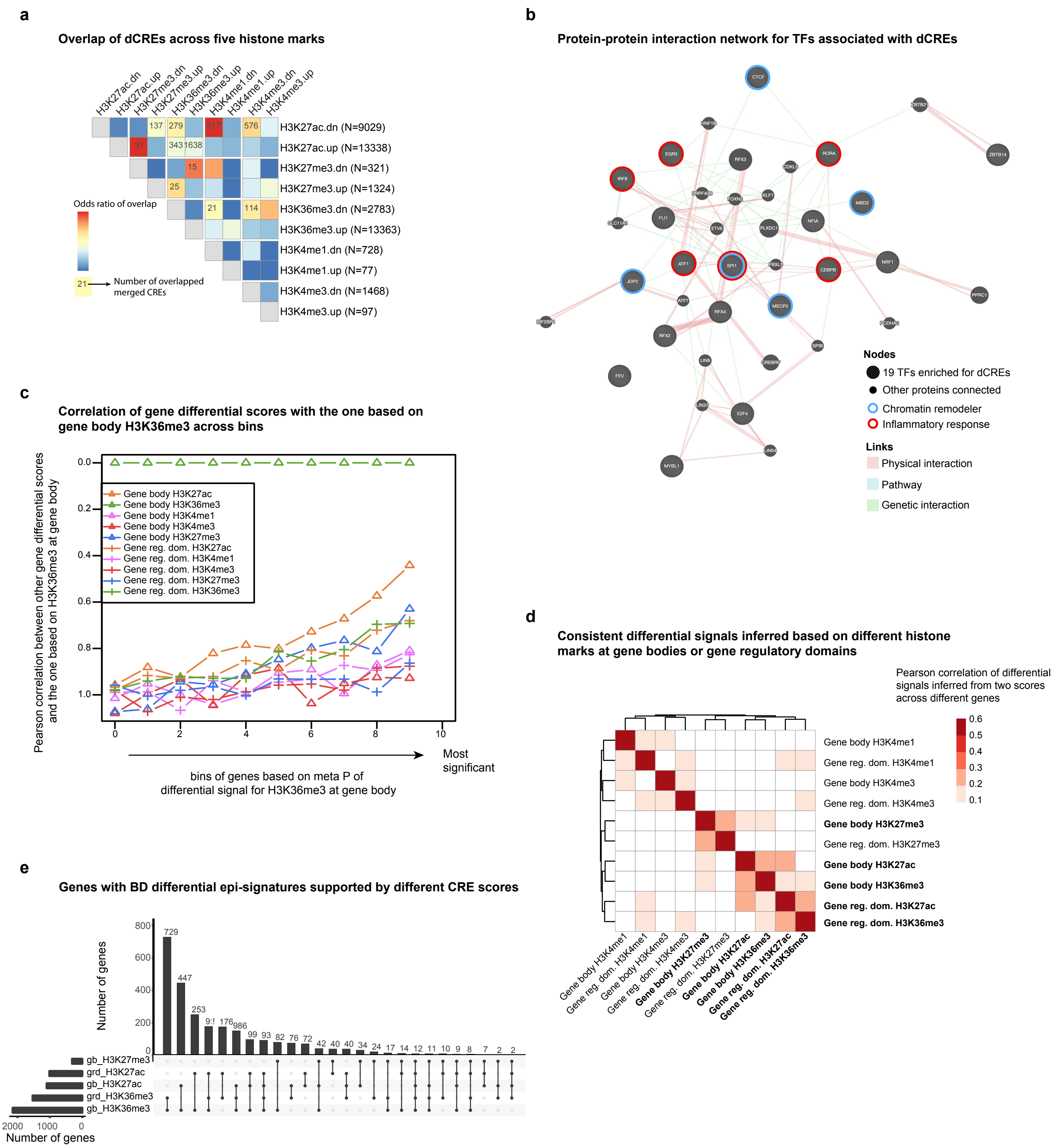
a
Overlap of dCREs across five histone marks
H3K27ac.dn
H3K27ac.up
H3K27me3.dn
H3K27me3.up
H3K36me3.dn
H3K36me3.up
H3K4me1.dn
H3K4me1.up
H3K4me3.dn
H3K4me3.up
H3K27ac.dn (N=9029)
H3K27ac.up (N=13338)
H3K27me3.dn (N=321)
H3K27me3.up (N=1324)
H3K36me3.dn (N=2783)
H3K36me3.up (N=13363)
H3K4me1.dn (N=728)
H3K4me1.up (N=77)
H3K4me3.dn (N=1468)
H3K4me3.up (N=97)
Odds ratio of overlap
21 Number of overlapped merged CREs
b
Protein-protein interaction network for TFs associated with dCREs
Nodes
19 TFs enriched for dCREs
Other proteins connected
Chromatin remodeler
Inflammatory response
Links
Physical interaction
Pathway
Genetic interaction
c
Correlation of gene differential scores with the one based on gene body H3K36me3 across bins
Pearson correlation between other gene differential scores and the one based on H3K36me3 at gene body
Gene body H3K27ac
Gene body H3K36me3
Gene body H3K4me1
Gene body H3K4me3
Gene body H3K27me3
Gene reg. dom. H3K27ac
Gene reg. dom. H3K4me1
Gene reg. dom. H3K4me3
Gene reg. dom. H3K27me3
Gene reg. dom. H3K36me3
Most significant
bins of genes based on meta P of differential signal for H3K36me3 at gene body
d
Consistent differential signals inferred based on different histone marks at gene bodies or gene regulatory domains
Pearson correlation of differential signals inferred from two scores across different genes
Gene body H3K4me1
Gene reg. dom. H3K4me1
Gene body H3K4me3
Gene reg. dom. H3K4me3
Gene body H3K27me3
Gene reg. dom. H3K27me3
Gene body H3K27ac
Gene body H3K36me3
Gene reg. dom. H3K27ac
Gene reg. dom. H3K36me3
e
Genes with BD differential epi-signatures supported by different CRE scores
Number of genes
gb_H3K27me3
grd_H3K27ac
gb_H3K27ac
grd_H3K36me3
gb_H3K36me3
Number of genes

Supplementary Figure 5. Differential CREs and associated TFs. a Overlap of dCREs across five histone marks. The heatmap shows the odds ratio of overlap between each pair of BD dCRE sets with the size of overlapping dCREs shown in the cell if significant (Fisher's exact test, two-sided, BH multiple test correction, adjusted P<0.05). **b** Protein-protein interaction network for TFs associated with dCREs. The network shows linking (edge width indicates the strength of the functional association) among the TFs associated with BD dCRE sets (big, borders in different colors indicating different functions) and other interacting proteins (small in size). **c** Correlation of gene differential scores with the one based on gene body H3K36me3 across bins. The scatterplot shows the correlation (y-axis) between gene body H3K36me3 score (z score) with each of the remaining scores for each bin of genes based on the significance of gene body H3K36me3 score ($-\log_{10} P$ based on a meta-analysis of dCREs, two-sided). **d** Consistent differential signals inferred based on different histone marks at gene bodies or gene regulatory domains. The heatmap shows the correlation among scores based on dCRE meta-analysis (by row and by column) included in panel e. **e** Genes with BD differential epi-signatures supported by different CRE scores. The upset plot shows the counts (y-axis) of genes with BD differential epi-signatures supported by different dCRE meta-analysis scores (x-axis). Source data are provided as a Source Data file.

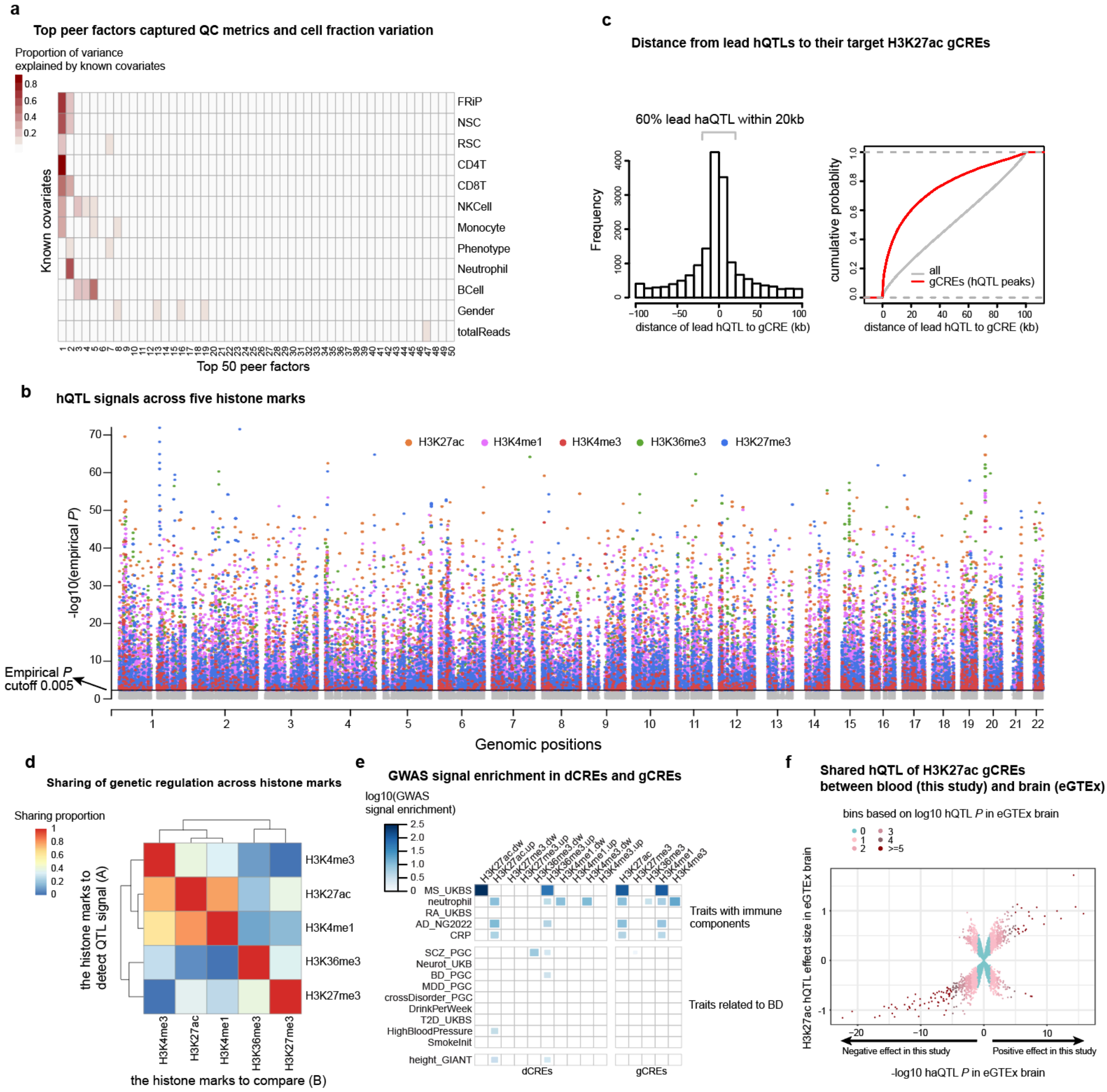

Supplementary Figure 6. Genetic regulation of CREs and implications in BD genetics. a Top PEER factors captured QC metrics and cell fraction variation. The heatmap shows the correlation between PEER factors (by column) and known or inferred covariates (by row) for H3K27ac as an example. **b** hQTL signals across five histone marks. The scatter plot shows the QTL signals (empirical *P*, y-axis) over the genome (x-axis) for five histone marks (in different colors shown on top). **c** Distance from the lead hQTLs to their target H3K27ac gCREs. Left: the barplot shows the frequency of the top hQTLs (y-axis) across the bins of distance between the QTLs and their target gCRE; right: the plot shows the cumulative distributions of distances between the top hQTLs and its target CRE for gCREs (red) and all CREs as background (gray). **d** Sharing of genetic regulation across histone marks. The heatmap shows the sharing of hQTLs detected for histone mark A (by row) with those detected for histone mark B (by column). **e** GWAS signal enrichment in dCREs and gCREs. **f** Comparing genetic regulation of H3K27ac between blood (this study) and brain (eGTEx). X axis: nominal *P* value significance of the hQTLs (two-sided) in the eGTEx brain, separated by positive effect and negative effect of the matched hQTL–CRE pair in our study; Y axis: effect size of hQTL in eGTEx brain. Source data are provided as a Source Data file.

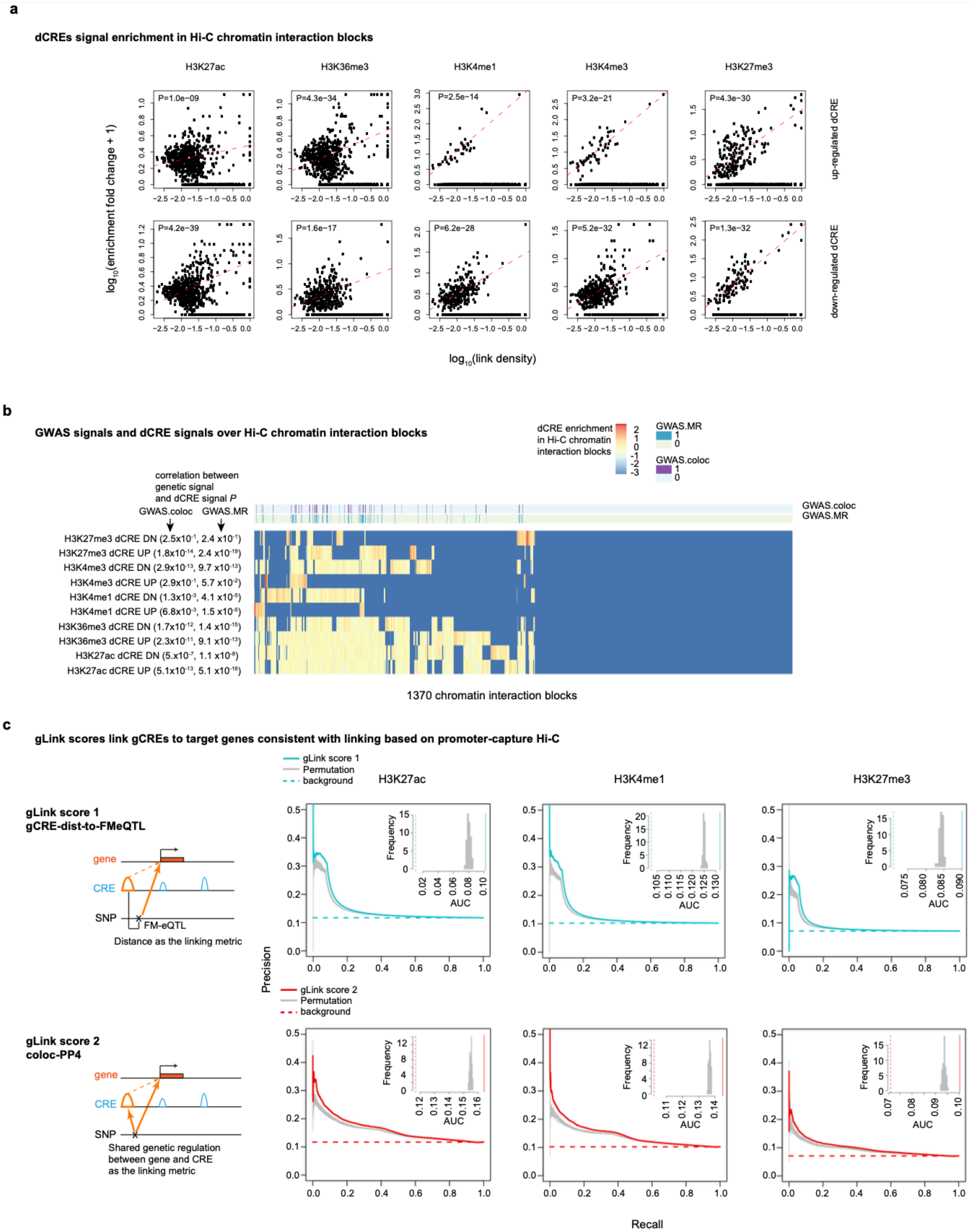

Supplementary Figure 7. Patterns of dCREs at Hi-C block level and comparison between gLink scores and Hi-C links. a dCRE signal enrichment in Hi-C chromatin interaction blocks. The multi-paneled scatter plot compares the enrichment of dCREs to the density of links, defined as the number of observed Hi-C links divided by the number of all pairs of chromatin regions, across each Hi-C block (each point) for up- or down-regulated dCREs (by row) for each histone mark (by column). *P* value of correlation (two-sided) between the enrichment and link density at log scale is

shown. **b** GWAS signals and dCRE signals over Hi-C chromatin interaction blocks. The heatmap shows the enrichment of dCRE sets (by row) in each Hi-C block (by column) with BD genetic signals for each block annotated at the top. Nominal *P* value of correlation (two-sided) between genetic signal and dCRE enrichment across HiC blocks are shown on left. **c** gLink scores link gCREs to target genes consistent with linking based on promoter-capture Hi-C. AUPRC plots show the performance of each gLink score (by row) for each histone mark (by column) with links based on promoter-capture Hi-C data as the benchmark data. In details, genomic regions linked to gene promoters through direct link or 1 step indirect link based on Hi-C were considered as linked CREs. Schematic plots to explain two different gLink scores are shown on the left. Source data are provided as a Source Data file.

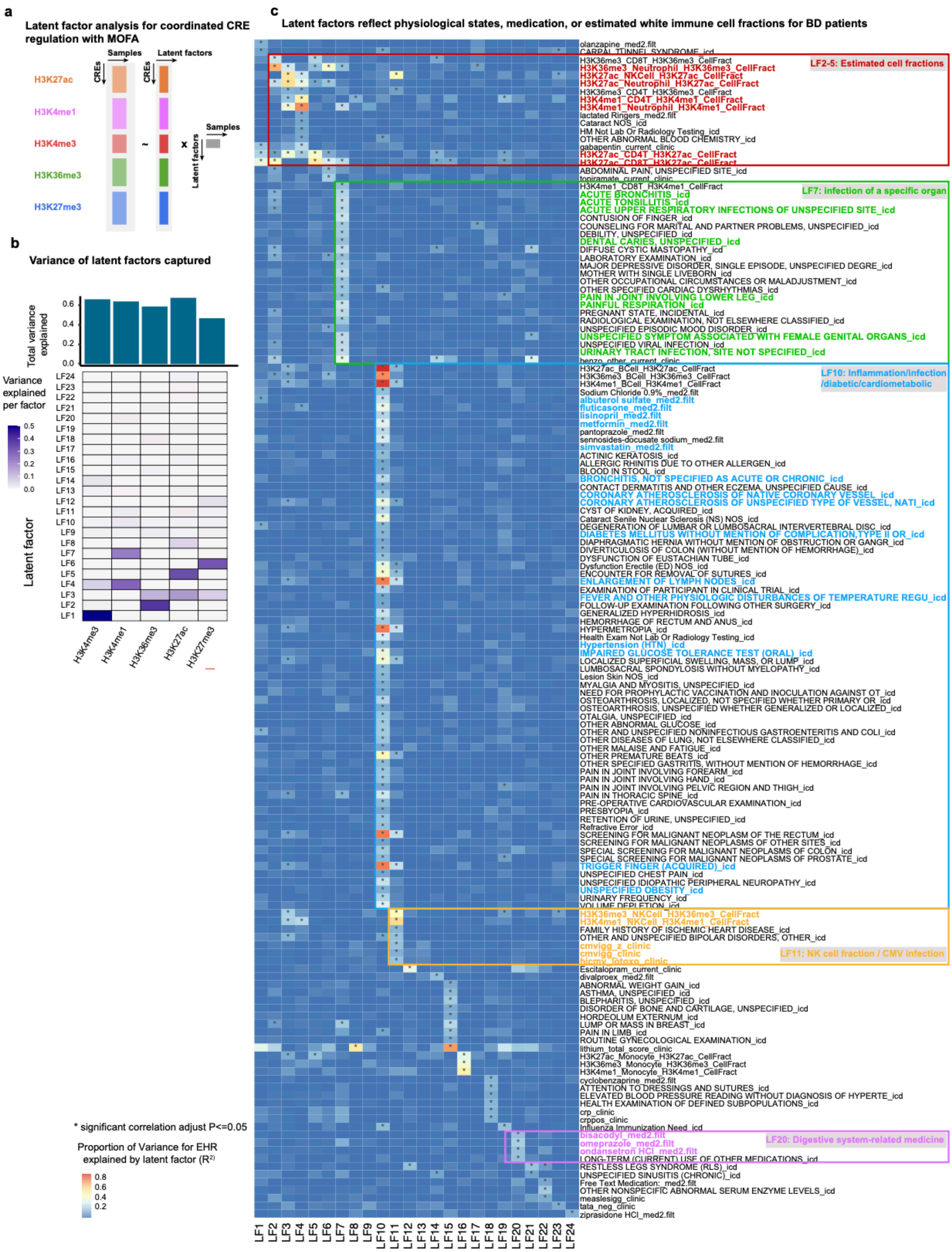

a
Latent factor analysis for coordinated CRE regulation with MOFA
Samples
Latent factors
CREs
H3K27ac
H3K4me1
H3K4me3
H3K36me3
H3K27me3
~
x
Samples
Latent factors
b
Variance of latent factors captured
Total variance explained
0.6
0.4
0.2
0.0
Variance explained per factor
0.5
0.4
0.3
0.2
0.1
0.0
Latent factor
LF24
LF23
LF22
LF21
LF20
LF19
LF18
LF17
LF16
LF15
LF14
LF13
LF12
LF11
LF10
LF9
LF8
LF7
LF6
LF5
LF4
LF3
LF2
LF1
H3K4me3
H3K4me1
H3K36me3
H3K27ac
H3K27me3
* significant correlation adjust P<=0.05
Proportion of Variance for EHR explained by latent factor (R²)
0.8
0.6
0.4
0.2
c
Latent factors reflect physiological states, medication, or estimated white immune cell fractions for BD patients
LF2-5: Estimated cell fractions
LF7: infection of a specific organ
LF10: Inflammation/infection /diabetic/cardiometabolic
LF11: NK cell fraction / CMV infection
LF20: Digestive system-related medicine
olanzapine_med2.filt
CARPAL TUNNEL SYNDROME_icd
H3K36me3_CD8T_H3K36me3_CellFract
H3K36me3_Neutrophil_H3K36me3_CellFract
H3K27ac_NKCell_H3K27ac_CellFract
H3K27ac_Neutrophil_H3K27ac_CellFract
H3K36me3_CD4T_H3K36me3_CellFract
H3K4me1_CD4T_H3K4me1_CellFract
H3K4me1_Neutrophil_H3K4me1_CellFract
lactated Ringers_med2.filt
Cataract NOS_icd
HM Not Lab Or Radiology Testing_icd
OTHER ABNORMAL BLOOD CHEMISTRY_icd
gabapentin_current_clinic
H3K27ac_CD4T_H3K27ac_CellFract
H3K27ac_CD8T_H3K27ac_CellFract
ABDOMINAL PAIN, UNSPECIFIED SITE_icd
topiramate_current_clinic
H3K4me1_CD8T_H3K4me1_CellFract
ACUTE BRONCHITIS_icd
ACUTE TONSILLITIS_icd
ACUTE UPPER RESPIRATORY INFECTIONS OF UNSPECIFIED SITE_icd
CONTUSION OF FINGER_icd
COUNSELING FOR MARITAL AND PARTNER PROBLEMS, UNSPECIFIED_icd
DEBILITY, UNSPECIFIED_icd
DENTAL CARIES, UNSPECIFIED_icd
DIFFUSE CYSTIC MASTOPATHY_icd
LABORATORY EXAMINATION_icd
MAJOR DEPRESSIVE DISORDER, SINGLE EPISODE, UNSPECIFIED DEGRE_icd
MOTHER WITH SINGLE LIVEBORN_icd
OTHER OCCUPATIONAL CIRCUMSTANCES OR MALADJUSTMENT_icd
OTHER SPECIFIED CARDIAC DYSRHYTHMIAS_icd
PAIN IN JOINT INVOLVING LOWER LEG_icd
PAINFUL RESPIRATION_icd
PREGNANT STATE, INCIDENTAL_icd
RADIOLOGICAL EXAMINATION, NOT ELSEWHERE CLASSIFIED_icd
UNSPECIFIED EPISODIC MOOD DISORDER_icd
UNSPECIFIED SYMPTOM ASSOCIATED WITH FEMALE GENITAL ORGANS_icd
UNSPECIFIED VIRAL INFECTION_icd
URINARY TRACT INFECTION, SITE NOT SPECIFIED_icd
benzo_other_current_clinic
H3K27ac_BCell_H3K27ac_CellFract
H3K36me3_BCell_H3K36me3_CellFract
H3K4me1_BCell_H3K4me1_CellFract
Sodium Chloride 0.9%_med2.filt
albuterol sulfate_med2.filt
fluticasone_med2.filt
lisinopril_med2.filt
metformin_med2.filt
pantoprazole_med2.filt
sennosides-docusate sodium_med2.filt
simvastatin_med2.filt
ACTINIC KERATOSIS_icd
ALLERGIC RHINITIS DUE TO OTHER ALLERGEN_icd
BLOOD IN STOOL_icd
BRONCHITIS, NOT SPECIFIED AS ACUTE OR CHRONIC_icd
CONTACT DERMATITIS AND OTHER ECZEMA, UNSPECIFIED CAUSE_icd
CORONARY ATHEROSCLEROSIS OF NATIVE CORONARY VESSEL_icd
CORONARY ATHEROSCLEROSIS OF UNSPECIFIED TYPE OF VESSEL, NATI_icd
CYST OF KIDNEY, ACQUIRED_icd
Cataract Senile Nuclear Sclerosis (NS) NOS_icd
DEGENERATION OF LUMBAR OR LUMBOSACRAL INTERVERTEBRAL DISC_icd
DIABETES MELLITUS WITHOUT MENTION OF COMPLICATION,TYPE II OR_icd
DIAPHRAGMATIC HERNIA WITHOUT MENTION OF OBSTRUCTION OR GANGR_icd
DIVERTICULOSIS OF COLON (WITHOUT MENTION OF HEMORRHAGE)_icd
DYSFUNCTION OF EUSTACHIAN TUBE_icd
Dysfunction Erectile (ED) NOS_icd
ENCOUNTER FOR REMOVAL OF SUTURES_icd
ENLARGEMENT OF LYMPH NODES_icd
EXAMINATION OF PARTICIPANT IN CLINICAL TRIAL_icd
FEVER AND OTHER PHYSIOLOGIC DISTURBANCES OF TEMPERATURE REGU_icd
FOLLOW-UP EXAMINATION FOLLOWING OTHER SURGERY_icd
GENERALIZED HYPERHIDROSIS_icd
HEMORRHAGE OF RECTUM AND ANUS_icd
HYPERMETROPIA_icd
Health Exam Not Lab Or Radiology Testing_icd
Hypertension (HTN)_icd
IMPAIRED GLUCOSE TOLERANCE TEST (ORAL)_icd
LOCALIZED SUPERFICIAL SWELLING, MASS, OR LUMP_icd
LUMBOSACRAL SPONDYLOSIS WITHOUT MYELOPATHY_icd
Lesion Skin NOS_icd
MYALGIA AND MYOSITIS, UNSPECIFIED_icd
NEED FOR PROPHYLACTIC VACCINATION AND INOCULATION AGAINST OT_icd
OSTEOARTHROSIS, LOCALIZED, NOT SPECIFIED WHETHER PRIMARY OR_icd
OSTEOARTHROSIS, UNSPECIFIED WHETHER GENERALIZED OR LOCALIZED_icd
OTALGIA, UNSPECIFIED_icd
OTHER ABNORMAL GLUCOSE_icd
OTHER AND UNSPECIFIED NONINFECTIOUS GASTROENTERITIS AND COLI_icd
OTHER DISEASES OF LUNG, NOT ELSEWHERE CLASSIFIED_icd
OTHER MALAISE AND FATIGUE_icd
OTHER PREMATURE BEATS_icd
OTHER SPECIFIED GASTRITIS, WITHOUT MENTION OF HEMORRHAGE_icd
PAIN IN JOINT INVOLVING FOREARM_icd
PAIN IN JOINT INVOLVING HAND_icd
PAIN IN JOINT INVOLVING PELVIC REGION AND THIGH_icd
PAIN IN THORACIC SPINE_icd
PRE-OPERATIVE CARDIOVASCULAR EXAMINATION_icd
PRESBYOPIA_icd
RETENTION OF URINE, UNSPECIFIED_icd
Refractive Error_icd
SCREENING FOR MALIGNANT NEOPLASM OF THE RECTUM_icd
SCREENING FOR MALIGNANT NEOPLASMS OF OTHER SITES_icd
SPECIAL SCREENING FOR MALIGNANT NEOPLASMS OF COLON_icd
SPECIAL SCREENING FOR MALIGNANT NEOPLASMS OF PROSTATE_icd
TRIGGER FINGER (ACQUIRED)_icd
UNSPECIFIED CHEST PAIN_icd
UNSPECIFIED IDIOPATHIC PERIPHERAL NEUROPATHY_icd
UNSPECIFIED OBESITY_icd
URINARY FREQUENCY_icd
VOLUME DEPLETION_icd
H3K36me3_NKCell_H3K36me3_CellFract
H3K4me1_NKCell_H3K4me1_CellFract
FAMILY HISTORY OF ISCHEMIC HEART DISEASE_icd
OTHER AND UNSPECIFIED BIPOLAR DISORDERS, OTHER_icd
cmvigg_z_clinic
cmvigg_clinic
hicmv_totoxo_clinic
Escitalopram_current_clinic
divalproex_med2.filt
ABNORMAL WEIGHT GAIN_icd
ASTHMA, UNSPECIFIED_icd
BLEPHARITIS, UNSPECIFIED_icd
DISORDER OF BONE AND CARTILAGE, UNSPECIFIED_icd
HORDEOLUM EXTERNUM_icd
LUMP OR MASS IN BREAST_icd
PAIN IN LIMB_icd
ROUTINE GYNECOLOGICAL EXAMINATION_icd
lithium_total_score_clinic
H3K27ac_Monocyte_H3K27ac_CellFract
H3K36me3_Monocyte_H3K36me3_CellFract
H3K4me1_Monocyte_H3K4me1_CellFract
cyclobenzaprine_med2.filt
ATTENTION TO DRESSINGS AND SUTURES_icd
ELEVATED BLOOD PRESSURE READING WITHOUT DIAGNOSIS OF HYPERTE_icd
HEALTH EXAMINATION OF DEFINED SUBPOPULATIONS_icd
crp_clinic
crppos_clinic
Influenza Immunization Need_icd
bisacodyl_med2.filt
omeprazole_med2.filt
ondansetron HCl_med2.filt
LONG-TERM (CURRENT) USE OF OTHER MEDICATIONS_icd
RESTLESS LEGS SYNDROME (RLS)_icd
UNSPECIFIED SINUSITIS (CHRONIC)_icd
Free Text Medication_med2.filt
OTHER NONSPECIFIC ABNORMAL SERUM ENZYME LEVELS_icd
measlesigg_clinic
tata_neg_clinic
ziprasidone HCl_med2.filt
LF1 LF2 LF3 LF4 LF5 LF6 LF7 LF8 LF9 LF10 LF11 LF12 LF13 LF14 LF15 LF16 LF17 LF18 LF19 LF20 LF21 LF22 LF23 LF24

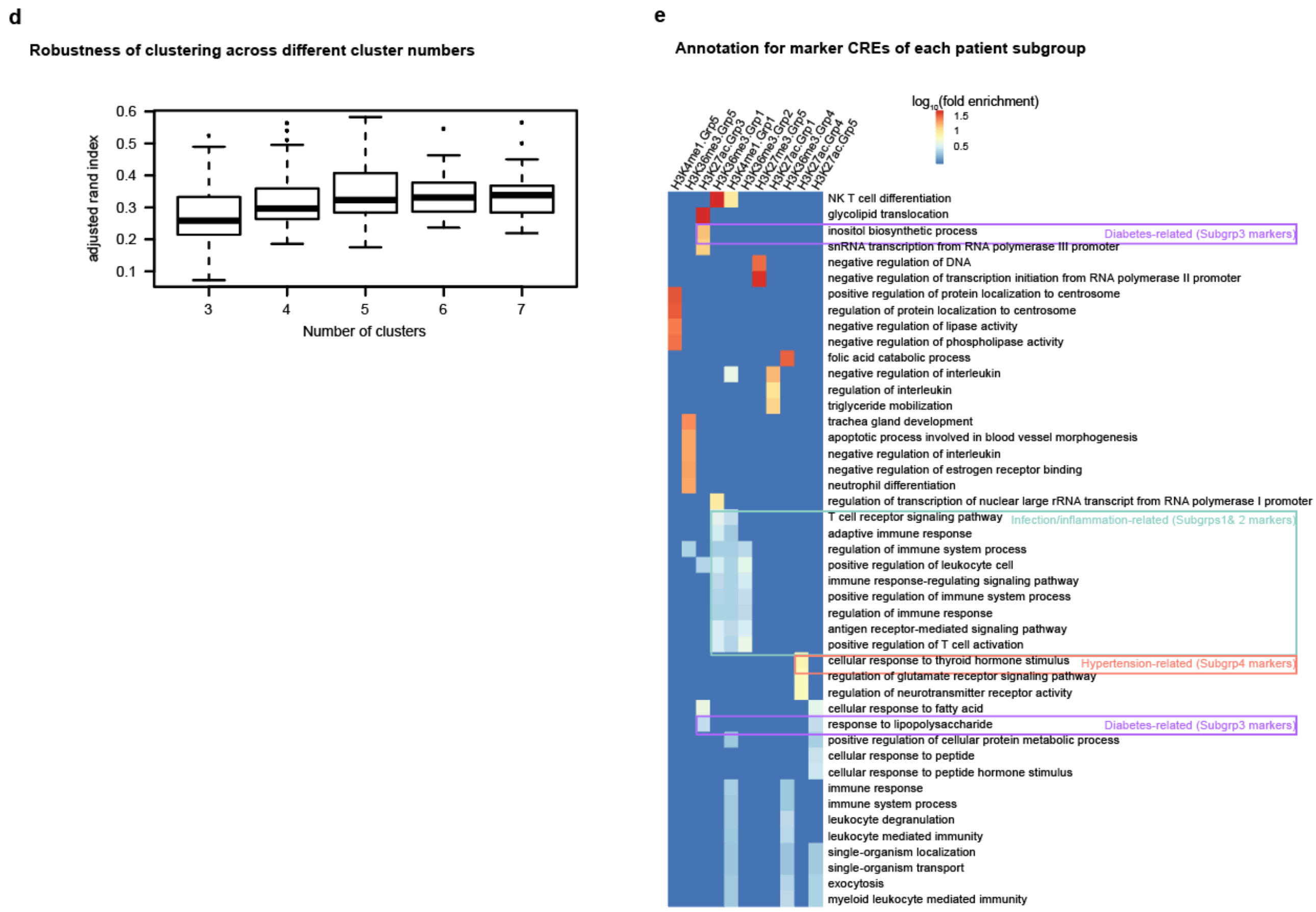

Supplementary Figure 8. Latent factor analysis, cluster, and marker CREs. a Latent factor analysis for coordinated CRE regulation with MOFA. A schematic diagram showing how latent factor analysis based on MOFA works. **b** Variance of latent factors captured. Top: the bar plot shows the total variance explained by all latent factors (y-axis) for each histone mark (x-axis); bottom: the heatmap shows the variance explained for each factor (by row). **c** Latent factors reflect physiological states, medication, or estimated white immune cell fractions for BD patients. The heatmap shows the proportion of variance for each covariate/EHR term (by row) explained by each latent factor (by column), with covariates/terms associated with several latent factors highlighted on the right. Significant levels are shown based on adjusted *P* values (two-sided *t*-test). **d** Robustness of clustering across different cluster numbers. Boxplots show how adjusted rand index changes with preset number of clusters. **e** Annotation of marker CREs of each patient group. The heatmap shows the enrichment of GO terms (by row) for each marker CRE set identified for each patient group (by column), with terms consistent with prior knowledge highlighted on the right. Source data are provided as a Source Data file.

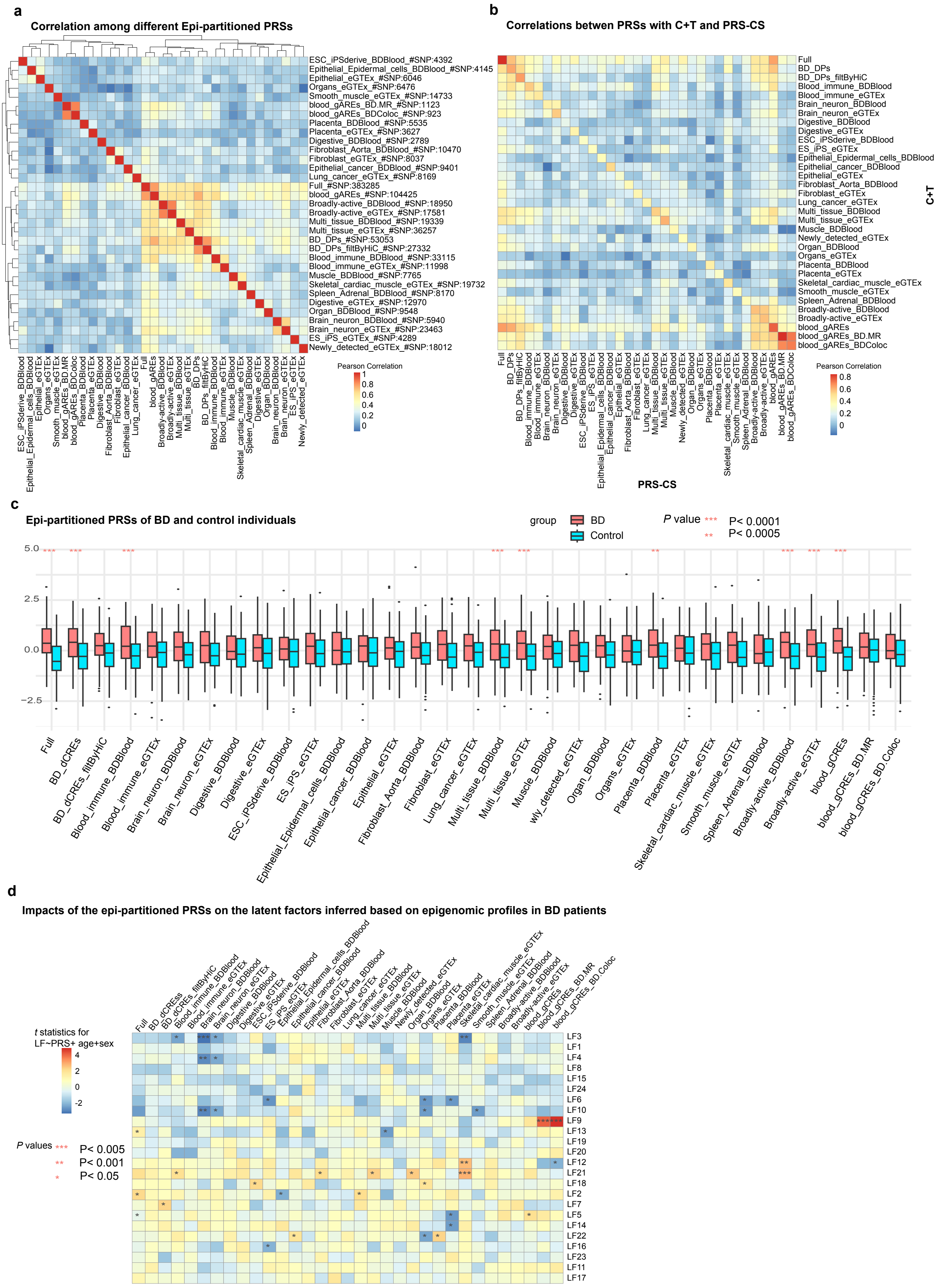

a
Correlation among different Epi-partitioned PRSs
ESC_iPSderive_BDBlood_#SNP:4392
Epithelial_Epidermal_cells_BDBlood_#SNP:4145
Epithelial_eGTEx_#SNP:6046
Organs_eGTEx_#SNP:6476
Smooth_muscle_eGTEx_#SNP:14733
blood_gAREs_BD.MR_#SNP:1123
blood_gAREs_BDColoc_#SNP:923
Placenta_BDBlood_#SNP:5535
Placenta_eGTEx_#SNP:3627
Digestive_BDBlood_#SNP:2789
Fibroblast_Aorta_BDBlood_#SNP:10470
Fibroblast_eGTEx_#SNP:8037
Epithelial_cancer_BDBlood_#SNP:9401
Lung_cancer_eGTEx_#SNP:8169
Full_#SNP:383285
blood_gAREs_#SNP:104425
Broadly-active_BDBlood_#SNP:18950
Broadly-active_eGTEx_#SNP:17581
Multi_tissue_BDBlood_#SNP:19339
Multi_tissue_eGTEx_#SNP:36257
BD_DPs_#SNP:53053
BD_DPs_filtByHiC_#SNP:27332
Blood_immune_BDBlood_#SNP:33115
Blood_immune_eGTEx_#SNP:11998
Muscle_BDBlood_#SNP:7765
Skeletal_cardiac_muscle_eGTEx_#SNP:19732
Spleen_Adrenal_BDBlood_#SNP:8170
Digestive_eGTEx_#SNP:12970
Organ_BDBlood_#SNP:9548
Brain_neuron_BDBlood_#SNP:5940
Brain_neuron_eGTEx_#SNP:23463
ES_iPS_eGTEx_#SNP:4289
Newly_detected_eGTEx_#SNP:18012
Pearson Correlation
1
0.8
0.6
0.4
0.2
0
b
Correlations betwen PRSs with C+T and PRS-CS
Full
BD_DPs
BD_DPs_filtByHiC
Blood_immune_BDBlood
Blood_immune_eGTEx
Brain_neuron_BDBlood
Brain_neuron_eGTEx
Digestive_BDBlood
Digestive_eGTEx
ESC_iPSderive_BDBlood
ES_iPS_eGTEx
Epithelial_Epidermal_cells_BDBlood
Epithelial_cancer_BDBlood
Epithelial_eGTEx
Fibroblast_Aorta_BDBlood
Fibroblast_eGTEx
Lung_cancer_eGTEx
Multi_tissue_BDBlood
Multi_tissue_eGTEx
Muscle_BDBlood
Newly_detected_eGTEx
Organ_BDBlood
Organs_eGTEx
Placenta_BDBlood
Placenta_eGTEx
Skeletal_cardiac_muscle_eGTEx
Smooth_muscle_eGTEx
Spleen_Adrenal_BDBlood
Broadly-active_BDBlood
Broadly-active_eGTEx
blood_gAREs
blood_gAREs_BD.MR
blood_gAREs_BDColoc
C+T
PRS-CS
Pearson Correlation
0.8
0.6
0.4
0.2
0
c
Epi-partitioned PRSs of BD and control individuals
group
BD
Control
P value *** P< 0.0001
** P< 0.0005
5.0
2.5
0.0
−2.5
Full
BD_dCREs
BD_dCREs_filtByHiC
Blood_immune_BDBlood
Blood_immune_eGTEx
Brain_neuron_BDBlood
Brain_neuron_eGTEx
Digestive_BDBlood
Digestive_eGTEx
ESC_iPSderive_BDBlood
ES_iPS_eGTEx
Epithelial_Epidermal_cells_BDBlood
Epithelial_cancer_BDBlood
Epithelial_eGTEx
Fibroblast_Aorta_BDBlood
Fibroblast_eGTEx
Lung_cancer_eGTEx
Multi_tissue_BDBlood
Multi_tissue_eGTEx
Muscle_BDBlood
wly_detected_eGTEx
Organ_BDBlood
Organs_eGTEx
Placenta_BDBlood
Placenta_eGTEx
Skeletal_cardiac_muscle_eGTEx
Smooth_muscle_eGTEx
Spleen_Adrenal_BDBlood
Broadly-active_BDBlood
Broadly-active_eGTEx
blood_gCREs
blood_gCREs_BD.MR
blood_gCREs_BD.Coloc
d
Impacts of the epi-partitioned PRSs on the latent factors inferred based on epigenomic profiles in BD patients
t statistics for
LF~PRS+ age+sex
4
2
0
-2
P values *** P< 0.005
** P< 0.001
* P< 0.05
LF3
LF1
LF4
LF8
LF15
LF24
LF6
LF10
LF9
LF13
LF19
LF20
LF12
LF21
LF18
LF2
LF7
LF5
LF14
LF22
LF16
LF23
LF11
LF17

Supplementary Figure 9. PRS for BD subgroups. **a** Correlation of Epi-partitioned PRSs across all individuals. The heatmap shows the person correlation with SNP numbers for each PRS included in the row names. **b** Correlation of Epi-partitioned PRSs based on Clumping + threshold (C+T) strategy and PRS-CS. The heatmap shows the person correlation with PRS based on C+T on each row and PRS based on PRS-CS on each column. **c** Epi-partitioned PRSs of BD and control patients (red and blue box plots, respectively). The x-axis represents different scores; the y-axis shows z scores for each PRS for each individual. Boxes: 25%-75% percentile (i.e. inter-quartile range; IQR); line: median; whiskers: 1.5 IQR. Asterisks indicate the significance levels of the difference of scores between the two groups. **d** Impacts of the epi-partitioned PRSs on the LFs inferred based on the epigenomic profiles of BD patients. Color of the heatmap represents the t statistics of a PRS in a linear regression model to predict a latent factor correcting for age and sex; each row of the heatmap represents a latent factor; and each column represents a PRS, with asterisks indicating the significance levels of the t statistics (two-sided t-test). Source data are provided as a Source Data file.

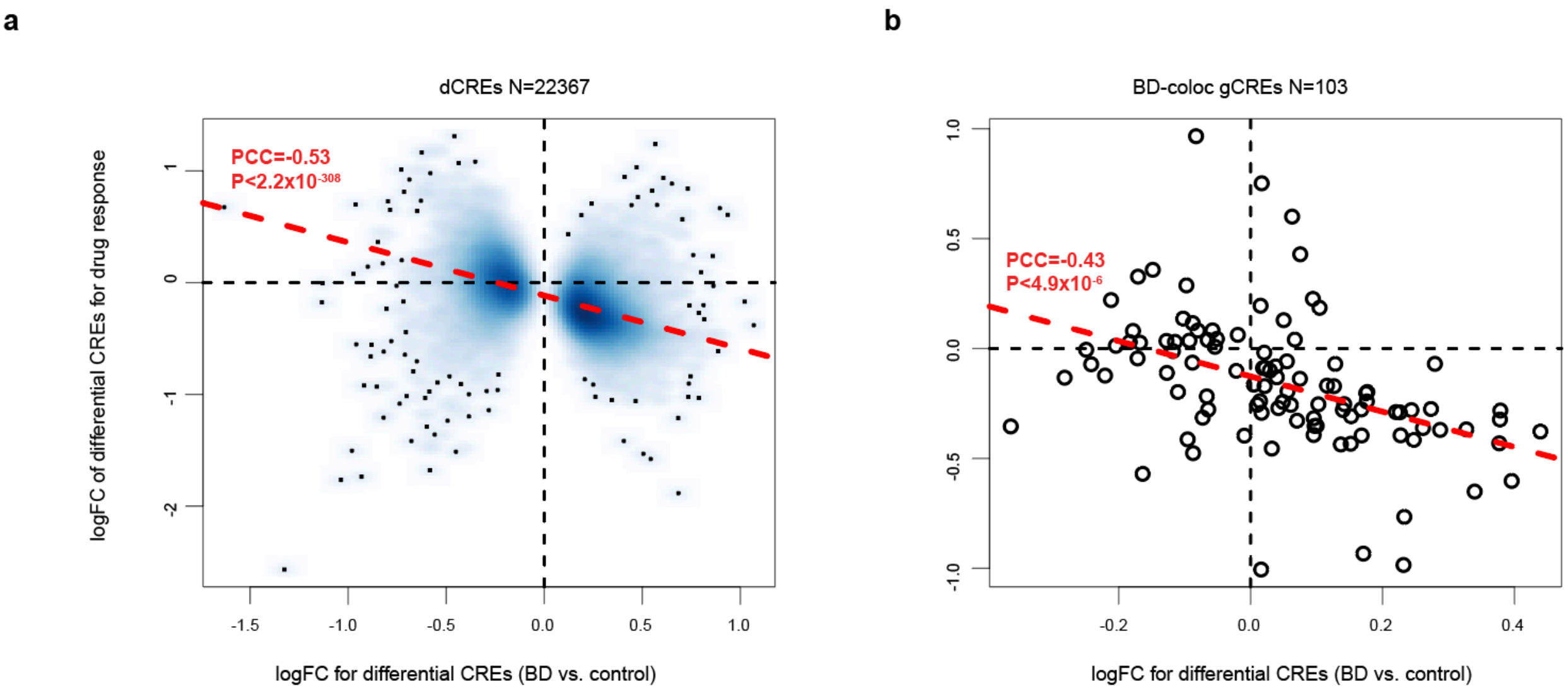

Supplementary Figure 10. Gabapentin reverses the differential activity of H3K27ac CREs associated with BD. Scatterplots show the log fold change of CRE activity associated with Gabapentin (Y-axis) vs. that associated with BD (X-axis) for dCREs (**panel a**) and BD GWAS-colocalized gCREs (**panel b**). Red dashed lines represent the regression lines. P values show the significance of correlation (two-sided). Source data are provided as a Source Data file.

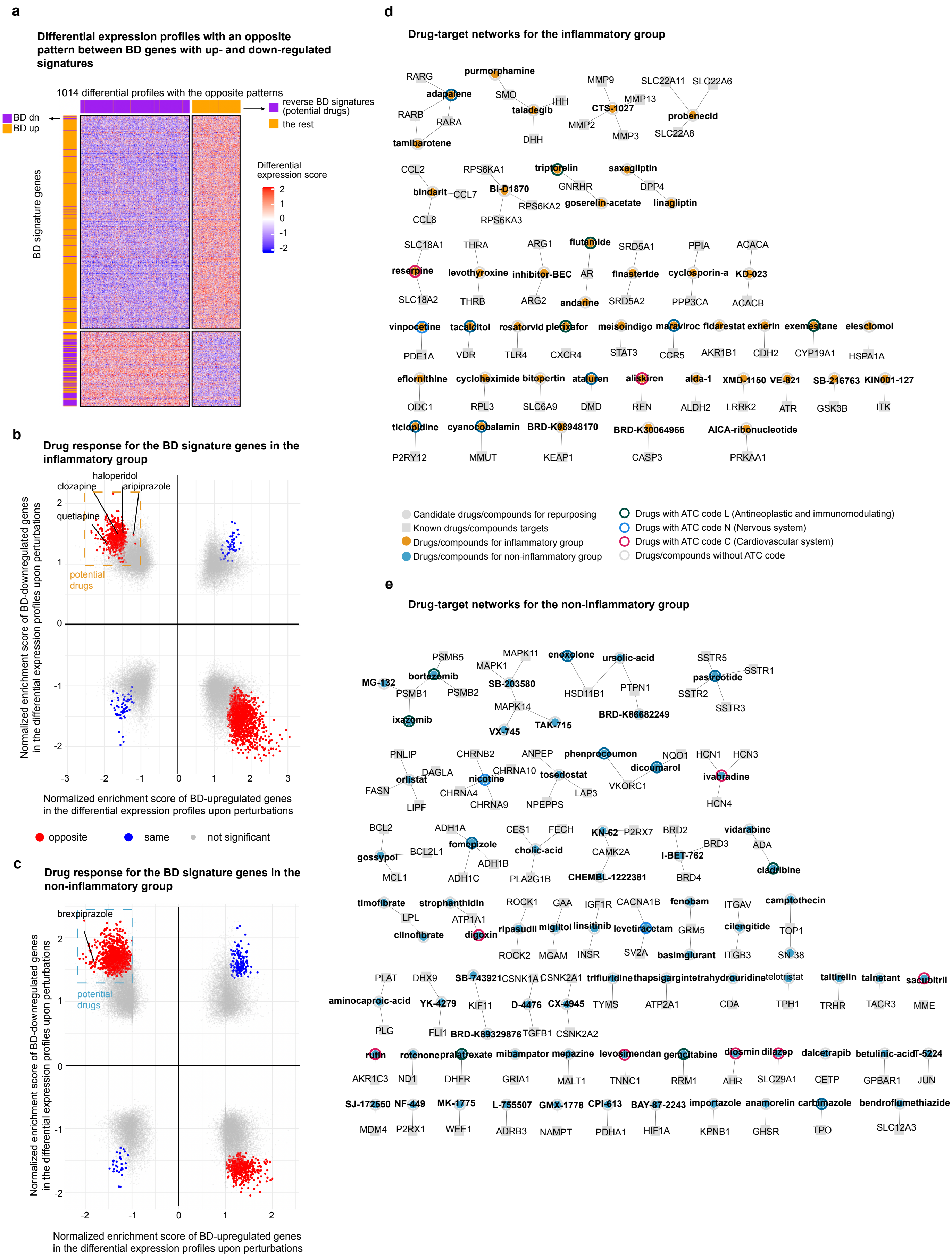

a
Differential expression profiles with an opposite pattern between BD genes with up- and down-regulated signatures
1014 differential profiles with the opposite patterns
reverse BD signatures (potential drugs)
the rest
BD dn
BD up
BD signature genes
Differential expression score
2
1
0
-1
-2
b
Drug response for the BD signature genes in the inflammatory group
haloperidol
clozapine
aripiprazole
quetiapine
potential drugs
Normalized enrichment score of BD-downregulated genes in the differential expression profiles upon perturbations
Normalized enrichment score of BD-upregulated genes in the differential expression profiles upon perturbations
opposite
same
not significant
c
Drug response for the BD signature genes in the non-inflammatory group
brexpiprazole
potential drugs
Normalized enrichment score of BD-downregulated genes in the differential expression profiles upon perturbations
Normalized enrichment score of BD-upregulated genes in the differential expression profiles upon perturbations
d
Drug-target networks for the inflammatory group
RARG
purmorphamine
MMP9
SLC22A11
SLC22A6
adapalene
SMO
IHH
MMP13
RARB
taladegib
CTS-1027
probenecid
RARA
MMP2
MMP3
SLC22A8
tamibarotene
DHH
CCL2
RPS6KA1
triptorelin
saxagliptin
bindarit
CCL7
BI-D1870
GNRHR
DPP4
RPS6KA2
goserelin-acetate
linagliptin
CCL8
RPS6KA3
SLC18A1
THRA
ARG1
flutamide
SRD5A1
PPIA
ACACA
reserpine
levothyroxine
inhibitor-BEC
AR
finasteride
cyclosporin-a
KD-023
SLC18A2
THRB
ARG2
andarine
SRD5A2
PPP3CA
ACACB
vinpocetine
tacalcitol
resatorvid
plerixafor
meisoindigo
maraviroc
fidarestat
exherin
exemestane
elesclomol
PDE1A
VDR
TLR4
CXCR4
STAT3
CCR5
AKR1B1
CDH2
CYP19A1
HSPA1A
eflornithine
cycloheximide
bitopertin
ataluren
aliskiren
alda-1
XMD-1150
VE-821
SB-216763
KIN001-127
ODC1
RPL3
SLC6A9
DMD
REN
ALDH2
LRRK2
ATR
GSK3B
ITK
ticlopidine
cyanocobalamin
BRD-K98948170
BRD-K30064966
AICA-ribonucleotide
P2RY12
MMUT
KEAP1
CASP3
PRKAA1
Candidate drugs/compounds for repurposing
Known drugs/compounds targets
Drugs/compounds for inflammatory group
Drugs/compounds for non-inflammatory group
Drugs with ATC code L (Antineoplastic and immunomodulating)
Drugs with ATC code N (Nervous system)
Drugs with ATC code C (Cardiovascular system)
Drugs/compounds without ATC code
e
Drug-target networks for the non-inflammatory group
PSMB5
MAPK11
enoxolone
ursolic-acid
SSTR5
MAPK1
SSTR1
MG-132
bortezomib
SB-203580
pasireotide
PSMB1
PSMB2
HSD11B1
PTPN1
SSTR2
MAPK14
BRD-K86682249
SSTR3
ixazomib
TAK-715
VX-745
PNLIP
CHRNB2
ANPEP
phenprocoumon
NQO1
HCN1
HCN3
DAGLA
CHRNA10
orlistat
nicotine
tosedostat
dicoumarol
ivabradine
FASN
CHRNA4
LAP3
VKORC1
LIPF
CHRNA9
NPEPPS
HCN4
BCL2
ADH1A
CES1
FECH
KN-62
P2RX7
BRD2
vidarabine
fomepizole
BRD3
ADA
gossypol
BCL2L1
cholic-acid
CAMK2A
I-BET-762
ADH1B
cladribine
MCL1
ADH1C
PLA2G1B
CHEMBL-1222381
BRD4
timofibrate
strophanthidin
ROCK1
GAA
IGF1R
CACNA1B
fenobam
ITGAV
camptothecin
LPL
ATP1A1
clinofibrate
digoxin
ripasudil
miglitol
linsitinib
levetiracetam
GRM5
cilengitide
TOP1
ROCK2
MGAM
INSR
SV2A
basimglurant
ITGB3
SN-38
PLAT
DHX9
SB-743921
CSNK1A1
CSNK2A1
trifluridine
thapsigargin
tetrahydrouridine
telotristat
taltirelin
talnetant
sacubitril
aminocaproic-acid
YK-4279
KIF11
D-4476
CX-4945
TYMS
ATP2A1
CDA
TPH1
TRHR
TACR3
MME
PLG
FLI1
BRD-K89329876
TGFB1
CSNK2A2
rutin
rotenone
pralatrexate
mibampator
mepazine
levosimendan
gemcitabine
diosmin
dilazep
dalcetrapib
betulinic-acid
T-5224
AKR1C3
ND1
DHFR
GRIA1
MALT1
TNNC1
RRM1
AHR
SLC29A1
CETP
GPBAR1
JUN
SJ-172550
NF-449
MK-1775
L-755507
GMX-1778
CPI-613
BAY-87-2243
importazole
anamorelin
carbimazole
bendroflumethiazide
MDM4
P2RX1
WEE1
ADRB3
NAMPT
PDHA1
HIF1A
KPNB1
GHSR
TPO
SLC12A3

Supplementary Figure 11. Drug response and repurposing. a Differential expression profiles in response to pharmacological interventions (from CMap) with an opposite enrichment pattern for BD genes with up- and down-regulated epigenomic signatures. Rows represent 1014 expression profiles, while columns represent the top 500 differentially expressed genes with BD up-/down-regulated epigenomic signatures. **b**, Enrichment of genes with BD up/down-regulated epi-signatures in the differential expression profiles of hematopoietic cell lines exposed to pharmacological perturbations for the inflammatory subgroups (subgroup 1 and 2 in Figure 5c). BD epi-signatures were identified by comparing the BD patients from inflammatory subgroups to all the controls. Each dot represents an expression profile upon a perturbation; the x-axis and y-axis represent normalized enrichment scores (NES) for the genes with BD up-regulated and down-regulated epi-signatures, respectively. Colored dots show differential expression profiles with significant NES for both gene sets (nominal $P<0.001$), with red dots representing changes with opposite signs of enrichment for BD up- and down-regulated genes and blue dots representing changes with the same signs of enrichment. **c** The same plot as **panel b** except that it is for the non-inflammatory subgroups (subgroup 3-5 in **Figure 5c**). **d & e** Network view of repurposed drugs and their targets specifically for inflammatory (**d**) and non-inflammatory (**e**) patient groups. Nodes represent either drugs (square) or targets (circle) with labels and edges connecting drugs to their targets. Source data are provided as a Source Data file.

Supplementary Tables

Supplementary Table 1. Antibodies used in the chromatin immunoprecipitation-sequencing (ChIP-seq) studies

Target	Supplier	Host	Clone	ID	Isotype	Final conc.	Validation source
H3K27ac	CST	Rabbit	mc	8173	IgG	2µg/4 x 10^6 cells	https://www.cellsignal.com/products/primary-antibodies/histone-h3-acetyl-lys27-d5e4-xp-rabbit-mab/8173
H3K4me1	CST	Rabbit	mc	5326	IgG	2µg/4 x 10^6 cells	https://www.cellsignal.com/products/primary-antibodies/histone-h3-monmethyl-lys4-d1a9-xp-rabbit-mab/5326
H3K4me3	EDL	Rabbit	pc	EDL Lot1	IgG	2µg/4 x 10^6 cells	PMID: 29268714
H3K27me3	CST	Rabbit	mc	9733	IgG	2µg/4 x 10^6 cells	https://www.cellsignal.com/products/primary-antibodies/histone-h3-trimethyl-lys27-c36b11-rabbit-mab/9733
H3K36me3	Active Motif	Rabbit	pc	61101	IgG	2µg/4 x 10^6 cells	https://www.activemotif.com/catalog/details/61101/histone-h3-trimethyl-lys36-antibody-pab

Abbreviations: Suppliers: CST, Cell Signaling Technology, Inc., Beverly, MA, USA; EDL, Epigenomics Development Laboratory, Mayo Clinic Center for Individualized Medicine, Rochester, MN; Active Motif, Active Motif, Inc., Carlsbad, CA. Host: mc, monoclonal; pc, polyclonal.

Supplementary Table 2. Counts of repurposed drugs based on ATC code shared and specific for each group of patients (**Figure 6c**, **Supplementary Figure 11d-e**). A: Alimentary tract and metabolism; B: Blood and blood-forming organs; C: Cardiovascular system; D: Dermatologicals; G: Genito-urinary system and sex hormones; H: Systemic hormonal preparations; J: Antiinfectives for systemic use; L: Antineoplastic and immunomodulating agents; M: Musculo-skeletal system; N: Nervous system; P: Antiparasitic products, insecticides, and repellents; R: Respiratory system; S: Sensory organs; V: Various

	**shared**	**inflammatory group**	**non-inflammatory group**
A	16	2	3
B	4	2	2
**C**	**19**	**2**	**7**
D	4	2	1
G	6	0	0
H	1	0	2
J	3	1	0
**L**	**48**	**4**	**5**
M	4	1	0
**N**	**30**	**1**	**2**
P	2	0	0
R	2	0	0
S	1	0	1
V	1	0	1
**sum**	141	15	24
