# Supplementary material for: Genotype epigenome phenotype integration reveals peripheral immune contributions to type I bipolar disorder
Source: Nat Commun. 2026 May 15;17:7740. doi: 10.1038/s41467-026-72543-0 (PMC13434788; doi:10.1038/s41467-026-72543-0)
Supplement: Supplementary file 4 [file 41467_2026_72543_MOESM2_ESM.pdf]

Description of Additional Supplementary Files

Supplementary Data 1. metadata and QC. These sheets include metadata for donors, and QC data for each sample for each histone marks.

Supplementary Data 2. CREs detection. These sheets include cis-regulatory-elements (CREs) detected for each histone mark (in hg19).

Supplementary Data 3. dCREs identification. These sheets are about differential CREs (dCREs), including dCREs related to bipolar disorder (BD) (in hg19), enriched TFs, genes with BD epi-signatures, GO terms enriched for these genes,

Supplementary Data 4. gCREs. These sheets are about genetically driven CREs (gCREs), including gCREs for each histone mark (in hg38), coloc PP4 for shared genetic signal between gCREs and BD GWAS (merged gCREs across histone marks in hg19), and BD GWAS lead SNPs associated with CREs across histone marks by hQTL (in hg38).

Supplementary Data 5. Driver genes. These sheets are about driver genes identified, including detailed evidence supporting all driver genes, and information for 39 driver genes with blood eQTL-GWAS colocalization supported.

Supplementary Data 6. PRSs. This sheet includes polygenetic risk scores (PRSs) generated for all donors with genetic information.

Supplementary Data 7. Drug response and repurposing. This sheet includes polygenetic risk scores (PRSs) generated for all donors with genetic information, including immune related and non-immune related GO terms enriched for CREs associated with drug response, respectively, perturbation ID and compounds identified based on BD signature genes, and BD signature genes identified for each patient group (inflammatory and non-inflammatory groups)
